# BMAL1–HIF2A heterodimer modulates circadian variations of myocardial injury

**DOI:** 10.1038/s41586-025-08898-z

**Published:** 2025-04-23

**Authors:** Wei Ruan, Tao Li, In Hyuk Bang, Jaewoong Lee, Wankun Deng, Xinxin Ma, Cong Luo, Fang Du, Seung-Hee Yoo, Boyun Kim, Jiwen Li, Xiaoyi Yuan, Katherine Figarella, Yu A. An, Yin-Ying Wang, Yafen Liang, Matthew DeBerge, Dongze Zhang, Zhen Zhou, Yanyu Wang, Joshua M. Gorham, Jonathan G. Seidman, Christine E. Seidman, Sary F. Aranki, Ragini Nair, Lei Li, Jagat Narula, Zhongming Zhao, Alemayehu A. Gorfe, Jochen D. Muehlschlegel, Kuang-Lei Tsai, Holger K. Eltzschig

**Affiliations:** 1 Department of Anesthesiology, Critical Care and Pain Medicine, The University of Texas Health Science Center at Houston, McGovern Medical School, Houston, TX USA; 2 Department of Anesthesiology, Second Xiangya Hospital, Central South University, Changsha, China; 3 Department of Biochemistry and Molecular Biology, The University of Texas Health Science Center at Houston, McGovern Medical School, Houston, TX USA; 4 Department of Anesthesiology, Yale University School of Medicine, New Haven, CT USA; 5 Center for Precision Health, McWilliams School of Biomedical Informatics, The University of Texas Health Science Center at Houston, Houston, TX USA; 6 Department of Anesthesiology, Zhongshan Hospital, Fudan University, Shanghai, China; 7 Major in Aquaculture and Applied Life Sciences, College of Fisheries Science, Pukyong National University, Busan, Republic of Korea; 8 Department of Cardiac Surgery, Sir Run Run Shaw Hospital, School of Medicine, Zhejiang University, Hangzhou, China; 9 Center for Outcomes Research, UTHealth Houston, Houston, TX USA; 10 Division of Medical Genetics, Department of Internal Medicine, The University of Texas Health Science Center at Houston, McGovern Medical School, Houston, TX USA; 11 Department of Genetics, Harvard Medical School, Boston, MA USA; 12 Department of Surgery, Division of Cardiac Surgery, Brigham and Women’s Hospital, Harvard Medical School, Boston, MA USA; 13 Institute of Systems and Physical Biology, Shenzhen Bay Laboratory, Shenzhen, China; 14 Division of Cardiology, Department of Medicine, The University of Texas Health Science Center at Houston, McGovern Medical School, Memorial Hermann Hospital, Houston, TX USA; 15 Department of Integrative Biology and Pharmacology, The University of Texas Health Science Center at Houston, McGovern Medical School, Houston, TX USA; 16 Department of Anesthesiology and Critical Care Medicine, Johns Hopkins University School of Medicine, Baltimore, MD USA; 17 MD Anderson Cancer Center, UTHealth Houston Graduate School of Biomedical Sciences, Houston, TX USA

**Keywords:** Myocardial infarction, Translational research, Cryoelectron microscopy, Randomized controlled trials, Circadian rhythms

## Abstract

Acute myocardial infarction is a leading cause of morbidity and mortality worldwide^1^. Clinical studies have shown that the severity of cardiac injury after myocardial infarction exhibits a circadian pattern, with larger infarcts and poorer outcomes in patients experiencing morning-onset events^2–7^. However, the molecular mechanisms underlying these diurnal variations remain unclear. Here we show that the core circadian transcription factor BMAL1^7–11^ regulates circadian-dependent myocardial injury by forming a transcriptionally active heterodimer with a non-canonical partner—hypoxia-inducible factor 2 alpha (HIF2A)^12–16^—in a diurnal manner. To substantiate this finding, we determined the cryo-EM structure of the BMAL1–HIF2A–DNA complex, revealing structural rearrangements within BMAL1 that enable cross-talk between circadian rhythms and hypoxia signalling. BMAL1 modulates the circadian hypoxic response by enhancing the transcriptional activity of HIF2A and stabilizing the HIF2A protein. We further identified amphiregulin (AREG)^16,17^ as a rhythmic target of the BMAL1–HIF2A complex, critical for regulating daytime variations of myocardial injury. Pharmacologically targeting the BMAL1–HIF2A–AREG pathway provides cardioprotection, with maximum efficacy when aligned with the pathway’s circadian phase. These findings identify a mechanism governing circadian variations of myocardial injury and highlight the therapeutic potential of clock-based pharmacological interventions for treating ischaemic heart disease.

## Abstract (web summary)

The core circadian transcription factor BMAL1 regulates circadian-dependent myocardial injury.

## Main

Cardiac injury after acute myocardial infarction (MI) exhibits pronounced circadian rhythmicity, with severity and clinical outcomes varying on the basis of the time of onset^2–7^. Circadian rhythms, driven by the Earth’s day–night cycles, synchronize internal biological functions with environmental changes, enabling organisms to adapt to the daily fluctuations^18,19^. At the core of these rhythms are transcription factors, notably BMAL1^7–11^, which forms a heterodimer with CLOCK to regulate clock-controlled genes^8,10,11,20^. Although clinical and preclinical studies have highlighted the importance of circadian rhythms in cardiovascular physiology and diseases^6,7,21–24^, the mechanisms underlying circadian variations in myocardial injury remain poorly understood. This knowledge gap poses challenges to therapeutic efficacy and may contribute to suboptimal treatment outcomes^21,25^. Here we identify a molecular mechanism in which cross-talk between circadian rhythms and hypoxia signalling^13–15,26–30^ underpins circadian-dependent cardioprotection. Given the universal and essential roles of these pathways across nearly all cells and organs, our findings have broad implications for advancing the understanding and treatment of ischaemic diseases influenced by circadian rhythms.

## Identification of BMAL1 as a regulator

**Fig. 1:**
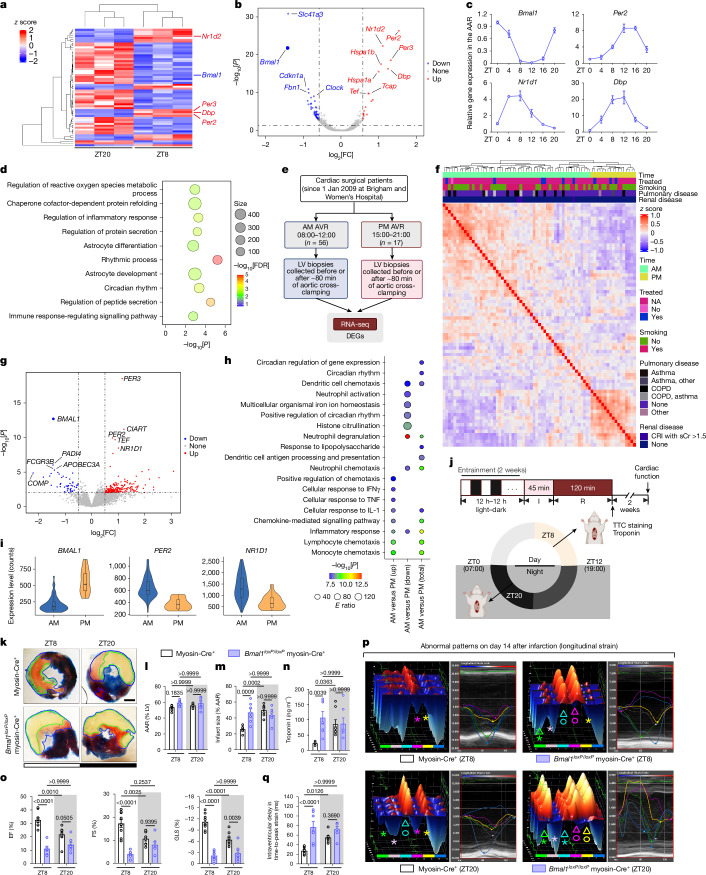
BMAL1 is a key transcription factor in circadian variations of myocardial injury. **a**–**d**, RNA-seq analysis of the AAR from C57BL/6J mice after 2 h reperfusion at ZT8 or ZT20, *n* = 3 mice per timepoint. **a**, Expression *z* scores. **b**, The fold change (FC) in expression. **c**, Analysis of circadian expression of DEGs using RT–qPCR. **d**, The top ten enriched biological process GO terms. FDR, false-discovery rate. **e**–**i**, RNA-seq analysis of post-clamping LV biopsies from patients who had aortic valve replacement (AVR) surgery in the morning (AM; *n* = 56) or afternoon (PM; *n* = 17). **e**, The study design. **f**, Clustered Pearson’s correlations. CRI, chronic renal insufficiency; sCr, serum creatinine. **g**, The fold change in expression after clamping (morning versus afternoon). **h**, Enriched GO terms. **i**, Normalized read counts for DEGs. The box plots show the median (centre line), interquartile range (box limits), and the minimum to maximum values (whiskers). **j**, The experimental setup for cardiac injury and function assessment in *Bmal1*^*loxP/loxP*^ myosin-Cre^+^ and myosin-Cre^+^ mice subjected to IRI at ZT8 or ZT20. I, ischaemia; R, reperfusion. **k**, Heart slices were stained with Evan’s blue and 2,3,5-triphenyltetrazolium chloride (TTC) after 2 h reperfusion; the infarct area (green) and AAR (blue) are shown. Scale bar, 1 mm. **l**, The AAR as the percentage of the LV. **m**, The infarct size as the percentage of the AAR. *n* = 7 mice per group per timepoint. **n**, Serum troponin I levels. *n* = 7 (*Bmal1*^*loxP/loxP*^ myosin-Cre^+^) and *n* = 8 (myosin-Cre^+^) mice per timepoint. **o**–**q**, Cardiac function on day 14 after MI by STE. **o**, The EF, FS and GLS. **p**, 3D and six-segment longitudinal strain imaging with annotations for reduced contractility (stars), dyskinesis (triangles) and dyssynchrony (circles). Dark blue, anterior base; yellow, anterior mid; magenta, anterior apex; cyan, posterior apex; light pink, posterior mid; green, posterior base. **q**, The intraventricular delay. For **o** and **q**, *n* = 7 (*Bmal1*^*loxP/loxP*^ myosin-Cre^+^) and *n* = 9 (myosin-Cre^+^) mice per timepoint. All samples are biologically independent. For **c**,**l**–**o** and **q**, data are mean ± s.e.m. Statistical analysis was performed using Wald tests (**b** and **g**), two-sided Fisher’s exact tests (**d** and **h**) and two-way analysis of variance (ANOVA; **l**–**o** and **q**). The diagram in **j** was created using BioRender. Source Data

**Extended Data Fig. 1:**
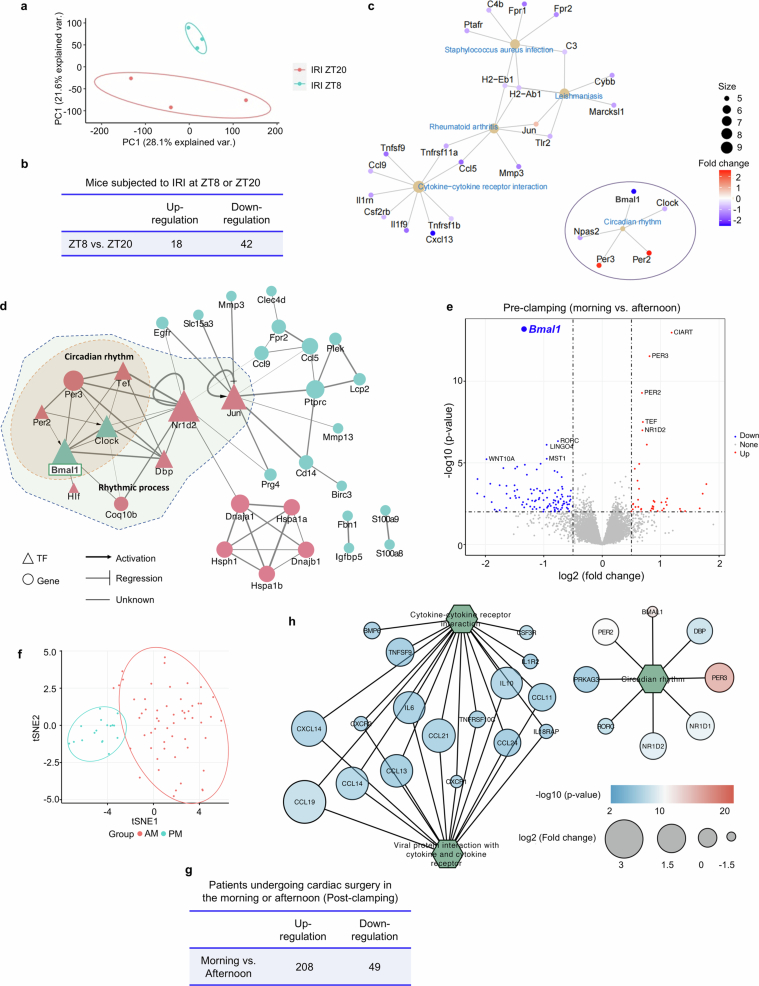
BMAL1 is a key transcription factor in regulating circadian variation of myocardial injury. **a-d**, RNA-seq analysis of the AAR following 2 h of reperfusion in C57BL/6J mice subjected to IRI at ZT8 or ZT20. **a**, PCA of mRNA expression profiles. **b**, Differentially expressed genes (DEGs) identified between ZT8 and ZT20 (fold change > 1.5, adjusted p < 0.05); Wald test. **c**, Top five enriched KEGG pathways for DEGs; two-sided Fisher’s exact test. **d**, Gene dysregulation network showing transcription factors (triangles) and genes (circles), with red and green nodes representing upregulated and downregulated DEGs, respectively, in ZT8 hearts relative to ZT20. Node size indicates degree, and edge width reflects correlation strength. Two-sided Fisher’s exact test. a-d, n = 3 mice/group/time point. **e**, Volcano plot of DEGs in pre-clamping LV biopsies from cardiac surgical patients grouped by surgery time (morning vs. afternoon); Wald test. **f-h**, Human RNA-seq analysis of post-clamping LV biopsies comparing morning (AM) and afternoon (PM) groups. **f**, PCA showing distinct transcriptional signatures between AM and PM groups. **g**, DEGs identified between AM and PM groups (log_2_ fold change > 0.5, p < 0.01); Wald test. **h**, Top three enriched KEGG pathways for DEGs, with node colour indicating p-value and size representing fold change; two-sided Fisher’s exact test. e-h, n = 56 (morning) and n = 17 (afternoon).

Consistent with previous studies^27,31^, diurnal variations in cardiac injury and long-term outcomes were evident in a mouse myocardial ischaemia and reperfusion injury (IRI) model, with the least injury at zeitgeber time 8 (ZT8; 15:00) and the most severe at ZT20 (03:00) (Supplementary Fig. 1). To identify the molecular factors that drive these circadian variations, RNA-sequencing (RNA-seq) was performed on area-at-risk (AAR) samples collected after 2 h of reperfusion at ZT8 and ZT20. This analysis revealed distinct transcriptional profiles, with 18 genes upregulated and 42 downregulated at ZT8 compared with at ZT20 (Extended Data Fig. 1a,b). Notably, BMAL1 target genes, including *Per2*, *Per3*, *Nr1d2* and *Dbp*, were significantly upregulated at ZT8 (Fig. 1a,b and Supplementary Table 1), indicating elevated BMAL1 transcriptional activity. Conversely, *Bmal1* transcript levels were downregulated at ZT8, exhibiting an antiphasic expression pattern relative to its targets, a hallmark of circadian transcription–translation feedback loops^10,11,20^. Analysis using quantitative PCR with reverse transcription (RT–qPCR) confirmed these oscillations (Fig. 1c). Gene Ontology (GO) (Fig. 1d and Supplementary Table 2) and KEGG (Extended Data Fig. 1c) pathway analyses identified ‘circadian rhythm’ as a highly enriched pathway, while a dysregulation network further emphasized BMAL1’s central role in orchestrating diurnal gene expression in ischaemic mouse hearts (Extended Data Fig. 1d).

To investigate transcriptional pathways in human hearts experiencing myocardial injury at different times of the day, we examined left ventricle (LV) biopsy samples from 73 patients undergoing elective aortic valve replacement surgery (NCT00281164). Samples from the morning (*n* = 56, median, 10:32) or afternoon (*n* = 17, median, 17:15) cohort were collected before and after around 80 min of ischaemia induced by aortic cross-clamping (Fig. 1e). Patient demographics and clinical characteristics were comparable between cohorts (Supplementary Table 3). RNA-seq analysis of pre-clamping samples revealed significant circadian modulation of gene expression, with *BMAL1* identified as the most downregulated gene in the morning cohort (Extended Data Fig. 1e and Supplementary Table 4), suggesting its role in the time-of-day-dependent regulation of cardiac physiology, such as metabolism and contractility^6,21–24,32,33^. Post-clamping RNA-seq analysis revealed distinct transcriptional signatures based on surgery timing (Extended Data Fig. 1f), with timing emerging as the dominant determinant over covariates such as smoking or comorbidities (Fig. 1f and Supplementary Table 5). *BMAL1* remained substantially downregulated in the morning cohort (Fig. 1g, Extended Data Fig. 1g and Supplementary Table 6), further implicating it in modulating the heart’s ischaemic response. GO and KEGG analyses revealed significant enrichment in the ‘circadian rhythm’ pathway (Fig. 1h, Extended Data Fig. 1h and Supplementary Table 7), with antiphasic expression patterns of *BMAL1* and its targets paralleling murine observations (Fig. 1i). These findings suggest that the conserved role of BMAL1 as a transcription factor is involved in regulating circadian variations in myocardial injury across species.

## BMAL1 regulates circadian cardiac injury

To further explore BMAL1’s functional role, we generated an inducible cardiomyocyte-specific *Bmal1*-knockout mouse line (*Bmal1*^*loxP/loxP*^*Myh6-cre*; hereafter, *Bmal1*^*loxP/loxP*^ myosin-Cre^+^) by crossing floxed *Bmal1* mice with myosin-Cre^+^ mice and inducing *Bmal1* ablation by tamoxifen injection at 8 weeks of age. We then subjected *Bmal1*^*loxP/loxP*^ myosin-Cre^+^ mice and control myosin-Cre^+^ mice to myocardial IRI at ZT8 or ZT20 (Fig. 1j). After 2 h of reperfusion, *Bmal1-*deficient mice showed abolished circadian variability in myocardial injury and diminished endogenous cardioprotection at ZT8 compared with the controls (Fig. 1k–n). This phenotype persisted during extended reperfusion (day 14 after MI), as speckle-tracking echocardiography (STE)^34,35^ demonstrated that ZT8 *Bmal1-*deficient mice exhibited significantly reduced systolic function (ejection fraction (EF), fractional shortening (FS), global longitudinal strain (GLS)), greater LV dilation (end-diastolic volume and end-systolic volume) and LV mass (end-diastolic LV mass and end-systolic LV mass) (Fig. 1o and Supplementary Fig. 2a). Impaired contractility was evident in both infarcted (anterior mid and posterior apex) and non-infarcted (posterior mid and posterior base) heart segments, indicating more extensive myocardial injury (Supplementary Fig. 2b,c). Pronounced intraventricular disparities further suggested heart failure progression^35^ (Fig. 1p,q). By contrast, IRI at ZT20 showed no significant differences in cardiac function between knockout and control mice. These findings highlight BMAL1’s key role in circadian regulation of acute myocardial injury and long-term outcomes.

## BMAL1 interacts with HIF2A under hypoxia

**Fig. 2:**
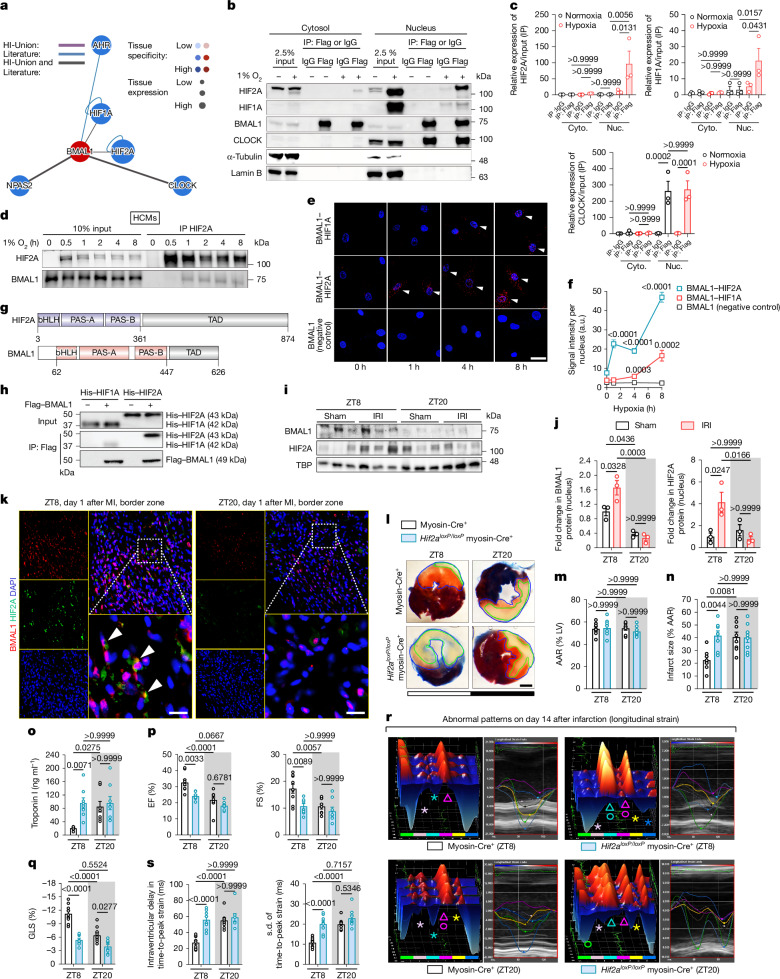
HIF2A interacts with BMAL1 and regulates circadian variations in myocardial injury. **a**, Predicted interactions of BMAL1 with bHLH-PAS transcription factors in the human LV using the HuRI. **b**,**c**, Co-IP analysis (**b**) and quantification (**c**) of BMAL1–Flag in HEK293 cells. *n* = 3 independent experiments. Cyto., cytoplasmic fraction; nuc., nuclear fraction. **d**, Co-IP analysis of HIF2A in HCMs. *n* = 3 independent experiments. **e**,**f**, PLA (**e**) and quantification (**f**) showing BMAL1–HIF2A interactions in HCMs. White arrows indicate the close interaction between BMAL1–HIF2A or BMAL1–HIF1A in the nuclei. Scale bar, 25 μm. *n* = 20 (BMAL1–HIF2A and BMAL1–HIF1A) and *n* = 10 (negative control). **g**, Schematic of BMAL1 and HIF2A protein domains. **h**, Flag pull-down of BMAL1. *n* = 3 independent experiments. **i**,**j**, Western blot analysis (**i**) and quantification (**j**) of nuclear BMAL1 and HIF2A levels in the AAR of C57BL/6J mice after 2 h reperfusion at ZT8 or ZT20. *n* = 3 mice per group per timepoint. Owing to similar molecular masses of proteins, the samples were run on separate gels, with TBP as the sample processing control. **k**, Immunofluorescence analysis of BMAL1 and HIF2A colocalization in the border zone on day 1 after MI, shown in merged images (yellow, white arrows). Scale bars, 25 μm. *n* = 3 mice per group per timepoint. **l**–**o**, *Hif2a*^*loxP/loxP*^ myosin-Cre^+^ mice and myosin-Cre^+^ mice were subjected to IRI at ZT8 or ZT20. Evan’s blue and TTC-stained heart slices (**l**; scale bar, 1 mm), AAR as the percentage of LV (**m**), infarct size as the percentage of the AAR (**n**) and serum troponin I levels (**o**) after 2 h reperfusion are shown. *n* = 8 mice per group per timepoint. **p**–**s**, Cardiac function by day 14 after MI. **p**, The EF and FS. **q**, The GLS. **r**, LV 3D and six-segment longitudinal strain images. **s**, Intraventricular dyssynchrony. For **p**–**s**, *n* = 8 (*Hif2a*^*loxP/loxP*^ myosin-Cre^+^) and *n* = 9 (myosin-Cre^+^) mice per timepoint. Data are mean ± s.e.m. Statistical analysis was performed using one-way ANOVA (**c**) and two-way ANOVA (**f**,**j**,**m**–**q** and **s**). Source Data

**Extended Data Fig. 2:**
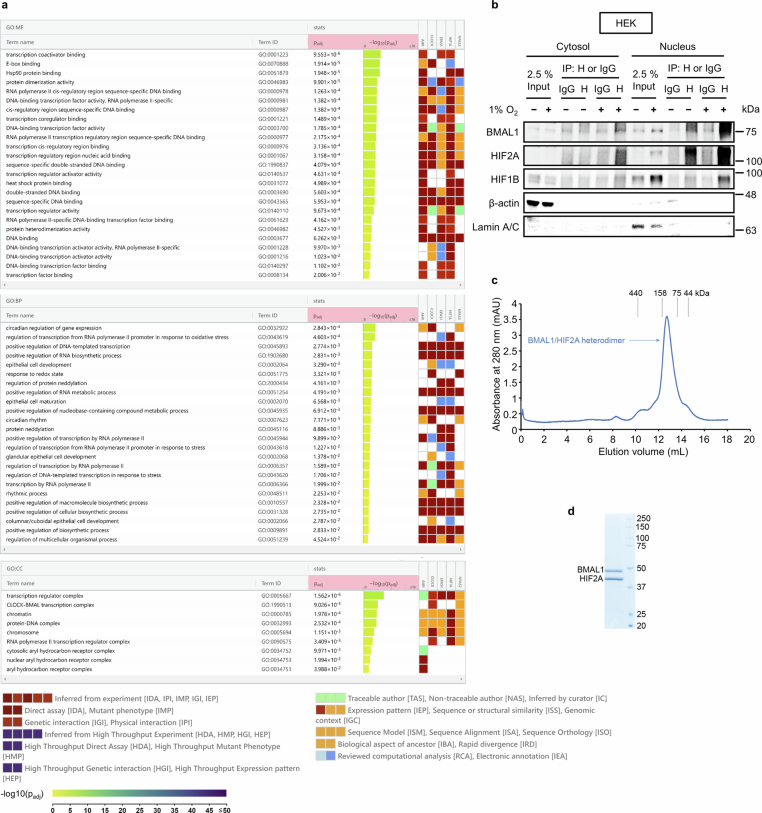
Interaction of BMAL1 and HIF2A. **a**, GO enrichment analysis was conducted to elucidate the molecular functions (MF), biological processes (BP), and cellular components (CC) associated with proteins predicted by the HuRI to potentially interact with BMAL1; two-sided Fisher’s exact test, Benjamini–Hochberg correction applied. **b**, Western Blot analysis of reciprocal co-IP with HIF2A in hypoxia-treated (1% O_2_, 4 h) or normoxia-treated HEK293 cells. Cytosolic and nuclear protein extracts were immunoprecipitated with HIF2A and blotted with anti-BMAL1, anti-HIF2A, anti-HIF1B, anti-β-actin, and anti-Lamin A/C antibodies. An IgG control affirmed procedure specificity. H indicates HIF2A. n = 3 independent experiments. **c**, Size-exclusion chromatography analysis of the BMAL1/HIF2A heterodimer. The purified BMAL1/HIF2A complex was loaded onto a Superdex 200 Increase 10/300 GL column. n = 3 independent experiments. The molecular weights of makers are as indicated. **d**, SDS-PAGE analysis of the recombinant BMAL1/HIF2A heterodimer purified by size-exclusion chromatography. n = 3 independent experiments.

As a key member of the basic helix-loop-helix PER–ARNT–SIM (bHLH-PAS) family, BMAL1 exerts regulatory functions through interactions with specific partners^8,10,11,20^. Using the Human Reference Interactome (HuRI)^36^, we identified hypoxia-inducible factor 2 alpha (HIF2A, also known as EPAS1), another bHLH-PAS family member, as the most abundantly expressed BMAL1 interactor in human LV (largest node in Fig. 2a). GO analysis highlighted shared roles of BMAL1 and HIF2A in transcriptional regulation and cellular responses to hypoxia and oxidative stress (Extended Data Fig. 2a and Supplementary Table 8). Given the severe hypoxia during MI^13,15,26,28^ and HIF2A’s established role in oxygen homeostasis^16,37^, we examined the BMAL1–HIF2A interaction under hypoxic conditions. Co-immunoprecipitation (co-IP) assays in HEK293 cells revealed a robust BMAL1–HIF2A nuclear interaction under ambient hypoxia (1% O_2_, 4 h), distinct from the oxygen-insensitive BMAL1–CLOCK interaction (Fig. 2b,c and Extended Data Fig. 2b). Consistent with previous reports^38^, binding of BMAL1 to HIF1A is considerably weaker (Fig. 2b,c). We further validated in primary human cardiomyocytes (HCMs) using endogenous co-IP and proximity ligation assay (PLA)^39^, confirming a robust BMAL1–HIF2A interaction at the single-molecule level under hypoxia, while BMAL1–HIF1A binding was also detected, albeit at significantly lower levels (Fig. 2d–f). The N-terminal regions of BMAL1 and HIF2A, containing conserved bHLH and PAS-A/B domains, mediate partner dimerization^8,40^ (Fig. 2g), while their C-terminal transactivation domains enable transcriptional activation^40^. Pull-down assays using recombinant BMAL1 and HIF2A demonstrated direct binding between their N-terminal regions (Fig. 2h). Size-exclusion chromatography confirmed the formation of a stable BMAL1–HIF2A heterodimer (Extended Data Fig. 2c,d). Despite a 67% sequence identity between the N-terminal regions of HIF1A and HIF2A, BMAL1 exhibits notably weaker binding to HIF1A (Fig. 2h). These results demonstrate a specific and direct interaction between BMAL1 and HIF2A during hypoxia.

## HIF2A regulates circadian cardiac injury

**Extended Data Fig. 3:**
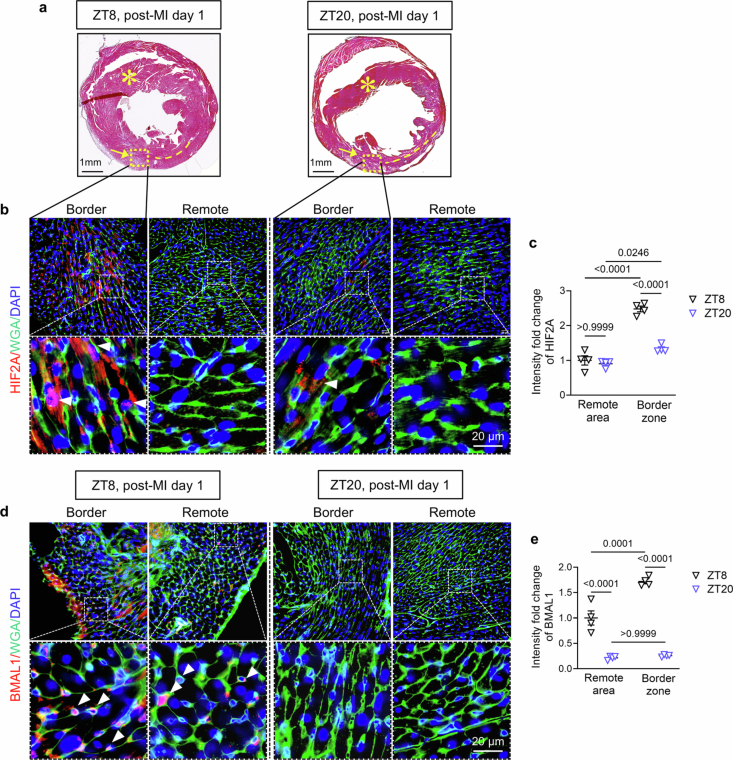
Increased expression of BMAL1 and HIF2A in the border zone at ZT8. **a**, Representative trichrome staining of heart sections on day 1 post‐MI. Border and remote areas are identified with an arrow and a star, respectively. The dashed lines highlight the extension of the areas of ischaemic injury, scale bar, 1 mm. **b** and **d**, Representative immunostaining of HIF2A (red) **(b)**, BMAL1 (red) **(d)**, WGA (green), and nuclei (DAPI; blue) in the border zone and remote area of hearts from C57BL/6J mice subjected to myocardial IRI at ZT8 or ZT20, n = 4 mice/group/time point, scale bar, 20 μm. White arrows indicate HIF2A or BMAL1 within the nuclei and cytoplasm. **c** and **e**, Quantification of fluorescence intensity of HIF2A in **(b)** and BMAL1 in **(d)**. Normalized to the protein levels in the remote areas at ZT8. Each quantification value dot represents the average value of three fields in one section. n = same as above. Data are mean ± s.e.m. Statistical analysis was performed using two-way ANOVA. Source Data

Although HIF2A is well recognized for its cardioprotective role against myocardial IRI^16,28,41^, its involvement in diurnal cardiac injury remains unclear. After 2 h of reperfusion, HIF2A protein levels were notably stabilized in the nuclear fraction of the AAR at ZT8 but not ZT20, paralleling BMAL1 expression (Fig. 2i,j), suggesting a time-of-day-dependent myocardial hypoxic response. On day 1 after MI, immunofluorescence revealed significantly higher HIF2A levels in the cytoplasm and nuclei of cardiomyocytes in the border zone (ischaemic region) at ZT8 compared with at ZT20, while expression remained low in the remote myocardium (Extended Data Fig. 3a–c). BMAL1 displayed a similar diurnal nuclear localization pattern (Extended Data Fig. 3d,e), with significantly greater co-localization with HIF2A in the ZT8 border zone (Fig. 2k), indicating circadian regulation of their interaction. To elucidate the functional role of HIF2A in circadian myocardial injury, we used an inducible cardiomyocyte-specific *Hif2a* knockout model (*Hif2a*^*loxP/loxP*^ myosin-Cre^+^)^16,41^. Similar to the *Bmal1*-deficient mice, these mice exhibited abolished circadian-dependent cardiac injury and diminished endogenous cardioprotection at ZT8 (Fig. 2l–o). On day 14 after MI, STE revealed worsened systolic function, increased LV dilation and mass, and greater wall motion abnormalities and intraventricular disparities in ZT8 *Hif2a*^*loxP/loxP*^ myosin-Cre^+^ mice compared with the controls (Fig. 2p–s and Supplementary Fig. 2d–f). These changes were absent at ZT20. By contrast, cardiomyocyte *Hif1a* deletion did not affect circadian variations in myocardial injury (Supplementary Fig. 3a–k). These results highlight a selective role for cardiomyocyte-specific HIF2A in regulating diurnal susceptibility to IRI.

## BMAL1–HIF2A drives diurnal *AREG*

**Fig. 3:**
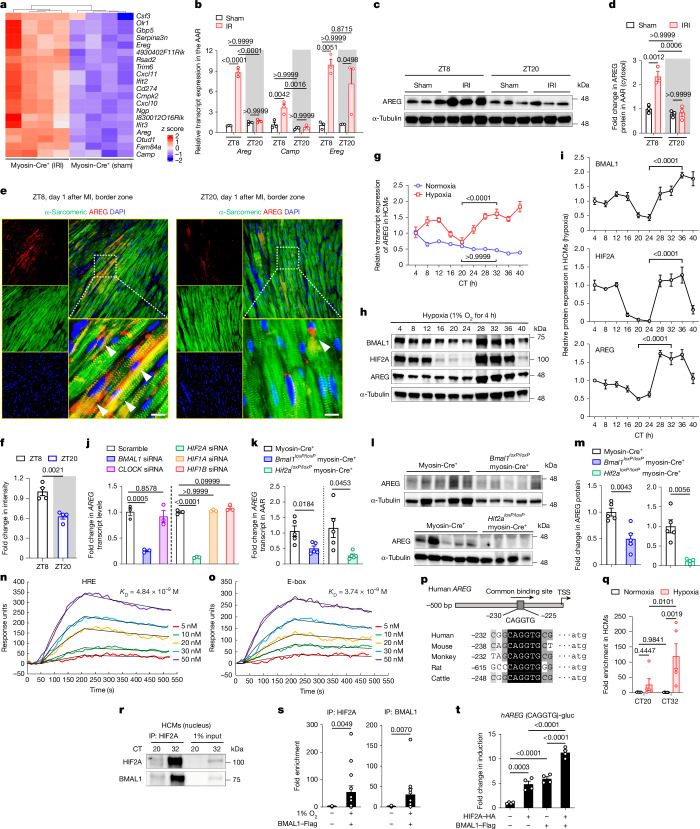
*Areg* is a circadian-dependent target of the BMAL1–HIF2A heterodimer. **a**, Heat map of the top 20 potential HIF2A targets. *n* = 4 mice per group. **b**–**d**, HIF2A targets (**b**) and AREG protein (western blot (**c**) and quantification (**d**)) in the AAR of C57BL/6J mice after 2 h reperfusion. *n* = 3 mice per group per timepoint. Owing to similar molecular masses of proteins, the samples were run on separate gels, with α-tubulin as the sample processing control. **e**,**f**, AREG immunostaining in the border zone on day 1 after MI (**e**; scale bars, 25 μm) and quantification (**f**). Arrows indicate α-sarcomeric^+^/AREG^+^ cells. *n* = 4 mice per timepoint. **g**–**i**, Synchronized HCMs were exposed to hypoxia (1% O_2_, 4 h) across circadian times (CT0–CT40); *AREG* mRNA expression (**g**) and BMAL1–HIF2A/AREG protein (**h**) and quantification (**i**) are shown. *n* = 3. **j**, Analysis of *AREG* mRNA in HEK293 cells transfected with various siRNAs and exposed to hypoxia. *n* = 3. **k**–**m**, The fold change in *Areg* mRNA transcripts (**k**), and western blot (**l**) and quantification (**m**) of cardiomyocyte AREG after 2 h reperfusion in the AAR of myosin-Cre^+^, *Bmal1*^*loxP/loxP*^ myosin-Cre^+^ and *Hif2a*^*loxP/loxP*^ myosin-Cre^+^ mice. *n* = 5 (transcripts) mice per group; and *n* = 5, 5 and 4 (proteins) mice. Statistical analysis was performed using unpaired two-tailed *t*-tests, with Welch’s *t*-tests comparing between myosin-Cre^+^ and *Hif2a*^*loxP/loxP*^ myosin-Cre^+^ mice (protein). **n**,**o**, Surface plasmon resonance analysis of BMAL1–HIF2A binding to HRE (**n**) or E-box (**o**). *n* = 3. **p**, Conserved BMAL1–HIF2A-binding site (CAGGTG) on the *AREG* promoter. **q**, ChIP–qPCR analysis of HIF2A binding in HEK293 cells at this shared site at CT20 or CT32. *n* = 5. **r**, Co-IP analysis of HIF2A–BMAL1 interactions in HCMs at CT20/CT32. *n* = 3. **s**, ChIP–qPCR analysis of HEK293 cells for *HIF2A* (*n* = 13) and *BMAL1* (n = 12 (normoxia) and *n* = 11 (hypoxia)). **t**, Luciferase assays of *hAREG* promoter activation in HEK293 cells. *n* = 4. All of the samples are biologically independent. Data are mean ± s.e.m. Statistical analysis was performed using one-way ANOVA (**g**,**i**,**j** and **t**), two-way ANOVA (**b**,**d**,**q**), unpaired two-tailed *t*-tests (**f**) and two‐sided Mann–Whitney *U*-test (**s**). Source Data

**Extended Data Fig. 4:**
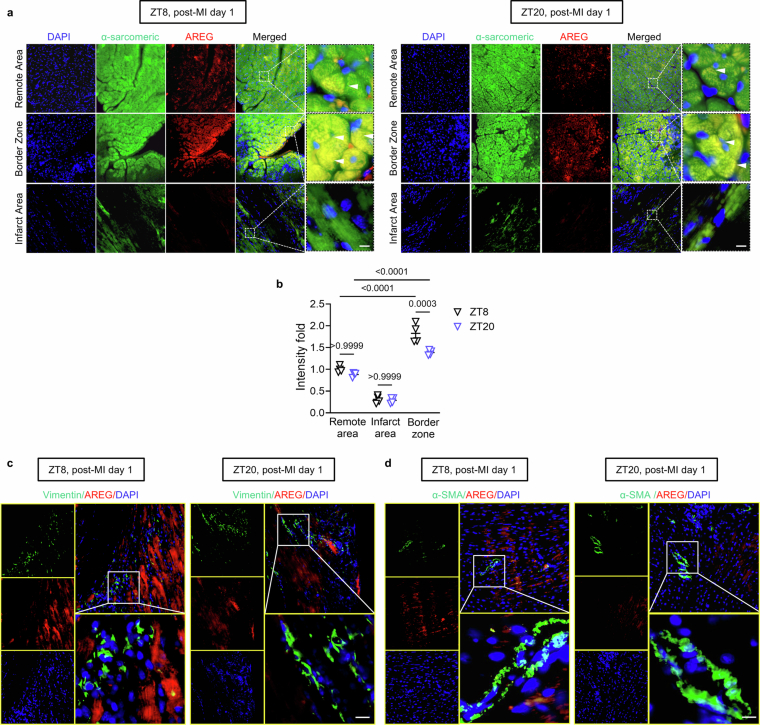
Increased induction of AREG in cardiomyocytes in the border zone at ZT8. **a-b, a**, Representative immunostaining of AREG (red), α-sarcomeric (cardiomyocyte marker; green), and nuclei (DAPI; blue) on day 1 post-MI in the border zone, infarct area, and remote area of hearts from C57BL/6J mice subjected to myocardial IRI at ZT8 or ZT20, scale bar, 25 μm. Arrows indicate α-sarcomeric^+^/AREG^+^ cells. **b**, Quantification of fluorescence intensity of AREG in (**a**). Normalized to the AREG levels in the remote areas at ZT8. Each quantification value dot represents the average value of three fields in one section. n = 4 mice/group/time point. Data are mean ± s.e.m. Statistical analysis was performed using two-way ANOVA. **c-d**, Representative immunostaining of AREG (red), vimentin (fibroblast marker; green) (**c**), α-smooth muscle actin (α-SMA, smooth muscle cell marker; green) (**d**) and nuclei (DAPI; blue) on day 1 post-MI in the border zone of hearts from C57BL/6J mice subjected to myocardial IRI at ZT8 or ZT20, scale bar, 25 μm. n = 4 mice/group/time point. Source Data

Next, to investigate how the BMAL1–HIF2A complex influences diurnal variations of myocardial injury, we reanalysed our previously published microarray data from *Hif2a*^*loxP/loxP*^ myosin-Cre^+^ and myosin-Cre^+^ mice^16^. Among potential HIF2A target genes that are uniquely upregulated in myosin-Cre^+^ mice after IRI (Fig. 3a and Supplementary Table 9), *Areg*—a known HIF2A target and member of the epidermal growth factor family^16,17^—exhibited the largest diurnal fold change in the AAR at ZT8 compared with at ZT20 (Fig. 3b and Supplementary Table 10). AREG protein levels in cytosolic extracts mirrored this diurnal pattern, peaking at ZT8 (Fig. 3c,d). Immunofluorescence staining confirmed elevated AREG expression in the cytoplasm of cardiomyocytes within the border zone at ZT8, while infarcted and remote myocardium showed low levels (Fig. 3e,f and Extended Data Fig. 4a,b). Minimal AREG expression was detected in fibroblasts or smooth muscle cells (Extended Data Fig. 4c,d). This spatiotemporal pattern aligns with BMAL1 and HIF2A nuclear co-localization in the ZT8 border zone (Fig. 2k), suggesting that the BMAL1–HIF2A complex drives diurnal *Areg* induction in ischaemic hearts.

To assess the role of the cell-intrinsic molecular clock in *AREG* regulation, synchronized HCMs were exposed to hypoxia (1% O_2_, 4 h) at different circadian times (CT0 to CT40). CT0 marks the start of the circadian cycle in vitro, corresponding to ZT0, the onset of the light phase in light-entrained systems. Under normoxia, *AREG* transcript levels were non-rhythmic, but hypoxia significantly induced *AREG* at CT32 (analogous to ZT8), with a much weaker induction at CT20 (Fig. 3g). This pattern mirrored the rhythmic expression of the BMAL1–HIF2A complex in HCMs (Fig. 3h,i), suggesting circadian-dependent, hypoxia-driven regulation of *AREG*. Silencing *HIF2A* or *BMAL1* with small interfering RNA (siRNA) significantly reduced hypoxia-induced *AREG* expression, whereas *CLOCK*, *HIF1A* or *HIF1B* knockdown had no effect (Fig. 3j). Consistently, myocardial IRI-induced *Areg* expression was markedly reduced in cardiomyocytes from *Bmal1*- or *Hif2a-*deficient mice (Fig. 3k–m). Together, these findings underscore the BMAL1–HIF2A complex as a key regulator of rhythmic *AREG* induction during both hypoxia and myocardial IRI.

## BMAL1–HIF2A synergistically activates *AREG*

To examine how the BMAL1–HIF2A heterodimer regulates *AREG*, we investigated its DNA-binding properties and potential transcriptional synergy under hypoxia. HIF2A–HIF1B and BMAL1–CLOCK complexes bind to hypoxia response elements (HREs, [A/G]CGTG)^40^ and E-box motifs (CACGTG)^8^, respectively, which share notable sequence similarity. Electrophoretic mobility shift assay (Supplementary Fig. 3l) and surface plasmon resonance analysis (Fig. 3n,o) demonstrated robust and comparable binding of the BMAL1–HIF2A heterodimer to both motifs, confirming its ability to recognize DNA elements of target genes. Analysis of the human *AREG* promoter using JASPAR (https://jaspar.elixir.no/; v.2022) revealed a conserved binding site (CAGGTG) for BMAL1 and HIF2A across species (Fig. 3p). Chromatin immunoprecipitation followed by qPCR (ChIP–qPCR) in HCMs confirmed significant binding enrichment of endogenous HIF2A to this site under hypoxia at CT32 but not at CT20 (Fig. 3q), indicating circadian gating of HIF2A transcriptional activity. This finding aligns with circadian-dependent BMAL1–HIF2A interaction, which peaks at CT32 (Fig. 3r) and corresponds to enhanced *AREG* induction (Fig. 3g). Additional ChIP–qPCR confirmed direct BMAL1 binding to the same conserved site during hypoxia (Fig. 3s). To evaluate functional cross-talk between BMAL1 and HIF2A at the chromatin level, the conserved sequence was cloned into a luciferase reporter and transfected into HEK293 cells. Both proteins enhanced luciferase activity, with co-transfection producing a synergistic effect and greater activation (Fig. 3t). These findings demonstrate that BMAL1 and HIF2A function as an integrated regulatory unit, synergistically driving rhythmic *AREG* transcription during hypoxia.

## *Areg* knockdown abolishes circadian injury

**Fig. 4:**
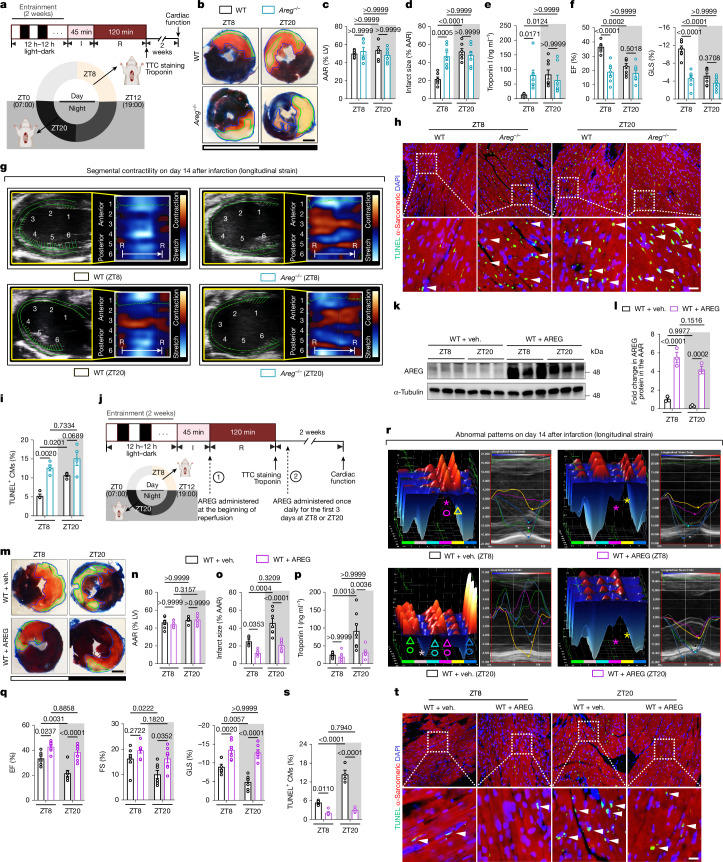
AREG drives circadian-dependent cardioprotection. **a**, Schematic of myocardial injury and cardiac function assessment in *Areg*^*−/−*^ mice and WT mice subjected to IRI at ZT8 or ZT20. **b**, Heart slices were stained with Evan’s blue and TTC after 2 h reperfusion. Scale bar, 1 mm. **c**, The AAR as a percentage of the LV. **d**, The infarct size as a percentage of the AAR. *n* = 7 mice per group per timepoint. **e**, Serum troponin I levels. *n* = 8 mice per group per timepoint. **f**,**g**, Cardiac function on day 14 after MI was determined using STE. **f**, The EF and GLS. **g**, *B*-mode images with 2D longitudinal strain. 1, anterior base; 2, anterior middle; 3, anterior apex; 4, posterior apex; 5, posterior middle; 6, posterior base. *n* = 8 mice per group per timepoint. **h**,**i**, TUNEL staining in the border zone on day 1 after MI (**h**; scale bar, 25 μm) and quantification (**i**). White arrows point out TUNEL-positive (green) cardiomyocyte (red) nuclei (blue). *n* = 4 mice per group per timepoint. **j**, Schematic of the cardiac injury and function assessment in AREG-treated or vehicle (veh.)-treated mice subjected to IRI at ZT8 or ZT20. Treatment was initiated at reperfusion and continued daily for 3 days. **k**,**l**, AREG protein in the AAR (**k**) with quantification (**l**). *n* = 3 mice per group per timepoint. **m**–**p**, Evan’s blue- and TTC-stained heart slices (**m**, scale bar, 1 mm), the AAR as a percentage of the LV (**n**), the infarct size as a percentage of the AAR (**o**) and serum troponin I levels (**p**) after 2 h reperfusion. *n* = 7 mice per group per timepoint. **q**,**r**, Cardiac function on day 14 after MI. **q**, The EF, FS and GLS. **r**, LV 3D images with six-segment longitudinal strain. *n* = 7 mice per group per timepoint. **s**,**t**, TUNEL staining in the border zone. The percentage of TUNEL-positive cells (**s**) and representative images (**t**) are shown. White arrows indicate TUNEL-positive (green) cardiomyocyte (red) nuclei (blue). Scale bar, 25 μm. *n* = 5 (veh., ZT8), *n* = 4 (veh., ZT20), *n* = 5 (AREG, ZT8) and *n* = 5 (AREG, ZT20). All data are mean ± s.e.m. For **c**–**f**,**i**,**l**,**n**–**q** and **s**, statistical analysis was performed using two‐way ANOVA. The diagrams in **a** and **j** were created using BioRender. Source Data

To determine whether AREG directly contributes to circadian variations in myocardial injury, we analysed its role using genetic deletion models. Similar to *Bmal1*- and *Hif2a-*deficient mice, *Areg*^*−/−*^ mice exhibited a loss of circadian variations in myocardial injury and diminished endogenous cardioprotection at ZT8 after 2 h of reperfusion (Fig. 4a–e). Diurnal variations in long-term outcomes, including cardiac function, LV size and mass, segmental wall motion abnormalities and intraventricular synchronicity, observed in wild-type (WT) controls, were completely absent in *Areg*^*−/−*^ mice by day 14 after MI (Fig. 4f,g and Supplementary Fig. 4a–d). Terminal deoxynucleotidyl-transferase-mediated dUTP-biotin nick-end labelling (TUNEL) staining, which detects double-stranded DNA breaks as a marker of apoptosis, revealed significantly increased cardiomyocyte apoptosis in the border zone of *Areg*^*−/−*^ mice at ZT8 compared with in WT mice, with minimal differences observed at ZT20 (Fig. 4h,i). These findings indicate a functional role of AREG in regulating daytime-dependent cardiac injury.

## AREG provides circadian cardioprotection

Building on AREG’s critical role in circadian injury modulation, we assessed its therapeutic potential through exogenous administration and gene delivery. To mimic a clinical scenario of reperfusion treatment, recombinant AREG (10 µg) or vehicle was administered to C57BL/6J mice at the onset of reperfusion. Timed AREG treatment elevated AREG protein levels in the AAR (Fig. 4j–l) and significantly reduced infarct size and serum troponin I levels after 2 h of reperfusion. Notably, protection was greater at ZT20, potentially compensating for the lower endogenous AREG levels at that time (Fig. 4m–p). Furthermore, AREG (10 µg) administered daily for 3 days after injury at ZT20 improved LV systolic function, reduced cardiac remodelling, restored segmental contractility and re-established normokinesis by day 14 after MI compared with the vehicle controls (Fig. 4q,r and Supplementary Fig. 4e–h). TUNEL staining on day 1 after MI revealed notably reduced cardiomyocyte apoptosis in the border zone in ZT20-treated mice (Fig. 4s,t). To further assess its therapeutic potential, we used adeno-associated virus serotype 9 (AAV9) to deliver the *Areg* gene under the muscle creatine kinase (MCK) promoter for cardiomyocyte-specific targeting. Tail-vein injection of Areg-AAV (2 × 10^11^ genome copies (GC) per mouse) significantly increased AREG levels in the heart by day 28, with minimal off-target expression (Supplementary Fig. 5a–e). This intervention reduced the infarct size and improved cardiac function, with greater effects at ZT20 (Supplementary Fig. 5f–n). These findings establish AREG as a promising therapeutic target for time-optimized interventions, offering strategies to harness circadian rhythms for improved cardiac protection.

## Targeting BMAL1 for cardioprotection

**Extended Data Fig. 5:**
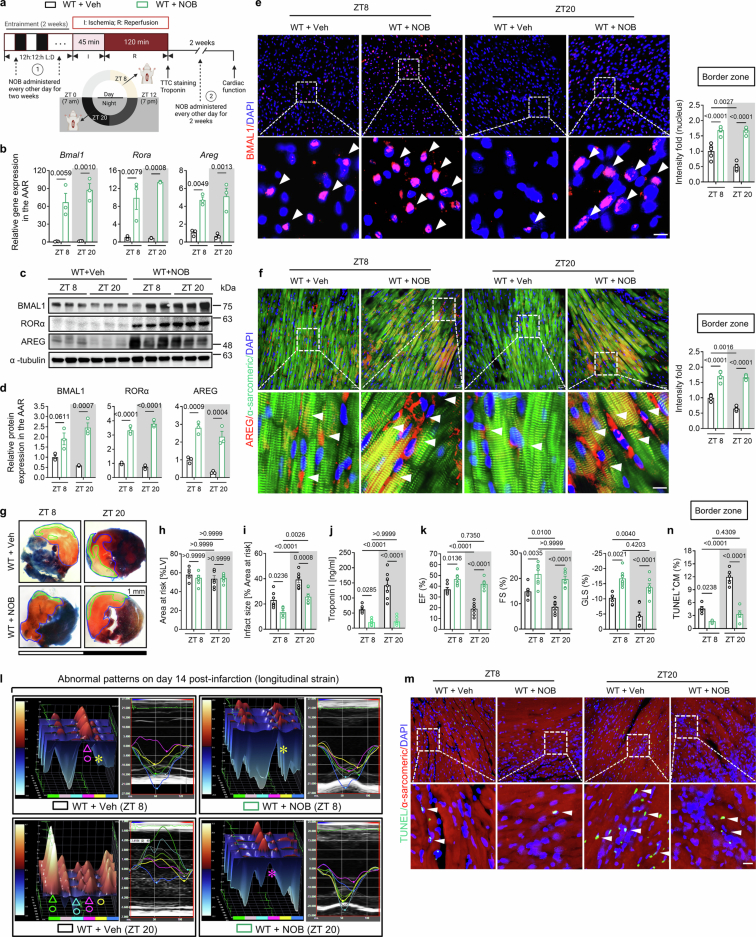
Targeting BMAL1 using NOB for circadian-dependent cardioprotection. **a**, Experimental setup for evaluating myocardial injury and cardiac function in NOB-treated (200 mg/kg, i.p., every other day) or Veh-treated C57BL/6J mice subjected to myocardial IRI at ZT8 or ZT20. **b-d**, BMAL1, RORα, and AREG transcript levels in the AAR after 2 h of reperfusion (**b**) and protein levels by Western blot (**c**) with quantification (**d**). Due to similar molecular weight of proteins, some samples were run on separate gels with equal loading volumes, using α-tubulin as sample processing control. n = 3 mice/group/time point; two-way ANOVA. **e**, BMAL1 immunofluorescence (red) in cell nuclei (DAPI: blue) in the border zone on day 1 post-IRI, with quantification. Scale bar, 25 μm. n = 5 mice/Veh-treated/ZT8, n = 4 mice/Veh-treated/ZT20, n = 5 mice/NOB-treated/ZT8, n = 5 mice/NOB-treated/ZT20; two-way ANOVA. **f**, AREG immunofluorescence (red), α-sarcomeric (green), and nuclei (DAPI: blue) in the border zone, with quantification. Scale bar, 25 μm. n = 4 mice/Veh-treated/ZT8, n = 5 mice/Veh-treated/ZT20, n = 4 mice/NOB-treated/ZT8, n = 5 mice/NOB-treated/ZT20; two-way ANOVA. **g–j**, Evan’s blue and TTC-stained heart slices (**g**), AAR as % LV (**h**), infarct size as % AAR (**i**), and serum troponin I levels (**j**) after 2 h of reperfusion. n = 7 mice/group/time point; two-way ANOVA. **k** and **l**, Cardiac function on day 14 post-MI assessed by STE. EF, FS, and GLS (**k**) and 3D longitudinal strain with six-segment images showing LV motion abnormalities (**l**). Colour-coded segments: Anterior Base (dark blue), Anterior Mid (yellow), Anterior Apex (magenta), Posterior Apex (cyan), Posterior Mid (light pink), and Posterior Base (green). Symbols indicate reduced contractility (stars), dyskinesis (triangles), and dyssynchrony (circles). n = 7 mice/group/time point; two-way ANOVA. **m** and **n,** TUNEL staining on day 1 post-MI (**m**) and quantification of TUNEL-positive cardiomyocytes (**n**); scale bar, 25 μm. n = 5 mice/group/time point; two-way ANOVA. All data are mean ± s.e.m. The diagram in **a** was created using BioRender. Source Data

To examine the translational potential of modulating circadian rhythms for cardioprotection, we used nobiletin (NOB), a flavonoid that enhances circadian rhythms by directly activating RORs^42^, key transcriptional activators of *Bmal1*^10,20^. NOB treatment in C57BL/6J mice (200 mg per kg, intraperitoneal (i.p.)) significantly upregulated RORα, leading to a 60–80-fold increase in *Bmal1* mRNA and a 2–3-fold increase in protein levels at both ZT8 and ZT20, ultimately inducing AREG upregulation in the heart (Extended Data Fig. 5a–d). Immunofluorescence analysis confirmed elevated nuclear BMAL1 and cytoplasmic AREG in cardiomyocytes in the border zone on day 1 after MI (Extended Data Fig. 5e,f). NOB treatment significantly attenuated acute myocardial injury, improved long-term cardiac function and reduced cardiomyocyte apoptosis, with more pronounced protection observed at ZT20 (Extended Data Fig. 5g–n), probably due to its ability to compensate for the natural trough in nuclear BMAL1 expression and transcriptional activity at this time^10,11,20^. To determine whether NOB’s cardioprotective effects depend on the BMAL1–HIF2A–AREG pathway, we evaluated its efficacy in genetic knockout models. Cardioprotection was completely abrogated in *Hif2a*-deficient mice treated with NOB, as evidenced by larger infarct sizes and significantly impaired cardiac function (Supplementary Fig. 6a–h). Similarly, NOB treatment failed in *Bmal1*^*loxP/loxP*^ myosin-Cre^+^ mice (Supplementary Fig. 6i–o) and *Areg*^*−/−*^ mice (Supplementary Fig. 6p–u), confirming the essential role of this pathway. To further assess the benefits of BMAL1 overexpression, we used AAV9 to deliver *Bmal1* specifically to cardiomyocytes (Supplementary Fig. 7a–e). BMAL1 overexpression significantly reduced infarct size and improved cardiac function with greater protection observed at ZT20 (Supplementary Fig. 7f–o). These effects mirrored those of NOB treatment, highlighting the therapeutic potential of BMAL1 activation in attenuating myocardial injury and advancing the development of circadian-targeted pharmacological strategies.

## Activating HIF2A for circadian protection

**Extended Data Fig. 6:**
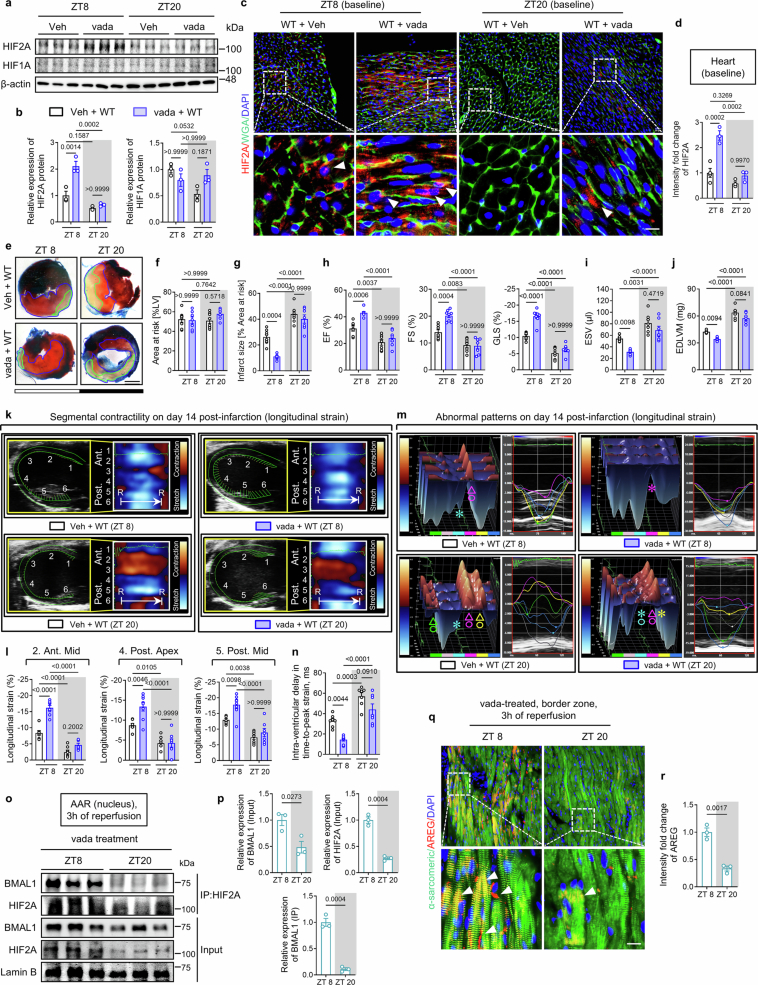
Stabilization of HIF2A by vadadustat treatment provides circadian-dependent cardioprotection. **a-d**, C57BL/6J mice treated with vadadustat (vada, 50 mg/kg, i.p., daily) or Vehicle (Veh) for three days at ZT8 or ZT20. HIF2A and HIF1A protein levels assessed by Western blot (**a**) and quantified (**b**). n = 3 mice/group/time point; two-way ANOVA. Immunostaining of HIF2A (red), WGA (green), and nuclei (DAPI, blue) in mouse hearts (**c**; scale bar: 25 μm), with quantification of HIF2A fluorescence intensity (**d**). n = 4 mice/Veh-treated/ZT8, n = 4 mice/Veh-treated/ZT20, n = 3 mice/vada-treated/ZT8, and n = 3 mice/vada-treated/ZT20; two-way ANOVA. **e-g**, Mice given an additional dose of vadadustat 2 h before IRI at ZT8 or ZT20. Evan’s blue and TTC-stained heart slices (**e**), quantification of AAR as % LV (**f**), and infarct size as % AAR (**g**) after 2 h of reperfusion. n = 8 Veh-treated/ZT8, n = 8 Veh-treated/ZT20, n = 7 vada-treated/ZT8, and n = 7 vada-treated/ZT20; two-way ANOVA. **h-n**, Cardiac function on day 14 post-MI assessed by STE. LV systolic function (EF, FS, GLS; **h**), ESV (**i**), EDLVM (**j**), B-mode imaging with 2D longitudinal strain (**k**), segmental wall contractility (**l**), 3D longitudinal strain with images showing LV motion abnormalities (**m**), and mechanical dyssynchrony measured by intra-ventricular delay (**n**). Symbols in (**m**) indicate reduced contractility (stars), dyskinesis (triangles), and dyssynchrony (circles). n = 7 mice/Veh-treated/ZT8, n = 7 mice/Veh-treated/ZT20, n = 8 mice/vada-treated/ZT8, and n = 7 mice/vada-treated/ZT20; two-way ANOVA. **o** and **p**, Mice treated with vadadustat for three days at ZT8 or ZT20, followed by IRI. Nuclear fractions from the AAR 3 h post-reperfusion were immunoprecipitated with HIF2A and blotted for HIF2A, BMAL1, and Lamin B (**o**) with quantification of BMAL1 and HIF2A protein levels (**p**). n = 3 mice/group; unpaired two-tailed *t*-tests. **q** and **r**, Immunostaining of AREG (red), α-sarcomeric (green), and nuclei (DAPI: blue) 3 h post-reperfusion (**q**, scale bar, 25 μm), with quantification of fluorescence intensity (**r**). n = 3 mice/group; unpaired two-tailed *t*-tests. All data are mean ± s.e.m. Source Data

To investigate the therapeutic potential of HIF2A activation in myocardial injury, C57BL/6J mice were treated with vadadustat, a prolyl hydroxylase domain (PHD) inhibitor^15^ (50 mg per kg, i.p., daily for 3 days) at ZT8 or ZT20. Vadadustat selectively stabilized HIF2A, particularly at ZT8, without affecting HIF1A levels (Extended Data Fig. 6a–d), potentially due to circadian regulation of PHD enzyme activity^31^. This stabilization significantly reduced infarct sizes and improved cardiac function in ZT8-treated mice, indicating time-dependent cardioprotection (Extended Data Fig. 6e–n). Analysis using co-IP revealed enhanced BMAL1–HIF2A binding in the nuclear fraction of the AAR at ZT8, accompanied by increased AREG expression in cardiomyocytes in the border zone after 3 h of reperfusion (Extended Data Fig. 6o–r). These findings suggest that BMAL1–HIF2A interaction and synergistic AREG induction underlie the circadian-dependent cardioprotection conferred by vadadustat at ZT8. To confirm this mechanism, vadadustat treatment was evaluated in *Bmal1*-deficient mice, which showed abolished cardioprotection at ZT8, highlighting a BMAL1-dependent mechanism (Supplementary Fig. 8a–g). A similar loss of protection was observed in *Hif2a*- and *Areg-*deficient mice, further supporting the essential role of the BMAL1–HIF2A–AREG pathway (Supplementary Fig. 8h–s). Moreover, cardiomyocyte-specific human-*HIF2A* knock-in mice (*LSL-HIF2dPA* myosin-Cre^+^) exhibited similar circadian-dependent HIF2A stabilization and increased *Areg* induction at ZT8 in mouse hearts (Supplementary Fig. 9a–c). Consistent with vadadustat treatment, HIF2A overexpression significantly reduced infarct size and improved cardiac function, with the most pronounced benefits at ZT8 (Supplementary Fig. 9d–m). These findings highlight the therapeutic potential of time-of-day-dependent HIF2A stabilization in enhancing cardioprotection, paving the way for new chronotherapy interventions.

## BMAL1 stabilizes HIF2A protein

**Extended Data Fig. 7:**
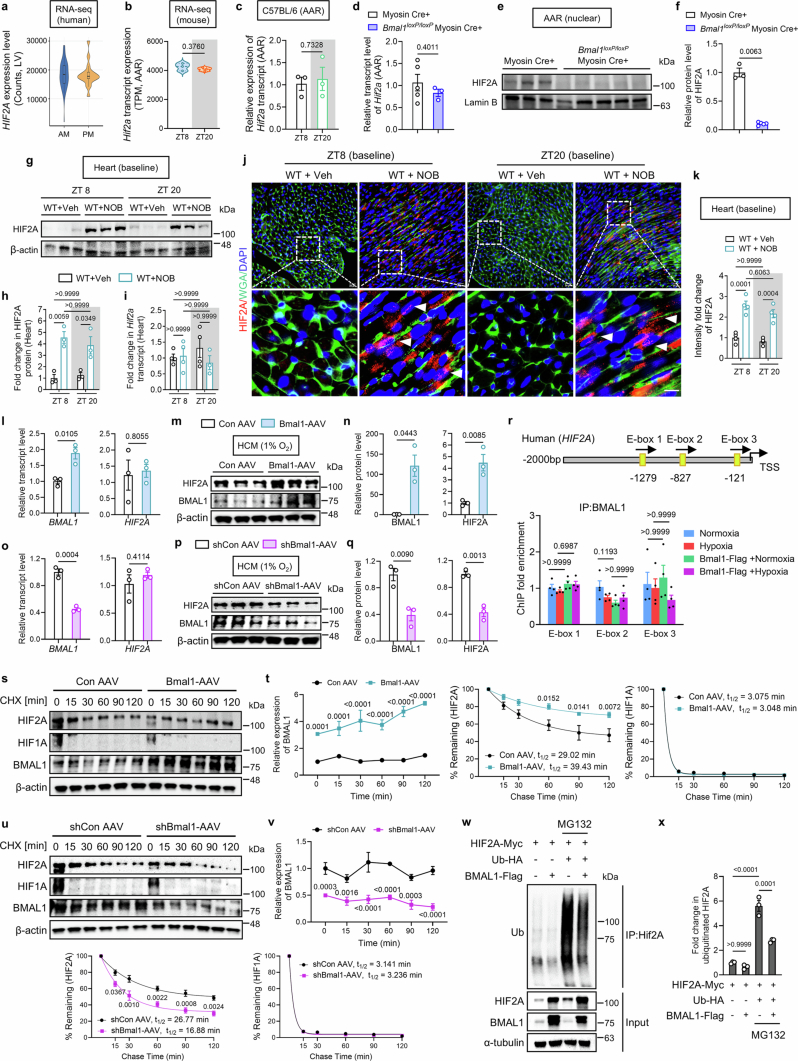
BMAL1 stabilizes HIF2A protein. **a** and **b**, *HIF2A* transcript levels from RNA-seq of LV biopsy samples after aortic cross-clamping in morning and afternoon cardiac surgery patients (**a**, n = 56 morning, n = 17 afternoon; boxplots show the 25th and 75th percentiles (box), median (central line), and minimum to maximum values (whiskers)) and in the AAR of mice after 2 h of reperfusion at ZT8 or ZT20 (**b**, n = 3 mice/time point; unpaired two-tailed *t*-tests). **c**, *Hif2a* transcript levels by real-time PCR after 2 h of reperfusion in the AAR subjected to IRI at ZT8 or ZT20. n = 3 mice/time point; unpaired two-tailed *t*-tests. **d-f**, *Hif2a* transcript levels by real-time PCR in the AAR after 2 h of reperfusion in *Bmal1*^*loxP/loxP*^ Myosin Cre+ mice and Myosin Cre+ mice (**d**, n = 5/Myosin Cre+ and n = 3/*Bmal1*^*loxP/loxP*^ Myosin Cre + ; unpaired two-tailed *t*-tests). HIF2A protein levels by Western blot in the nuclear fractions (**e**, n = 3/Myosin Cre+ and n = 5/*Bmal1*^*loxP/loxP*^ Myosin Cre + ) and quantification (**f**, Welch’s *t*-tests). **g-k**, C57BL/6J mice treated with NOB (200 mg/kg, i.p.) and HIF2A protein levels were assessed by Western blot (**g, h**, n = 3 mice/group/time point; two-way ANOVA) or transcript levels by real-time PCR (**i**, n = 4 mice/Veh-treated/ZT8, n = 4 mice/Veh-treated/ZT20, n = 4 mice/NOB-treated/ZT8, and n = 3 mice/NOB-treated/ZT20; two-way ANOVA). Immunostaining of HIF2A in the myocardium (**j**, n = 4 mice/group/time point, scale bar, 25 μm) and quantification (**k**, two-way ANOVA). **l**-**q**, HCMs transduced with Bmal1-AAV or shBmal1-AAV were treated with 1% O_2_ for 4 h. *BMAL1* and *HIF2A* transcript levels by real-time PCR (**l, o**), protein levels by Western blot (**m, p**), and quantification (**n, q**). n = 3 independent experiments; Unpaired two-tailed *t*-tests were used for all comparisons, except for BMAL1 protein levels in (**n**), which were analysed using Welch’s *t*-tests. The samples used in **(m**) were also used in Extended Data Fig. 8j, sharing the β-actin blot. The samples used in (**p**) were also used in Extended Data Fig. 8m, sharing the β-actin blot. **r**, ChIP-qPCR of BMAL1 binding to E-box elements in the human *HIF2A* promoter under normoxic and hypoxic conditions. n = 4 independent experiments; one-way ANOVA. **s**-**v**, HCMs transduced with Bmal1-AAV (**s, t**) or shBmal1-AAV (**u, v**) were exposed to 1% O_2_ for 4 h and treated with CHX to assess HIF2A degradation. Protein levels were quantified and plotted to determine half-life using exponential decay. n = 3 independent experiments. **w, x**, HEK293 cells transfected with HIF2A-Myc, Ub-HA, and BMAL1-Flag, treated with MG132, and subjected to HIF2A immunoprecipitation. Ubiquitination levels were analysed (**w**) and quantified (**x**). n = 3 independent experiments; one-way ANOVA. All data are mean ± s.e.m. Source Data

Circadian-dependent HIF2A stabilization was observed after myocardial IRI, hypoxia, vadadustat administration and in cardiomyocyte-specific *HIF2A* knock-in mice (Figs. 2i,j, 3h,i, Extended Data Fig. 6a–d and Supplementary Fig. 9a,b), suggesting that clock genes may regulate HIF2A expression. However, RNA-seq and qPCR analyses revealed no circadian variations in *Hif2a* transcripts after IRI in humans and mice (Extended Data Fig. 7a–c). In *Bmal1*-deficient mice, nuclear HIF2A protein levels were reduced without changes in *Hif2a* transcripts, while NOB treatment further stabilized HIF2A protein at both ZT8 and ZT20, again without affecting transcript levels (Extended Data Fig. 7d–k). Gain-of-function and loss-of-function experiments in HCMs corroborated these findings, indicating a post-translational regulatory mechanism (Extended Data Fig. 7l–q). ChIP–qPCR analysis of predicted E-box elements in the *HIF2A* promoter showed no detectable BMAL1 binding, suggesting that BMAL1 does not directly regulate *HIF2A* transcriptionally (Extended Data Fig. 7r). Instead, cycloheximide (CHX) chase assays demonstrated that BMAL1 prolongs the HIF2A protein half-life by reducing its ubiquitin-mediated degradation (Extended Data Fig. 7s–x). Notably, BMAL1 did not affect HIF1A protein stability. These findings identify a role for BMAL1 in stabilizing HIF2A through post-translational mechanisms, offering insights into how circadian rhythms modulate hypoxic response during myocardial injury.

## BMAL1 does not disrupt HIF2A–HIF1B

**Extended Data Fig. 8:**
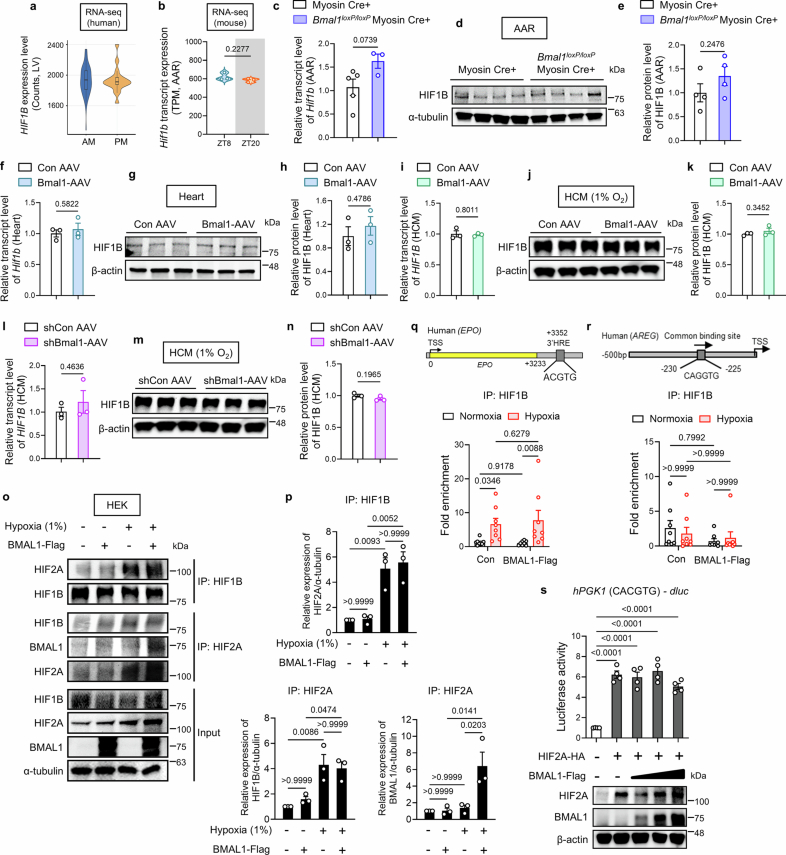
BMAL1 does not interfere with HIF2A/HIF1B interaction or its transcriptional activity. **a** and **b**, RNA-seq analysis of *HIF1B* transcript levels in LV biopsies after aortic cross-clamping in morning vs. afternoon patients (**a**, n = 56 AM, n = 17 PM; boxplots show the 25th and 75th percentiles (box), median (central line), and minimum to maximum values (whiskers)) and in the AAR after 2 h of reperfusion at ZT8 or ZT20 in mice (**b**, n = 3 mice/time point; unpaired two-tailed *t*-tests). **c-e**, *Hif1b* transcript levels in the AAR on day 1 post-IRI in *Bmal1*^*loxP/loxP*^ Myosin Cre+ and Myosin Cre+ mice (**c**, n = 5 Myosin Cre+ and n = 3 *Bmal1*^*loxP/loxP*^ Myosin Cre+ mice; unpaired two-tailed *t*-tests), HIF1B protein levels by Western blot (**d**, n = 4 mice/group) and quantification (**e**, unpaired two-tailed *t*-tests). **f-h**, *Hif1b* transcript levels in mouse hearts 28 days post-Bmal1-AAV or control AAV injection (**f**, n = 3 mice/group; unpaired two-tailed *t*-tests), HIF1B protein levels by Western blot (**g**, n = 3 mice/group) and quantification (**h**, unpaired two-tailed *t*-tests). The same samples in (**g**) were also used in Supplementary Fig. 7c, sharing the β-actin blot. **i-k**, *HIF1B* transcript levels in HCMs transduced with Bmal1-AAV or control AAV under hypoxia (**i**, n = 3 independent experiments; unpaired two-tailed *t*-tests), HIF1B protein levels by Western blot (**j**, n = 3 independent experiments), and quantification (**k**; unpaired two-tailed *t*-tests). The same samples used in (**j**) were also used in Extended Data Fig. 7m, sharing the β-actin blot. **l-n**, *HIF1B* transcript levels in HCMs transduced with shBmal1-AAV or shControl AAV (**l**, n = 3 independent experiments; unpaired two-tailed *t*-tests), HIF1B protein levels by Western blot (**m**, n = 3 independent experiments), and protein quantification (**n**; unpaired two-tailed *t*-tests). The same samples in (**m**) were also used in Extended Data Fig. 7p, sharing the β-actin blot. **o** and **p**, Immunoprecipitation using HIF1B and HIF2A antibodies in HEK293 cells transfected with BMAL1-Flag or control plasmid under hypoxia or normoxia, analysed by Western blot (**o**, n = 3 independent experiments) and normalized to α-tubulin (**p**, one-way ANOVA). **q** and **r**, ChIP-qPCR using HIF1B antibody showing binding to the human *EPO* (**q**) and *AREG* (**r**) promoters under normoxia and hypoxia. n = 8 independent experiments; two-way ANOVA. **s**, Luciferase assays in HEK293 cells transfected with BMAL1-Flag (0–200 nM), HIF2A-HA, and a luciferase reporter for the human *PGK1* promoter. n = 4 independent experiments; one-way ANOVA. All data are mean ± s.e.m. Source Data

Given BMAL1’s interaction with HIF2A, we assessed its influence on the HIF2A–HIF1B complex, which mediates canonical hypoxia signalling. RNA-seq analysis of ischaemic LV biopsy samples from patients and mouse AAR revealed no circadian variation in *Hif1b* transcript levels (Extended Data Fig. 8a,b). Gain-of-function and loss-of-function experiments in mice and HCMs further confirmed that BMAL1 does not regulate HIF1B transcript or protein levels during IRI or hypoxia (Extended Data Fig. 8c–n). Co-IP analysis showed that BMAL1 overexpression has no impact on HIF2A–HIF1B binding, while ChIP–qPCR revealed no changes in HIF1B binding to the erythropoietin (*EPO*) promoter^43^ (Extended Data Fig. 8o–q). Moreover, HIF1B was not observed to bind the *AREG* promoter, and luciferase reporter assays confirmed that BMAL1 does not affect the transcriptional activity of the HIF2A–HIF1B complex on *PGK1* (Extended Data Fig. 8r,s). These findings demonstrate that BMAL1 interacts with HIF2A without disrupting the canonical HIF2A–HIF1B complex, emphasizing its distinct role in circadian regulation of hypoxic responses.

## The cryo-EM structure of BMAL1–HIF2A–DNA

**Fig. 5:**
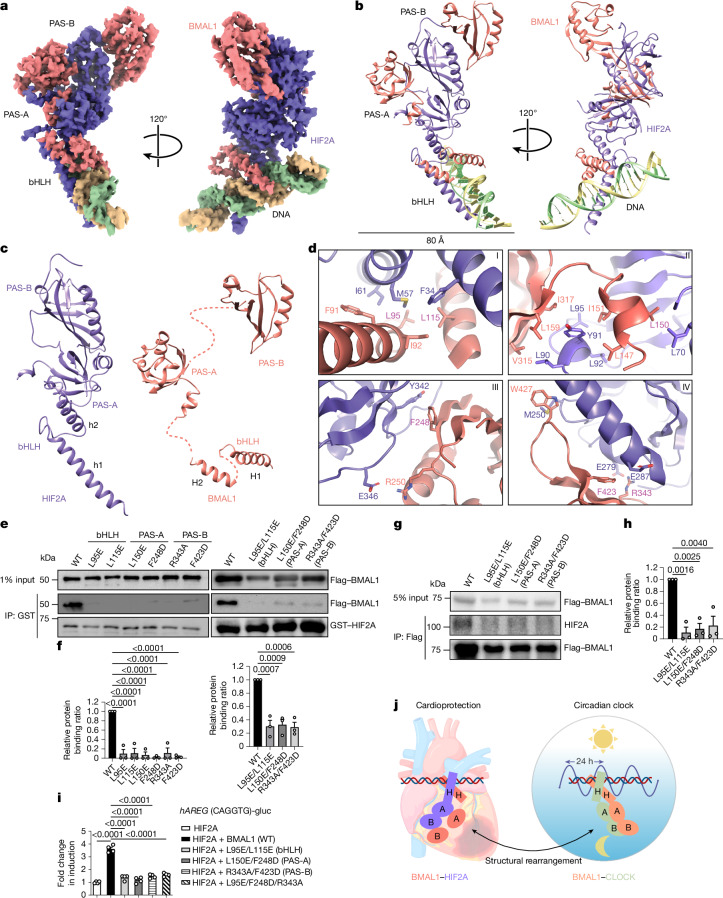
Structural analysis of the BMAL1–HIF2A heterodimer in a complex with DNA. **a**, Cryo-EM map of the BMAL1–HIF2A–DNA complex. The map was sharpened using DeepEMhancer. HIF2A and BMAL1 are coloured in purple and red, respectively, with two HRE DNA strands in green and yellow. **b**, The overall structure of the BMAL1–HIF2A–DNA complex. **c**, Individual structures of HIF2A and BMAL1 within the complex. **d**, The four major interfaces (I to IV) between HIF2A and BMAL1. Interaction residues in BMAL1 (red) and HIF2A (purple) are shown; the BMAL1 residues mutated for pull-down analysis in **e** are shown in magenta. **e**, Pull-down analysis showing impaired interaction between GST–HIF2A and Flag-tagged BMAL1 mutants. Mutations in the bHLH, PAS-A and PAS-B domains of the BMAL1 are indicated. *n* = 3 independent experiments. **f**, The relative binding of BMAL1 mutants compared with WT BMAL1, with WT BMAL1 binding to HIF2A set to 1. *n* = 3 independent experiments. **g**, HEK293 cells overexpressing WT or mutated Flag-tagged BMAL1 were exposed to ambient hypoxia (1% O_2_) for 4 h, followed by IP with Flag and blotted with the indicated antibodies. *n* = 3 independent experiments. **h**, Quantification of the relative binding in **g**. *n* = 3 independent experiments. **i**, HEK293 cells were transfected with either WT or mutant *BMAL1* along with a *HIF2A* vector. A luciferase reporter assay was performed to evaluate transcriptional activation by the BMAL1–HIF2A complex of the *AREG* promoter, which contains a shared binding site. *n* = 4 independent experiments. For **f**,**h** and **i**, data are mean ± s.e.m. **j**, Schematic illustrating the substantial structural rearrangement of BMAL1 (red) when accommodating various partners to be involved in different pathways. The PAS domains of BMAL1 (red) bend towards nearly opposite direction and position separately when intertwining with HIF2A (purple). Statistical analysis was performed using one-way ANOVA (**f**, **h** and **i**). The diagram in **j** was created using BioRender. Source Data

**Extended Data Fig. 9:**
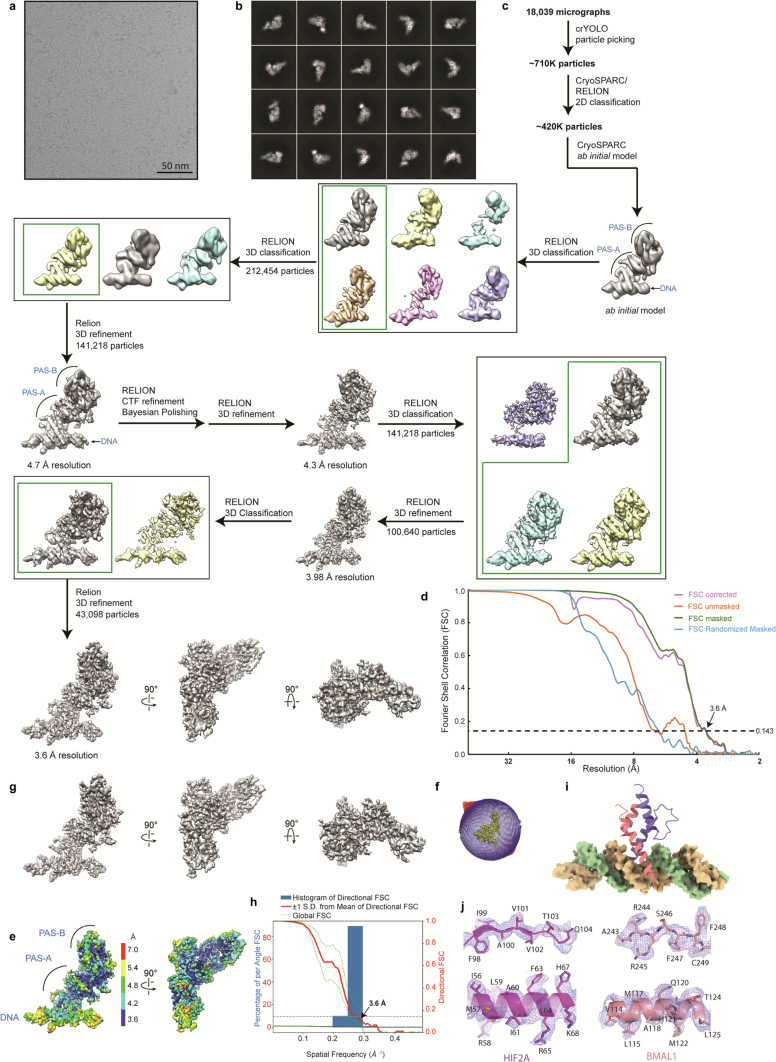
Cryo-EM structure determination of the BMAL1/HIF2A/DNA complex. **a**, A cryo-EM micrograph of the BMAL1/HIF2A/DNA complex. **b**, Representative 2D class averages of the BMAL1/HIF2A/DNA complex. **c**, Flowchart of image processing of the BMAL1/HIF2A/DNA cryo-EM data. **d**, FSC curves of the BMAL1/HIF2A/DNA complex. The resolution for the BMAL1/HIF2A/DNA complex is 3.6 Å according to the gold-standard FSC = 0.143 criterion. **e**, Cryo-EM density map of the BMAL1/HIF2A/DNA complex coloured by local resolution. **f**, Angular distribution plot. **g**, Density map of the BMAL1/HIF2A/DNA complex generated by deepEMhancer. **h**, Histogram and directional FSC plot generated by 3DFSC indicates that 90% of directions achieve a resolution better than 4 Å. **i** and **j**, Local structures with their corresponding densities are shown.

Although the interaction between BMAL1 and HIF2A has been reported^38,44,45^, the structural information of their heterodimerization remains unclear. To determine the structure using single-particle cryo-EM, we expressed and purified the N-terminal regions of BMAL1 and HIF2A, including the bHLH and PAS domains, bound to a 22-bp DNA containing a canonical HRE element (Fig. 2g, Extended Data Fig. 9a and Supplementary Table 11). After two-dimensional (2D) and three-dimensional (3D) classification, the final approximately 43,000 polished particles were processed for 3D refinement, resulting in a density map with an average resolution of 3.6 Å (Fig. 5a and Extended Data Fig. 9b–h). In brief, HIF2A exhibited a better overall resolution compared with BMAL1, probably due to its compact structure and stabilization by BMAL1. An atomic model of the DNA-bound heterodimer was built into the density map using the structures of BMAL1 and HIF2A from the BMAL1–CLOCK^8^ and HIF2A–HIF1B^40^ complexes as templates, respectively (Extended Data Fig. 9i,j and Supplementary Table 11).

**Extended Data Fig. 10:**
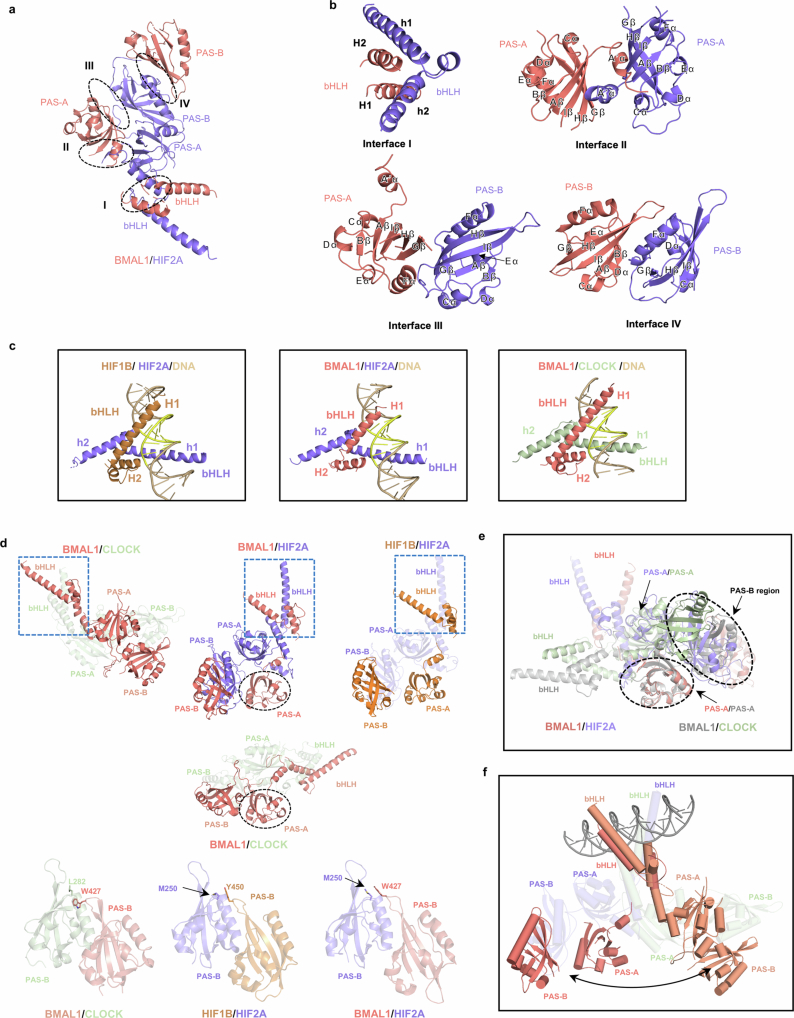
Structural analysis of the BMAL1/HIF2A/DNA complex. **a**, Four domain interfaces (I to IV) between BMAL1 and HIF2A. Each of these is indicated by a dashed ellipse. **b**, Zoom-in views of the interfaces (I to IV) between HIF2A and BMAL1. **c**, Comparison of the DNA-binding by two bHLH domains in HIF1B/HIF2A (left, PDB ID 4ZPK), BMAL1/HIF2A (middle), and BMAL1/CLOCK (right, PDB ID 4H10). The DNA contacted by the bHLH domains is highlighted in yellow. The PAS domains are omitted for clarity. **d**, Structural comparison of BMAL1/HIF2A with BMAL1/CLOCK (PDB ID: 4F3L) and HIF1B/HIF2A (PDB ID: 4ZPK). Upper: Structural comparison of BMAL1/HIF2A with BMAL1/CLOCK and HIF1B/HIF2A by aligning bHLH domains (highlighted in blue dashed frames). For clarity, DNA was omitted. Middle: Structure of BMAL1/CLOCK with its BMAL1 PAS-A domain (dashed circle) aligned to that of BMAL1/HIF2A. Lower: Structural comparison of the PAS-B dimers of BMAL1/HIF2A, BMAL1/CLOCK and HIF1B/HIF2A. The conserved residues in the HI loop and their interacting residues are shown. **e**, Structural comparison of BMAL1/HIF2A and BMAL1/CLOCK by aligning the PAS-A domains of BMAL1. BMAL1 is coloured in red in the BMAL1/HIF2A complex and in grey in the BMAL1/CLOCK complex. **f**, BMAL1 undergoes structural rearrangements upon binding with various partners. Superimposing the BMAL1/HIF2A and BMAL1/CLOCK complexes by aligning their bHLH domains reveals that BMAL1 (red) undergoes a substantial conformational change, with the two PAS domains bending in nearly opposite directions. BMAL1 exhibits a compact overall architecture when bound with CLOCK (green) and a distinctly separated conformation when interacting with HIF2A (purple).

In the structure, BMAL1 and HIF2A exhibit distinct architectures (Fig. 5b). HIF2A adopts a compact conformation, with the PAS-A domain bridging the bHLH and PAS-B domains, whereas the corresponding domains in BMAL1 are arranged more loosely, lacking interdomain contacts (Fig. 5c). BMAL1 uses its bHLH and PAS domains to wrap around their respective counterparts in HIF2A, establishing four major interfaces (I to IV) (Fig. 5d and Extended Data Fig. 10a,b). Similar to classical bHLH transcription factors, the BMAL1–HIF2A heterodimer binds to the DNA through two α-helices, H1 and h1, from their bHLH domains, inserting into the major groove^46^ (Extended Data Fig. 10c). Owing to slight mobility, the density of the DNA bases is not well resolved in the map.

Notably, the structure of BMAL1 bound to HIF2A differs significantly from that of its complex with CLOCK^8^, in which the PAS domains of BMAL1 are tightly packed together (Extended Data Fig. 10d). By contrast, in the BMAL1–HIF2A structure, the PAS domains of BMAL1 are positioned separately and arranged in a conformation similar to the HIF1B observed in its complex with HIF2A. The conformational similarity between BMAL1 and HIF1B is probably due to their significant sequence identity (44%) in the N-terminal region, where several residues involved in HIF2A binding are conserved (Extended Data Fig. 10d and Supplementary Fig. 10). As both BMAL1–CLOCK and BMAL1–HIF2A complexes bind to DNA through their bHLH domains, we superimposed the bHLH domain of BMAL1 and observed that its PAS domains bend in nearly opposite directions. Similarly, superimposing the PAS-A domains of BMAL1 reveals conformational variability in the bHLH and PAS-B domains (Extended Data Fig. 10e,f). These structural comparisons suggest that BMAL1 undergoes significant conformational changes to accommodate different partners (Supplementary Video 1). Notably, proteins with PAS domains in the bHLH family typically form alpha–beta dimers^47^. In the BMAL1–HIF2A structure, two PAS-B domains form an open end-to-end alpha–beta dimer, with the conserved Trp427 in BMAL1 interacting with Met250 in HIF2A to stabilize the PAS-B dimer (Extended Data Fig. 10d). However, it is unclear whether this interaction allows HIF2A to bind to other factors, similar to the PAS-B interaction between CLOCK and CRY1^8,48^.

To strengthen our structural observations, we mutated conserved residues within each domain of BMAL1. These mutations disrupted BMAL1–HIF2A complex formation both in vitro and in HEK293 cells under hypoxia, underscoring the essential role of each domain in heterodimer stability (Fig. 5e–h). Using a luciferase-reporter assay with a human *AREG*-gluc reporter containing the common binding sequence (CAGGTG), we observed that BMAL1 mutants significantly impaired BMAL1–HIF2A transactivation at the *AREG* promoter, underscoring the importance of proper heterodimerization for *AREG* induction (Fig. 5i). Collectively, our study provides the structural basis of the DNA-bound BMAL1–HIF2A heterodimer, highlighting its role in regulating target genes, such as *AREG*, and the capability of BMAL1 in bridging diverse pathways through structural rearrangements for partner binding (Fig. 5j).

## Discussion

Our study underscores the pivotal role of the BMAL1–HIF2A heterodimer in mediating circadian-dependent cardioprotection. This conclusion is supported by robust evidence, including the cryo-EM structure, the diurnal interaction between BMAL1 and HIF2A under hypoxia and during IRI, their functional roles in the synergistic transactivation of *AREG* and their interdependence in mediating circadian-dependent cardioprotection. However, we cannot conclusively prove that BMAL1–HIF2A heterodimerization is the bona fide mechanism regulating the time-of-day dependence of myocardial injury. Although our findings indicate that cardiomyocyte HIF1A does not significantly affect circadian variations in myocardial injury, the established interaction between BMAL1 and HIF1A^31,49^ warrants further investigation into whether BMAL1–HIF1A interactions in other cells contribute to circadian myocardial injury. Moreover, while we demonstrate that BMAL1 enhances HIF2A transcriptional activity and stabilizes it by reducing ubiquitination, the precise mechanisms remain unclear. For example, it is uncertain whether BMAL1 directly regulates HIF hydroxylation or influences HIF2A levels through alternative post-translational modifications. Finally, the contributions of other circadian rhythm molecules, such as PER2, and their interactions with HIFs may also have a role in this phenomenon^27^. Further elucidation of these pathways could advance chronotherapeutic strategies to optimize HIF-targeted interventions^13–15^. The widespread presence of peripheral molecular clocks across various cardiac cell types^21^ emphasizes the need to define BMAL1’s tissue-specific roles for coordinated cardiac protection. Our BMAL1–HIF2A–DNA structure reveals BMAL1’s ability to undergo structural rearrangements to bind a non-canonical partner, enabling cross-talk between signalling pathways. It also provides a molecular basis for developing therapeutics to stabilize this interaction, offering opportunities for treating ischaemic heart disease. Given the feasibility of integrating circadian timing into clinical practice, prospective trials are needed to evaluate whether aligning treatments with circadian phases or directly targeting circadian rhythms can improve outcomes in patients with MI. Beyond MI, circadian modulation of hypoxic responses may have broader implications for other hypoxia- and inflammation-related diseases with circadian characteristics, potentially paving the way for clock-based therapeutic innovations.

## Methods

Detailed information on experimental materials, reagents and mouse lines, including sources and identifiers, is provided in the key resources table in Supplementary Table 12.

### Human participant research

#### Study design and participants

We examined samples from a prospective study of myocardial injury in humans undergoing cardiac surgery (ClinicalTrials.gov: NCT00281164). The study population consisted of consecutive patients (aged ≥20 years) with aortic stenosis referred to our cardiovascular surgery department at Brigham and Women’s Hospital for aortic valve replacement (with or without coronary artery bypass graft) between 1 January 2009 and 31 December 2014. Patients enrolled in a concurrent drug or device trial were excluded. This ongoing study involved 56 patients in the morning (samples collected between 08:00 and 12:00; median time, 10:32) and 17 patients who underwent the same procedure in the afternoon (samples collected between 15:00 and 21:00; median time, 17:15). Patients whose surgery fell outside of these time periods were excluded from the analysis. The ethics committee for the Protection of Human Subjects from Research Risks of Brigham and Women’s Hospital approved the protocol, and written informed consent was obtained from all of the patients.

#### Procedures

Patients underwent aortic valve replacement in the morning or afternoon by the same senior surgeon. Anaesthesia, cardiopulmonary bypass, cardioplegia and surgical procedures were done according to standard guidelines. Anaesthesia was induced with intravenous fentanyl or sufentanil and propofol (0.5–1.5 mg per kg) and maintained with isoflurane. Surgery was done using normothermic cardiopulmonary bypass and repeated antegrade and retrograde cold blood cardioplegia. LV biopsy samples were obtained from both the morning and afternoon patient cohorts before and after approximately 80 min of aortic cross-clamping—a procedure that temporarily halts blood flow to the heart. The samples were taken from the site of the routinely placed LV vent.

### Animal care and use

Animal care was performed according to the guide for the care and use of laboratory animals of the National Institutes of Health. All experimental procedures were approved by the UTHealth Institutional Animal Care and Use Committee. The sample size was estimated based on published literature on previous murine models of myocardial IRI^27^. All of the mice were housed under a standard 12 h–12 h light–dark photoperiod at 22 °C with ad libitum access to a standard chow diet, and maintained at a humidity level of 40–60%.

### Generation of transgenic mouse models

To generate cardiomyocyte-specific deletion, myosin-Cre^+^ (*A1cf*^Tg[Myh6-cre/Esr1*]1Jmk^*/*J, The Jackson Laboratory, 005650), *Hif1a*^*loxP/loxP*^ (*B6.129-Hif1a*^*tm3Rsjo*^*/J*, The Jackson Laboratory, 007561), *Hif2a*^*loxP/loxP*^ (*Epas1*^*tm1Mcs*^/J, The Jackson Laboratory, 008407), *LSL-HIF2dPA* (*Gt(ROSA)26Sor*^tm4(HIF2A*)Kael^*/J*, Jackson Laboratory, 009674), and *Bmal1*^*loxP/loxP*^ (B6.129S4(Cg)-*Bmal1*^*tm1Weit*^/J, The Jackson Laboratory, 007668) mice, aged 8 weeks, were purchased from The Jackson Laboratory and crossbred as previously described^16,27^. To address sex as a biological variable, both male and female mice aged 8 to 16 weeks underwent myocardial ischaemia and reperfusion surgery. Our sex-specific analysis, which included assessments of infarct size, troponin levels and cardiac function, revealed no significant differences between sexes^29,50^. To induce Cre-recombinase activity, mice received an i.p. injection of tamoxifen at a dosage of 1 mg per day for five consecutive days, as previously described^29,30,50^. A recovery period of 7 days was allowed after the final tamoxifen dose before proceeding with further experimental procedures. In these experiments, myosin-Cre^+^ mice were used as controls. Moreover, *Areg*^*−/−*^ mice (*B6;129-Areg*^*tm1Dle*^*/Mmnc*, MMRRC, MMRRC_011533-UNC)^51^ aged 8 weeks were purchased from Mutant Mouse Resources & Research Centers. To circumvent lactation difficulties observed in younger female mice, *Areg*^*−/−*^ mice were bred using a heterozygous (*Areg*^*+/−*^) strategy^16,41^. Control mice for these experiments were C57BL/6J (The Jackson Laboratory, 000664) mice aged 8 weeks, chosen for their same genetic background as the *Areg*^*−/−*^ mice. Genotyping for all mouse strains was conducted by GeneTyper (NY).

### Mouse model of myocardial IRI

The mouse model used in the study was subjected to a period of entrainment for at least 2 weeks within circadian cabinets (Actimetrics) to establish a stable circadian rhythm^42^. After this acclimatization period, the mice underwent an in situ procedure for myocardial IRI at different times of day (ZT2, ZT8, ZT14 or ZT20), as described in previous publications^27,29,30,50,52^. In brief, the mice were anaesthetized using 1–3% isoflurane delivered by a compact small-animal anaesthesia device (RWD), followed by endotracheal intubation and ventilation using a VentStar Small Animal Ventilator (RWD). Once anaesthetized, the mice were placed into a supine position and received pre-incisional analgesia with sustained-release buprenorphine (0.1 mg per kg, subcutaneous; ZooPharm). Throughout the procedure, their body temperature was consistently maintained at 37 °C with a ThermoStar Homeothermic Blanket equipped with rectal feedback control (RWD). The surgical procedure was meticulously performed using a research stereomicroscope system, SZX10 (Olympus). We temporarily ligated the proximal left anterior descending coronary artery approximately 2 mm from its emergence beneath the left atrium, using Surgipro II 7–0 monofilament polypropylene sutures (Covidien). The success of this ligation was confirmed by observing blanching or a pale discolouration of the anterior wall of the heart, a reduction in wall movement and the presence of ST-segment elevation on an electrocardiogram (ECG). After 45 min of induced ischaemia, reperfusion was initiated to restore the blood flow. The effectiveness of reperfusion was verified both by the resolution of ST-segment elevation on the ECG and through direct visual inspection, where a return of colour and movement in the previously affected area of the heart was noted. After surgery, the mice were carefully transferred back to the circadian cabinets for recovery and continued observation. Any mice that did not survive within the first 48 h after MI were considered to have experienced technical complications and were consequently excluded from further analysis. Blinding was implemented for the individuals analysing the data, including those calculating infarct size and measuring serum troponin I levels, to ensure unbiased data interpretation. However, blinding the surgeons performing the surgeries was logistically challenging due to the nature of the procedures. Despite this, the surgeries were carried out by experienced surgeons, ensuring consistency. To confirm the similarity of conditions, we calculated the AAR/LV, which showed comparable values across ZT and treatment/vehicle groups. This suggests that any differences observed in the study are largely due to experimental conditions rather than variations in the surgical procedure.

### Cell line

All cell lines and primary cells used in this study, including HEK293 cells (ATCC, CRL-1573) and human primary cardiomyocytes (ScienCell Research Laboratories, 6200), were authenticated by short-tandem-repeat profiling, as performed by the manufacturers. Moreover, the cell lines were tested for mycoplasma contamination by the manufacturers and were subsequently tested monthly for potential contamination throughout the study. All mycoplasma contamination tests yielded negative results. Furthermore, none of the cell lines used in this study are listed in the database of commonly misidentified cell lines maintained by the International Cell Line Authentication Committee (ICLAC) and NCBI Biosample.

### Timed administration of AREG and NOB in mouse models

#### Timed administration of AREG

To simulate the clinical scenario in which treatment is initiated after the onset of MI, mouse recombinant AREG protein (R&D Systems) was prepared in 0.9% NaCl solution, and a dosage of 10 µg AREG was administered at the start of the reperfusion phase. Furthermore, to assess the long-term effects of timed AREG treatment on cardiac function, a post-injury treatment regimen was implemented. In this protocol, AREG (10 µg) was administered through i.p. injection at either ZT8 or ZT20 daily for the first 3 days after myocardial IRI. For control purposes, an equivalent volume of 0.9% NaCl solution was administered as a vehicle.

#### Administration of NOB

In the NOB treatment experiments, Mice were administered either DMSO (as a vehicle control) or NOB at a dose of 200 mg per kg body weight. The administration was carried out through i.p. injection and repeated on an every-other-day basis. This treatment protocol was followed for 2 weeks before the surgical procedure and was specifically timed within the ZT14–ZT20 time window. To evaluate the long-term effects of NOB on cardiac function and remodelling, the same dosing regimen was continued after IRI. The chosen dosing regimen was based on several considerations: first, the dosage range was consistent with those used in previous studies (100–200 mg per kg per day)^42,53^, and it was aligned with the active phase of the mice. Second, daily dosing was intentionally avoided to prevent the entrainment of the experimental mice to the dosing schedule as an artificial zeitgeber. Third, previous single-dose pharmacokinetic assays demonstrated a favourable pharmacokinetic profile for NOB, with significant exposure detected in the serum, brain and liver^42^. Considering that NOB levels were typically undetectable within 8–24 h after administration^42,54^, we opted for an every-other-day dosing strategy to prevent incomplete clearance over the 4-week experimental period.

### Mouse RNA-seq and bioinformatic analysis

The AAR of the heart was collected after 2 h of reperfusion from the C57BL/6J mice subjected to myocardial IRI at either ZT8 or ZT20. Total RNA was extracted using the RNeasy Mini kit (Qiagen) and was used to construct RNA-seq libraries, which were then sequenced using the Illumina 1.9 platform (75 bp paired end). Alignment of RNA sequencing tags was restricted to those mapping to the same DNA strand as annotated in the GRCm38 reference genome, using STAR (v.2.7.10a). Quantification of transcripts was conducted by calculating the fragments per kilobase of transcript per million mapped reads (FPKM) values, along with transcript counts. In the preprocessing step of mRNA analysis, any gene identified as non-expressed, defined as having an FPKM expression level less than 1 in more than 80% of samples, was excluded. The normalized expression profiles of these mRNAs were then subjected to PCA for quality control and to evaluate sample similarity. Differential expression analysis was performed using the DESeq2^55^ pipeline, with DEGs being identified based on a threshold of 1.5-fold change and an adjusted *P* *<* 0.05, as determined using the Benjamini–Hochberg method^56^.

The functional enrichment analysis of the identified DEGs was conducted using the GO and KEGG pathway databases with the WebGestalt tool^57^ (http://www.webgestalt.org/; v.2019). For rigorous validation, only pathways and GO terms with an adjusted *P* < 0.05 were included. In constructing the gene regulation network, we used known transcription factor (TF)–mRNA interactions from the TRRUST_v2^58^ and Chipbase_v2^59^ databases. Moreover, the mouse protein–protein interaction (PPI) network was integrated from the STRING database (v.11.5)^60^, with a focus on interactions having a combined score above 900. Considering the limited sample size at each timepoint, we merged six samples from both conditions to calculate the Spearman’s correlation coefficient (SCC), ensuring robust and meaningful analysis. In finalizing the gene regulation network, we merged the TF–mRNA regulation network with the PPI data, specifically targeting interactions where both genes were differentially expressed. We also selectively incorporated edges with high SCC into our network, tailored to align with the specific conditions of our experimental design.

### Human RNA-seq and bioinformatic analysis

For human RNA-seq and subsequent bioinformatic analysis, we began by excluding outlier samples that failed to cluster appropriately, resulting in a total of 73 samples for analysis, including 56 morning and 17 afternoon samples derived from various sequencing protocols. Quality control of raw sequencing reads identified adaptor sequences from the Illumina Nextera platform in some samples, which were subsequently trimmed using Cutadapt (v.4.1)^61^. kallisto (v.0.46.1)^62^ was used to quantify transcript-level expression by mapping to a transcript index built from GENCODE human transcript (v.44)^63^. We next used tximport to convert these transcript-level quantifications to gene read counts^64^. Differential expression analysis was performed with DESeq2 (v.1.34.0)^55^, with particular attention paid to sequencing batch to control for batch effects. Genes displaying a log_2_-transformed fold change of greater than 0.5 and *P* < 0.01 were deemed to be significant. For enrichment analysis, we used the GO annotation (v.1.1) for humans, using all genes annotated by at least one GO term^65^ as the background in Fisher’s exact test to identify over-represented biological processes. Moreover, the R package KEGGREST (v.1.36.0) was used to extract KEGG pathway annotations, and Cytoscape (v.3.10.0) was used to construct network plots for the identified KEGG pathways.

### Microarray data reanalysis

The microarray assay for gene expression transcript levels of post-ischaemic myocardium from myosin-Cre^+^ or *Hif2a*^*loxP*/*loxP*^ myosin-Cre^+^ mice was reanalysed using data obtained from the GEO (GSE67308)^16^. Differential expression analysis was performed using GEO2R, with limma precision weights applied and the remaining options set to default^66^.

### Assessment of infarct size in the acute phase

The assessment of myocardial infarct size was conducted by determining the percentage of infarcted myocardium within the AAR using a previously established method^16,27,29,30,50^. After 2 h or 1 day of reperfusion, the hearts were flushed with PBS and then subjected to permanent occlusion of the left coronary artery. 1 ml of 1% Evans blue dye (Sigma-Aldrich, E2129) was then infused through the carotid artery catheter. After infusion, the hearts were excised and sectioned into 1 mm slices using a microtome (Roboz, SA-4130). These heart sections were subsequently double-stained with 1% TTC (Sigma-Aldrich, T8877) for 10 min at 37 °C and then fixed in 10% formalin overnight. The double-stained heart slices were photographed, and ImageJ software (NIH, Fiji v.2.1.0) was used to calculate the infarct size.

### Cardiac troponin I enzyme-linked immunosorbent assay

Serum samples were collected from mice subjected to myocardial IRI at various ZTs through the inferior vena cava after 2 h of reperfusion. The measurement of serum troponin I, a highly sensitive biomarker for cardiac injury, was conducted using the mouse cardiac troponin-I SPARCL kit (Life Diagnostics, CTNI-SP-1), as we have done previously^16,27,29,30,50^.

### STE analysis of mouse cardiac function

#### Ultrasound imaging

Cardiac function and structure were assessed by transthoracic echocardiography using the Vevo3100 Ultrasound system (VisualSonics). Mice were lightly sedated with 0.5–1.0% isoflurane and positioned on a heated platform equipped with ECG monitoring. Validation criteria included consistent, uninterrupted tracking of the endocardium throughout the cardiac cycles, maintaining a heart rate between 450 and 550 beats per minute, and a frame rate exceeding 250 fps. Two-dimensional grey-scale echocardiographic images were obtained from parasternal long-axis and short-axis views. Longitudinal strain represented myocardial shortening at the endocardium, while radial strain indicated shortening at the mesocardium. All image acquisitions and subsequent offline measurements were conducted by a single investigator who was blinded to the grouping of the animals.

#### Speckle-based deformation mapping and analysis

For strain analysis using Vevo LAB (FUJIFILM VisualSonics, v.5.7.1), we carefully selected suitable *B*-mode loops, ensuring a clear view of the endocardial border and no image artefacts. Three consecutive cardiac cycles with distinct ECG recordings were chosen for in-depth analysis. Semi-automated tracing of the endocardial and epicardial borders was performed, with adjustments made as necessary to maintain optimal tracking quality throughout each cine loop. The tracked images were then processed frame by frame for strain measurements^34,67,68^. The resulting strain values were averaged across these cardiac cycles, providing comprehensive data on the LV systolic function (EF, FS and GLS), LV size (end-diastolic volume and end-systolic volume) and LV mass (end-diastolic LV mass and end-systolic LV mass).

In analysing regional and global cardiac dynamics, such as contractility and synchronicity, the LV endocardium was delineated using 48 sampling points^34,67^. This method divided the chamber into six segments for detailed examination in the long-axis view: basal anterior, mid anterior, apical anterior, basal posterior, mid posterior and apical posterior segments^67^. In myocardial IRI models, the mid anterior, apical anterior and apical posterior wall segments were designated as the infarct region, and the remaining segments were categorized as the non-infarct region^34^. Peak systolic strain, indicating the percentage change in length during myocardial contraction and relaxation, was calculated using the formula: *ε* = (*L*_1_ − *L*_0_)/*L*_0_, where *L*_0_ is the initial length and *L*_1_ is the final length^69^. This calculation was applied to each segment to evaluate the peak systolic strain (%) and the time-to-peak systolic strain (ms). Abnormal ventricular contractility patterns were evaluated on the basis of the magnitude of systolic strain (segment peak strain) and its timing (peak of shortening). Dyssynchrony was specifically identified by a pattern of reduced systolic strain magnitude, early opposing deflection and a delayed time-to-peak systole. Akinesis was defined as minimal or no contractility, with peak systolic strain values between −5% and 5%. Dyskinesis was described as systolic motion of the ventricle occurring in the opposite direction of normal contraction. Moreover, intraventricular disparity was measured by assessing the intraventricular delay in the time-to-peak strain and the s.d. of the time-to-peak strain across segments^34,70^.

### Sequential hypoxia treatment after synchronization in vitro

HCMs were obtained from ScienCell Research Laboratories (ScienCell, 6200) and isolated from human hearts^71^. The cells were synchronized using 200 nM dexamethasone, which was replaced with complete media (ScienCell, 6101) after 1 h. After synchronization, we initiated a series of hypoxia treatments, exposing different batches of cells to 1% oxygen for 4 h at systematic 4-h intervals to capture cellular responses at various circadian phases. The first hypoxia session started immediately after synchronization (CT0) and lasted for 4 h (referred to as the CT4 timepoint). Subsequent treatments were applied every 4 h: from CT4 to CT8 (CT8 timepoint), and so on, continuing up to CT40. Immediately after each hypoxia period, cells were collected to evaluate transcript and protein levels.

To ensure valid results, we carefully selected timepoints for post-synchronization analysis. After synchronization, cells require a stabilization period to re-establish their circadian rhythms, and early evaluations may capture this adjustment phase rather than true circadian behaviour^72^. Moreover, dexamethasone induces immediate stress responses and activates signalling pathways unrelated to circadian changes^73,74^. Administering hypoxia treatments too soon after synchronization could overlap these stress responses, confounding the circadian-related changes we aim to measure. For this reason, early timepoints like CT8 were excluded, and we focused on later timepoints to minimize these effects and ensure that the data reflect circadian-driven changes.

### ChIP–qPCR

HCMs were synchronized by dexamethasone (200 nM) for 1 h and then exposed to normoxia or hypoxia (1% oxygen) for 4 h at CT20 and CT32 in Fig. 3q. HEK293 cells were transfected with pcDNA3-mBmal1 (a gift from A. Sancar, Addgene, 31367)^75^ and treated with normoxia or hypoxia (1% oxygen) for 4 h in Fig. 3s. ChIP–qPCR was conducted using the SimpleChIP enzymatic chromatin IP kit (Cell Signaling Technology, 9002). In brief, cells were fixed with 1% formaldehyde, quenched with 125 mM glycine, washed and sonicated to digest the DNA to an average length of 150–500 bp. Lysates were then incubated overnight at 4 °C with 2 µg of ChIP grade anti-HIF2A antibody (Novus Biologicals, NB100-122) and anti-BMAL1 antibody (Cell Signaling Technology, 14020), anti-HIF1B antibody (Cell Signaling Technology, 5537) or IgG as a negative control. After washing, the antibody–protein–DNA complexes were eluted from the beads. The cross-links were then reversed through overnight incubation at 65 °C, followed by DNA purification. For the qPCR analysis, specific primer pairs were designed to amplify the predicted common binding sequence (CAGGTG) on the human *AREG* promoter region or E-box regions on the human *HIF2A* promoter region. The enrichment was quantitatively compared against input controls and IgG negative controls to confirm the specificity of the ChIP-qPCR assay.

### Co-IP analysis

HEK293 cell lines (Fig. 2b and Extended Data Fig. 2b) overexpressing the pcDNA3-mBmal1 (a gift from A. Sancar, Addgene, 31367)^75^ were exposed to either normoxia or hypoxia (1% oxygen) for 4 h. In Fig. 3r, HCMs were initially synchronized using dexamethasone (200 nM) for 1 h and then exposed to hypoxia (1% oxygen) for 4 h at CT20 and CT32. After exposure, the cytoplasmic and nuclear fractions were separately extracted using the NE-PER Nuclear and Cytoplasmic Extraction Kit (Thermo Fisher Scientific, 78835), according to the manufacturer’s instructions. The cell lysates were centrifuged and pre-cleared with protein A/G beads (Thermo Fisher Scientific, 53133) for 1 h with rotation. For the IP, the pre-cleared lysates were incubated overnight at 4 °C with specific antibodies: mouse anti-Flag (Sigma-Aldrich, F1804) or rabbit anti-HIF2A (Novus Biologicals, NB100-122) antibodies. Mouse IgG (Cell Signaling Technology, 5415) and rabbit IgG (Abcam, ab172730) were used as isotype controls for the respective antibodies. After the overnight incubation, 40 μl of protein A/G beads were added to each sample, and the incubation was continued for an additional 4 h at 4 °C. The proteins were then eluted using an SDS sample buffer and subjected to heating at 95 °C for 5 min with vigorous shaking. The eluted proteins were subsequently stored at −80 °C for further analysis. In Fig. 2d, HCMs were subjected to hypoxic conditions (1% oxygen) for various durations: 0, 0.5, 1, 2, 4 or 8 h. After each exposure period, HCMs from a single 10-cm dish were lysed using 330 µl of HEPES extraction buffer containing 20 mM HEPES pH 7.4, 100 mM NaCl, 1 mM EDTA, 0.1% Triton X-100, 5% glycerol, and a cocktail of protease, phosphatase and RNase inhibitors. Co-IP was then performed according to the previously described protocol. In Extended Data Fig. 7w,x, HEK293 cells were transfected with UB–HA, HIF2A–MYC or BMAL1–Flag expression constructs. To assess the ubiquitination of HIF2A, cells were treated with 10 µM MG132 for 16 h. After treatment, the cells were collected and lysed using HEPES extraction buffer. Immunoprecipitation was then performed using rabbit anti-HIF2A antibodies (Novus, NB100-122) as previously described.

### CHX chase assay

HCMs were transduced with Bmal1-AAV (10^5^ GC per cell) or shBMAL1-AAV (10^5^ GC per cell) and exposed to 1% O_2_ for 4 h to stabilize HIF1A and HIF2A. After the return to normoxic conditions, CHX (20 μg ml^−1^, Sigma-Aldrich, C4859) was added to inhibit de novo protein synthesis. Cells were collected at 15, 30, 60, 90 and 120 min after CHX treatment, washed with cold PBS, and lysed in RIPA buffer containing protease and phosphatase inhibitors. After centrifugation, protein concentrations were determined using a BCA protein assay kit. Equal amounts of protein (10–20 μg) were separated by SDS–PAGE and probed with primary antibodies against HIF2A (Novus Biologicals, NB100-122, 1:1,000), HIF1A (Novus Biologicals, NB100-479, 1:1,000), BMAL1 (Cell Signaling Technology, 14020, 1:1,000) and β-actin (Cell Signaling Technology, 4967, 1:1,000). The relative protein levels at each timepoint were quantified using ImageJ (NIH, v.2.1.0) and normalized to β-actin as a loading control. For each experimental group, normalized protein levels at 15, 30, 60, 90 and 120 min were expressed as the percentage of the 0-min timepoint (set as 100%). The degradation rates of the proteins were then plotted as the percentage of remaining protein over time.

### PLA

Confluent HCMs were subjected to hypoxic conditions (1% oxygen) for various durations (0, 1, 4 or 8 h). After exposure, the cells were fixed using 4% paraformaldehyde (Millipore Sigma, P6148), permeabilized with 0.1% Triton X-100 (Amresco, M143), and subjected to a blocking step to reduce non-specific binding. Rabbit anti-BMAL1 (Abcam, ab3350, 1:100), mouse anti-HIF2A (Novus Biologicals, NB100-132, 1:100) and mouse anti-HIF1A (Novus Biologicals, NB100-105, 1:100) were then applied. For PLA, pairs of primary antibodies raised in different species were used as mentioned. After primary antibody incubation, cells were treated with Duolink In Situ PLA Probes (Millipore Sigma, DUO92002 and DUO92004) conjugated to these antibodies. These probes, when in close proximity of less than 40 nm, facilitated the ligation of adjacent oligonucleotides attached to them. Subsequently, the ligated oligonucleotides were amplified and detected using fluorescently labelled probes. The resulting fluorescence signals, which are indicative of PPIs, were visualized using a Nikon Eclipse Ti2 confocal microscope (Nikon) and analysed with NIS Element AR software (Nikon, v6.10.01). To confirm the specificity of the observed interactions, control experiments were performed using only the BMAL1 antibody.

### Plasmid construction and site-directed mutagenesis

To overexpress proteins in *Escherichia coli*, the DNA sequences encoding the N-terminal portions of mouse HIF2A (residues 3–361) and mouse HIF1A (residues 13–357), with a 6×His tag at their N terminus, were inserted into the pET15b vector. The DNA encoding the N-terminal region of mouse BMAL1 protein (residues 68–488), with a C-terminal Flag tag, was ligated into the pET24b vector.

For GST pull-down, the DNA encoding residues 3–361 of mouse HIF2A was inserted into pGEX-6P-1 to generate GST–HIF2A for expression. Six BMAL1 single-site mutants (L95E, L115E, L150E, F248D, R343A and F423D) and three double-site mutants (L95E/L115E, L150E/F248D and R343A/F423D) were generated using site-directed mutagenesis. The DNA sequences of all of the mutants were verified by DNA sequencing.

### Protein expression and purification

The recombinant plasmids were individually transformed into *E. coli* BL21 Rosetta (DE3) or co-transformed together to express either individual proteins or the BMAL1–HIF2A complex. Expression of the proteins or BMAL1–HIF2A complex was carried out at 18 °C by induction with 0.1 mM isopropyl-β-d-thiogalactoside for 16 h. The BMAL1–HIF2A heterodimer (including bHLH, PAS-A and PAS-B domains) was first purified through a Ni-NTA affinity column (Thermo Fisher Scientific, 88222) followed by heparin chromatography. The heterodimer eluted from the heparin column was further analysed by size-exclusion chromatography using the Superdex 200 Increase 10/300 GL column. The peak fractions were assessed using SDS–PAGE and Coomassie blue staining. Subsequently, the samples were combined and concentrated. The concentrated protein was then used for cryo-EM sample preparation or stored at −80 °C for further use. His–HIF2A and His–HIF1A were purified individually using Ni-NTA affinity columns, followed by dialysis to eliminate imidazole. The proteins were concentrated and stored at −80 °C for further use.

### Pull-down assay

To confirm the interaction between BMAL1 and HIF2A or HIF1A in Fig. 2h, BMAL1-expressing cells were lysed using binding buffer (20 mM HEPES (pH 7.4), 10% glycerol, 0.01% Triton X-100, 300 mM NaCl, 5 mM MgCl_2_, 1 mM DTT, and protease inhibitors). After centrifugation, the supernatants were incubated with anti-Flag M2 beads (Millipore Sigma, A2220) for 40 min at 4 °C. Subsequently, the beads were washed three times using the binding buffer, followed by the addition of purified His–HIF2A or His–HIF1A proteins. After incubation for 40 min at 4 °C, the beads were washed five times using the binding buffer. The proteins bound to beads were then eluted using Flag peptide (0.3 mg ml^−1^) and further analysed by SDS–PAGE and western blotting using anti-Flag (Sigma-Aldrich, F1804, 1:1,000) and anti-His (Thermo Fisher Scientific, MA1-21315, 1:1,000) antibodies.

To compare the interactions between HIF2A and WT BMAL1 or BMAL1 mutants in Fig. 5e, the cell pellets of GST–HIF2A and GST (as a control) were lysed by sonication in binding buffer (20 mM HEPES (pH 7.4), 10% glycerol, 0.05% Triton X-100, 300 mM NaCl, 5 mM MgCl_2_, 1 mM DTT and protease inhibitors). After centrifugation, the supernatants were incubated with Glutathione magnetic agarose beads (GE Healthcare, 17-0756-01) for 30 min at 4 °C. The beads were subsequently washed three times using the binding buffer, followed by the addition of Flag-tagged WT or mutated BMAL1. After 40 min incubation at 4 °C, the beads were washed five times using the binding buffer. The proteins bound to beads were eluted by heating with SDS–PAGE loading dye at 95 °C. The eluted samples were further analysed by SDS–PAGE and western blotting using anti-GST (Genscript, A00865, 1:1,000) and anti-Flag (Sigma-Aldrich, F1804, 1:1,000) antibodies.

### Electrophoretic mobility shift assay

Five fmol of 22-bp biotin-labelled dsDNA fragment containing an HRE consensus sequence was incubated with 1, 2, 3, 4 and 6 pmol of purified BMAL1–HIF2A heterodimer in a total volume of 10 µl in 10 mM MES (pH 6.0), 50 mM KCl, 1 mM DTT, 2.5% glycerol, 0.05% NP-40 and 5 mM MgCl_2_. After incubation at room temperature for 20 min, the binding reactions were directly loaded onto a native 4–20% polyacrylamide gel and electrophoresed in 0.5× TBE buffer. DNA on the gel was transferred onto a positively charged nylon membrane and detected using an HRP-conjugated streptavidin with chemiluminescent substrates.

### Surface plasma resonance

The interaction analysis of the BMAL1–HIF2A heterodimer with 22-bp DNA duplexes (HRE or E-box) was performed using OpenSPR (Nicoya Life Sciences). A 5′-end biotin-labelled dsDNA fragment (2 μM) containing either an HRE or E-box consensus sequence was immobilized in channel 2 of the biotin–streptavidin sensor, while channel 1 remained unmodified and served as a control. The BMAL1–HIF2A heterodimer at various concentrations (from 5 to 50 nM) was injected and flowed slowly at a rate of 20 μl min^−1^ over the sensor chip for 5 min in a running buffer (20 mM MES pH 5.5, 300 mM NaCl, 0.05% Tween-20, 0.02% BSA). After the injection, a 10-min dissociation phase was collected. The resulting data were analysed using Trace Drawer Kinetic Data Analysis software (v.1.9.2), using a one-to-one model, which assumes one monovalent ligand binding to one target.

### Transactivation assay

To assess the transcriptional activation activity of HIF2A and BMAL1 on the promoter of human *AREG*, the 3 × 300 bp sequences (−300/0) from the human *AREG* promoter harbouring the common binding sequence (CAGGTG) were PCR-amplified and cloned into the pEZX-PG04-promoter luciferase vector to generate GLuc-*hAREG*. HEK293 cells were co-transfected with several constructs: pcDNA3-mHif2a-P405A/P530V/N851A (oxygen-regulation insensitive, a gift from C. Simon, Addgene, 44027)^76^, pcDNA3-mBmal1 (a gift from A. Sancar, Addgene, 31367)^75^, *hAREG*-gluc and SEAP vectors (GeneCopoeia, LF031). Transfection was performed using Lipofectamine 3000 reagent (Invitrogen, L3000015). After 48 h, the culture medium was collected, and the transcriptional activity was quantified using the Secrete-pair Dual Luminescence Assay Kit (GeneCopoeia, LF031) on the Cytation5 (Agilent Technologies) device with Gen5 software (v.3.12, Agilent Technologies). To investigate the transcriptional activity of HIF2A and BMAL1 on a general HIF target gene, HEK293 cells were transfected with the *hPGK1*-luc reporter (a gift from C. V. Dang, Addgene plasmid, 128095) along with HIF2A or BMAL1 expression constructs. After 48 h of transfection, firefly and *Renilla* luciferase activities were measured using the Dual-Luciferase Reporter Assay System (Promega, E1690). The luciferase activity was detected on the Cytation5 (Agilent Technologies) system and analysed using Gen5 software (v.3.12, Agilent Technologies).

### Immunofluorescence staining and TUNEL assay

On day 1 after MI, collected heart tissues were fixed in 10% neutral-buffered formalin and then dehydrated through a graded alcohol series. After dehydration, the tissues were cleared in xylene and embedded in paraffin blocks. Sections of 5–6-μm thickness were prepared and underwent antigen retrieval. These sections were then blocked with diluted donkey serum, followed by overnight incubation with primary antibodies at 4 °C. In the immunofluorescence staining process, a range of primary antibodies was used to specifically target and identify various proteins within the tissue sections. These primary antibodies included: AREG (Santa Cruz, sc-74501, 1:50), HIF2A (Novus Biologicals, NB100-122, 1:200; Novus Biologicals, NB100-132, 1:200), BMAL1 (Abcam, ab3350, 1:200), α-sarcomeric (Abcam, ab137346, 1:200), vimentin (Cell Signaling Technology, 5741, 1:200), and α-smooth muscle actin (Cell Signaling Technology, 19245, 1:200) antibodies. For antigen visualization, the sections were then incubated with Alexa fluorescence-conjugated secondary antibodies (Invitrogen). Moreover, TUNEL staining was conducted using in situ Click-iT Plus TUNEL assay kits (Thermo Fisher Scientific, C10617) according to the manufacturer’s protocol to detect apoptotic cells as previously described^77^. The samples were counterstained with DAPI (1 μg ml^−1^, Invitrogen, D3571) and mounted using SlowFade Gold Antifade reagent (Invitrogen, S36936). The stained sections were then imaged with Nikon Eclipse Ti2 confocal microscopy (Nikon) and analysed using ImageJ software (NIH, Fiji v.2.1.0).

### RT–qPCR

Total RNA was extracted from both tissue samples and isolated cells using the RNeasy Mini Kit (Qiagen, 74106) according to the manufacturer’s guidelines. This procedure is consistent with our established protocols as described in previous publications^16,29,50^. After the extraction, cDNA was synthesized from the extracted RNA using the High-Capacity cDNA Reverse Transcription Kit (Thermo Fisher Scientific, 4368814). qPCR was then performed using SYBR Green PCR Master Mix (Qiagen, 204145). The reactions were performed on the Bio-Rad CFX384 Touch Real-Time PCR Detection System. For the analysis of gene expression levels, we applied the comparative *C*_t_ (ΔΔ*C*_t_) method. The final data were presented as mean expression ratios relative to β-actin, allowing for the comparison of gene expression across different samples.

### Western blotting

Mouse tissues and isolated cells were prepared for western blot analysis by lysing in RIPA lysis buffer (Thermo Fisher Scientific, 89900), supplemented with both protease (Thermo Fisher Scientific, 78425) and phosphatase inhibitor cocktails (Thermo Fisher Scientific, 78420). Protein concentrations were quantified, and 10–20 μg of total protein was separated on 4–12% SDS-PAGE gels (Bio-Rad Laboratories). After electrophoresis, the proteins were transferred to membranes for immunoblotting. The membranes were probed with the following primary antibodies: anti-HIF1A (Novus, NB100-105, 1:1,000), anti-HIF2A (Novus, NB100-122, 1:1,000), anti-HIF2A (Bethyl Laboratories, A700-003, 1:1,000), anti-HIF1A (Bethyl Laboratories, A700-001, 1:1,000), anti-HIF1B (Cell Signaling Technology, 5537. 1:2,000), anti-BMAL1 (Cell Signaling Technology, 14020, 1:2,000), anti-CLOCK (Cell Signaling Technology, 5157, 1:1,000), anti-RORα (Abcam, ab60134, 1:1,000), anti-AREG (Santa Cruz, sc-74501, 1:1,000), anti-caspase-3 (Cell Signaling Technology, 9662, 1:1,000), anti-cleaved caspase-3 (Cell Signaling Technology, 9661, 1:1,000), anti-Bax (Cell Signaling Technology, 2772, 1:1,000), anti-Flag (Sigma-Aldrich, F1804, 1:1,000), anti-ubiquitin (Santa Cruz, sc-8017, 1:1,000), anti-lamin B1 (Cell Signaling Technology, 12586, 1:2,000), anti-lamin A/C (Cell Signaling Technology, 4777, 1:2,000), anti-TBP (Cell Signaling Technology, 8515, 1:1,000), anti-α-tubulin (Cell Signaling Technology, 2144, 1:2,000) and anti-β-actin (Santa Cruz, 47778, 1:2,000). After incubation with secondary antibodies, the membranes were developed using appropriate substrates. The signal intensity was detected and quantified using ImageJ software (NIH, Fiji v.2.1.0). The results were normalized to the appropriate internal control, and data were expressed as the relative fold changes in protein levels.

### Cryo-EM sample preparation

The purified BMAL1–HIF2A heterodimer was mixed with a twofold molar excess of the HRE dsDNA, followed by incubation on ice for 20 min. The mixture was then applied onto the Superdex 200 Increase 10/300 GL column. The eluate fractions from the column were analysed by SDS–PAGE and stained with Coomassie blue. The peak fractions corresponding to the BMAL1–HIF2A–DNA complex were combined and concentrated to 0.5 mg ml^−1^ for preparing cryo-EM specimens. In brief, 3 μl of the purified complex was applied onto freshly glow-discharged R 2/1 holey carbon 300 mesh copper grids (C-flat). The grids were then vitrified in liquid ethane using an FEI Vitrobot (Mark IV).

### Cryo-EM data collection and processing

Single-particle cryo-EM images were collected using the FEI Titan Krios (300 KV) electron microscope with a Gatan GIF Quantum K2 direct electron detector. The images were automatically acquired using EPU (Thermo Fisher Scientific, v.2.10) at underfocus values ranging from 1.0 to 2.5 μm with a pixel size of 0.85 Å px^−1^. Each micrograph was exposed for 9 s with a total dose of about 75 e^−^ Å^−2^, which was fractionated into 45 frames. The movie frames were aligned using MotionCor2 (v.1.4.0)^78^, resulting in a total of 18,039 micrographs. Gctf (v.1.18)^79^ was used to estimate the parameters of contrast transfer function (CTF) for each micrograph. crYOLO (v.1.10)^80^ was used for reference-free automatic particle picking, yielding a total of approximately 710,000 particles. The particles were extracted with a box size of 352. Multiple rounds of Cryosparc (v.-2.5)^81^ 2D classification were initially performed to remove junk particles. Next, the remaining particles were subjected to an additional round of 2D classification using RELION (v.3.1)^82^, yielding a stack of 420,000 particles. An ab initio model, showing secondary structural features bound to DNA, was generated using CryoSPARC and then used as a reference for subsequent 3D analysis. Multiple rounds of 3D classification were performed in RELION, resulting in 141,218 particles. The particles were rescaled to a pixel size of 1.02 Å px^−1^ with a box size of 328 pixels and subsequently subjected to 3D refinement. The reference map for 3D refinement was filtered to 25 Å, and a low-pass filtered mask at 25 Å was generated using a threshold that included the protein and DNA regions, followed by a 4-pixel dilation and 5-pixel soft padding. 3D refinement in RELION produced a 4.7 Å-resolution map of the BMAL1–HIF2A–DNA complex. Next, these particles were further processed by CTF refinement and Bayesian polishing in RELION, resulting in 141,218 shiny particles. These particles were then subjected to 3D refinement, producing a map with a resolution of 4.3 Å. Multiple rounds of 3D classification, with and without alignment, were then conducted. A final set of 43,098 shiny particles was used for 3D refinement, resulting in a map with an average resolution of 3.6 Å. The resolution was calculated using a low-pass filtered mask at 15 Å, created with a threshold of 0.008, a 3-pixel dilation and 3-pixel soft padding. The *b*-factor for the RELION map was −61. The final map was sharpened using DeepEMhancer (v.0.14) with the wideTarget training mode^83^. The local resolutions were calculated using CryoSPARC. The resolutions of the 3D maps were estimated using gold-standard Fourier shell correlation (FSC) curves with a 0.143 cut-off criteria^84^. The directional resolution anisotropy was quantified by the 3D FSC algorithm (v.3.0)^85^. The final set of 43,098 particles had an *E*_od_ value of 0.74 with the best and worst PSF resolutions of 2.8 and 5.3 Å, respectively, as calculated by cryoEF (v.1.1.0)^86^, indicating minor anisotropy. The cryo-EM data collection and image analysis procedures are shown in Supplementary Table 11 and Extended Data Fig. 9, respectively.

### Model building and refinement

To build an atomic model of the BMAL1–HIF2A–DNA complex, the crystal structure of HIF2A (Protein Data Bank (PDB): 4ZP4) and a 22-bp B-form DNA duplex were used as templates and fitted into the deepEMhancer map of BMAL1–HIF2A–DNA by rigid-body fitting in Chimera (v.1.15)^87^. Owing to the structural arrangement, the bHLH, PAS-A and PAS-B domains of the BMAL1 crystal structure (PDB: 4F3L) were individually fitted into the map. The model was then manually built and adjusted in Coot (v.1.1)^88^, followed by real-space refinement in Phenix (v.1.21)^89^ (Supplementary Table 11). In the final BMAL1–HIF2A–DNA atomic model, amino acids for HIF2A (3–12, 76–88, 151–163, 174–185, 201–219 and 356–361) and BMAL1 (68–74, 102–110, 126–144, 164–167, 210–242, 254–279, 291–311, 321–337, 345–349, 407–412 and 441–488) as well as a four DNA bases near the terminal ends were not built because of missing or poor densities. The refined model statics are shown in Supplementary Table 11. All molecular graphic figures were generated by Chimera, ChimeraX (v.1.7)^90^ and PyMOL (v.2.5.5)^91^.

### Quantification and statistical analysis

Unless otherwise specified, all results are presented as the mean ± s.e.m., with the precise number of biological replicates (*n*) described in the figure legends. All data were plotted from independent biological replicates. The Shapiro–Wilk normality test was used to assess normal distribution. The equality of variances for unpaired *t*-tests was determined using the *F-*test. Statistical analysis was performed using GraphPad Prism 10.0, using unpaired *t*-tests (two-tailed), Welch’s *t-*tests (for unequal variances), Mann–Whitney *U*-tests (when normality assumptions were not met) or one-way ANOVA with Bonferroni’s multiple-comparison analysis, depending on the dataset. Outliers were identified using the ROUT method (*Q* = 1%) in GraphPad Prism. For ChIP–qPCR data analysis in Fig. 3s, values identified as outliers were excluded from the statistical analysis. Cardiac function assessment in *Bmal1*^*loxP/loxP*^ myosin-Cre^+^, *Hif2a*^*loxP/loxP*^ myosin-Cre^+^ and *Hif1a*^*loxP/loxP*^ myosin-Cre^+^ mice was compared to a shared myosin-Cre^+^ control group. Cardiac function data from *Bmal1*^*loxP/loxP*^ myosin-Cre^+^ mice were used as the control for comparing NOB-treated *Bmal1*^*loxP/loxP*^ myosin-Cre^+^ mice. Similarly, cardiac function data from *Areg*^*−/−*^ mice were used as the control for comparing NOB-treated *Areg*^*−/−*^ mice. Cardiac function data from C57BL/6J mice were partially used as the control group for *Areg*^*−/−*^ mice. This strategy minimized animal use and ensured a consistent baseline, optimizing research efficiency while enabling reliable comparative analysis.

### Reporting summary

Further information on research design is available in the Nature Portfolio Reporting Summary linked to this article.

## Online content

Any methods, additional references, Nature Portfolio reporting summaries, source data, extended data, supplementary information, acknowledgements, peer review information; details of author contributions and competing interests; and statements of data and code availability are available at 10.1038/s41586-025-08898-z.

## Supplementary information

Supplementary Information Supplementary Notes 1–7.

Reporting Summary

Supplementary Tables Supplementary Tables 1–9.

Supplementary Data Source data for Supplementary Tables 1–9.

Supplementary Video 1 Structural rearrangement of BMAL1 after binding with various partners.

## Source data

Source Data Fig. 1

Source Data Fig. 2

Source Data Fig. 3

Source Data Fig. 4

Source Data Fig. 5

Source Data Extended Data Fig. 3

Source Data Extended Data Fig. 4

Source Data Extended Data Fig. 5

Source Data Extended Data Fig. 6

Source Data Extended Data Fig. 7

Source Data Extended Data Fig. 8

## Data Availability

The mouse myocardial IRI heart bulk RNA-seq data are available at the NCBI GEO database under accession number GSE255307. Analyses of the mouse RNA-seq data were performed using the *Mus musculus* reference genome assembly GRCm38 (Genome Reference Consortium Mouse Build 38), which is available at the NCBI under accession number GCF_000001635.20. Human surgical LV bulk RNA-seq data are available under controlled access through the NIH database of Genotypes and Phenotypes (dbGaP) under accession number phs001679.v1.p1. Access to these data is restricted due to privacy and ethical considerations. Requests must be submitted through dbGaP’s data access request process. Interested researchers should apply for access through dbGaP by contacting the NIH Data Access Committee (DAC) and providing a detailed research proposal outlining the intended use of the data. The DAC typically reviews requests within 2 weeks, and access is granted subject to compliance with data use agreements. For further details on controlled access policies, data use agreements and any additional restrictions, please refer to the dbGaP study page linked above. Questions regarding data access can be directed to the dbGaP helpdesk. The microarray assay for gene expression transcript levels in post-ischaemic myocardium from myosin-Cre^+^ or *Hif2a*^*loxP*/*loxP*^ myosin-Cre^+^ mice was re-analysed using data obtained from the GEO (GSE67308). The cryo-EM map of the BMAL1–HIF2A–DNA complex was deposited at the Electron Microscopy Data Bank under accession number EMD-43237. The corresponding atomic model was deposited at the RCSB PDB under accession number 8VHG. Source data are provided with this paper.
