# Supplementary material for: BMAL1–HIF2A heterodimer modulates circadian variations of myocardial injury
Source: Nature. 2025 Apr 23;641(8064):1017–28. doi: 10.1038/s41586-025-08898-z (PMC12095075; doi:10.1038/s41586-025-08898-z)
Supplement: Supplementary file 1 — Supplementary Notes 1–7. [file 41586_2025_8898_MOESM1_ESM.pdf]

nature portfolio

https://doi.org/10.1038/s41586-025-08898-z

Supplementary information

BMAL1–HIF2A heterodimer modulates circadian variations of myocardial injury

In the format provided by the authors and unedited

BMAL1/HIF2A heterodimer modulates circadian variations of myocardial injury

Wei Ruan[1,2,18*], Tao Li[3,18], In Hyuk Bang[1,18], Jaewoong Lee[4,18], Wankun Deng[5], Xinxin Ma[1], Cong Luo[1,2], Fang Du[1,6], Seung-Hee Yoo[3], Boyun Kim[1,7], Jiwen Li[1,8], Xiaoyi Yuan[1], Katherine Figarella[1], Yu A An[1], Yin-Ying Wang[5], Yafen Liang[1,17], Matthew DeBerge[1], Dongze Zhang[1], Zhen Zhou[9], Yanyu Wang[1], Joshua M. Gorham[10], Jonathan G. Seidman[10], Christine E. Seidman[10], Sary F. Aranki[11], Ragini Nair[1], Lei Li[12], Jagat Narula[13], Zhongming Zhao[5], Alemayehu A Gorfe[14], Jochen D. Muehlschlegel[15], Kuang-Lei Tsai[3,16*], and Holger K. Eltzschig[1,17*]

[1]Department of Anesthesiology, Critical Care and Pain Medicine, The University of Texas Health Science Center at Houston, McGovern Medical School, Houston, TX, 77030, USA. [2]Department of Anesthesiology, Second Xiangya Hospital, Central South University, Changsha, 410011, China. [3]Department of Biochemistry and Molecular Biology, The University of Texas Health Science Center at Houston, McGovern Medical School, Houston, TX, 77030, USA. [4]Department of Anesthesiology, Yale University School of Medicine, New Haven, CT, 06510, USA. [5]Center for Precision Health, McWilliams School of Biomedical Informatics, The University of Texas Health Science Center at Houston, Houston, TX, 77030, USA. [6]Department of Anesthesiology, Zhongshan Hospital, Fudan University, Shanghai, 200032, China. [7]Major in Aquaculture and Applied Life Sciences, College of Fisheries Science, Pukyong National University, Busan, 48513, Republic of Korea. [8]Department of Cardiac Surgery, Sir Run Run Shaw Hospital, School of Medicine, Zhejiang University, Hangzhou, 310016, China. [9]Division of Medical Genetics, Department of Internal Medicine, The University of Texas Health Science Center at Houston, McGovern Medical School, Houston, TX, 77030, USA. [10]Department of Genetics, Harvard Medical School, Boston, MA, 02115, USA. [11]Department of Surgery, Division of Cardiac Surgery, Brigham and Women's Hospital, Harvard Medical School, Boston, MA, 02115, USA. [12]Institute of Systems and Physical Biology, Shenzhen Bay Laboratory, Shenzhen, 518055, China. [13]Division of Cardiology, Department of Medicine, The University of Texas Health Science Center at Houston, McGovern Medical School, Memorial Hermann Hospital, Houston, TX, 77030, USA. [14]Department of Integrative Biology and Pharmacology, The University of Texas Health Science Center at Houston, McGovern Medical School, Houston, TX, 77030, USA. [15]Department of Anesthesiology and Critical Care Medicine, Johns Hopkins University School of Medicine, Baltimore, MD, 21287, USA. [16]MD Anderson Cancer Center, UTHealth Houston Graduate School of Biomedical Sciences, Houston, TX, USA. [17]Center for Outcomes Research, UTHealth Houston, Houston, TX, USA. [18]These authors contributed equally.

[*]Corresponding authors:
Holger K. Eltzschig, MD, PhD. Email: holger.eltzschig@uth.tmc.edu
or Kuang-Lei Tsai, PhD. Email: kuang-lei.tsai@uth.tmc.edu
or Wei Ruan, MD, PhD. Email: Wei.Ruan@uth.tmc.edu

Contents

Supplementary Note 1: Supplementary Figures (1-10) 3
Supplementary Note 2: Supplementary Tables (1-12) 19
Supplementary Note 3: Supplementary Video (1) 26
Supplementary Note 4: Supplementary Discussion 27
Supplementary Note 5: Uncropped gel blot images for main Figures 41
Supplementary Note 6: Uncropped gel blot images for Extended Data Figures 49
Supplementary Note 7: Uncropped gel blot images for Supplementary Figures 59

Supplementary Note 1: Supplementary Figures

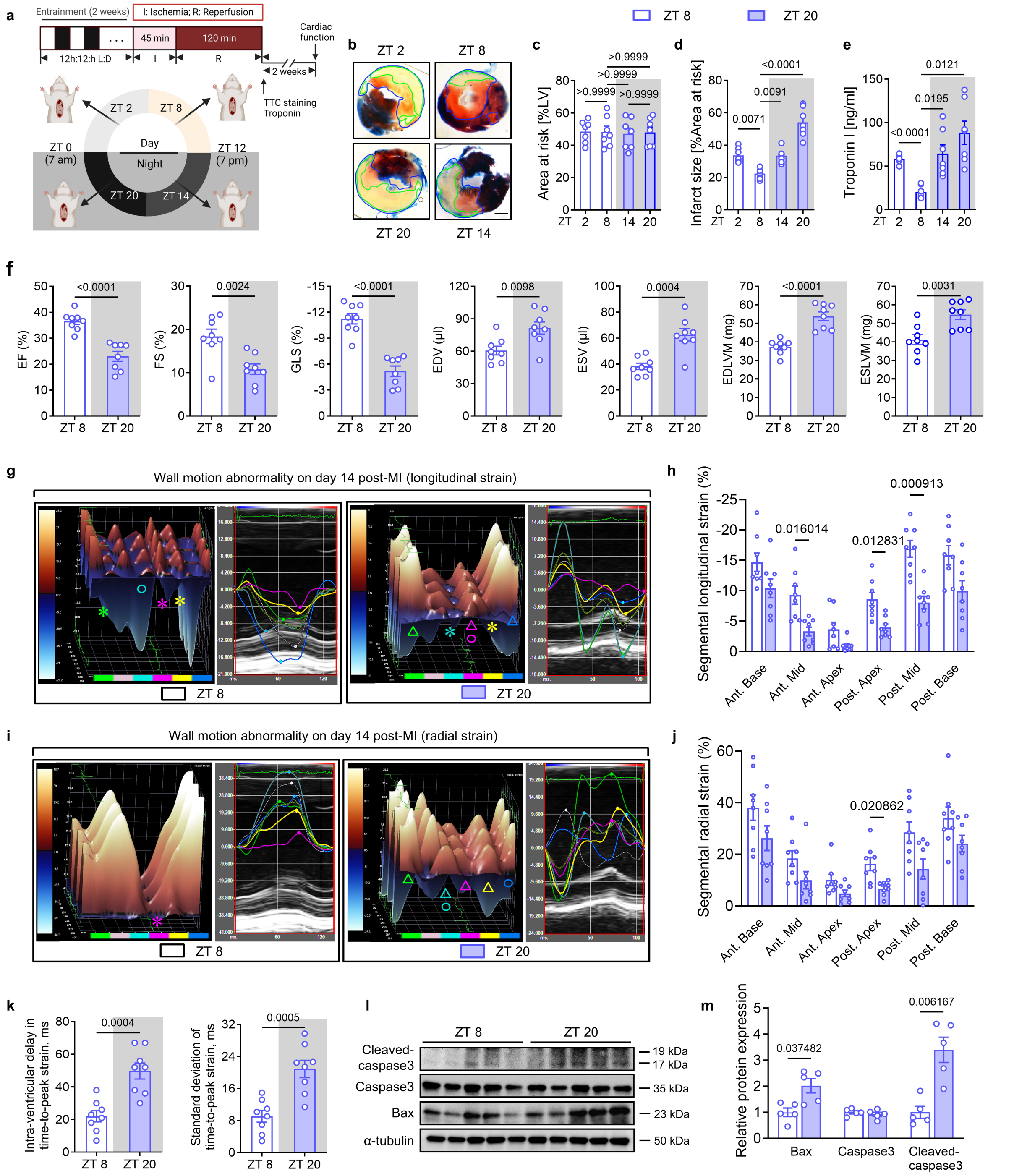

Fig. S1. Circadian variations of myocardial injury in C57BL/6J mice.
a, Experimental setup for evaluating myocardial injury and cardiac function in C57BL/6J mice subjected to myocardial ischemia and reperfusion injury (IRI) at different Zeitgeber times (ZT2, ZT8, ZT14, and ZT20). **b-e, b,** Heart slices stained with Evan's blue and TTC following 2h of reperfusion: infarct area (green line) and area at risk (AAR; blue line); scale bar, 1 mm. **c,** Percentage of AAR relative to left ventricular (LV) size. **d,** Infarct size as percentage of AAR. **e,** Serum troponin I levels. b-e: n = 7 mice/group/time point; one-way ANOVA. **f-k,** Cardiac function measured by speckle-tracking echocardiography (STE) on day 14 post-MI. **f,** LV

systolic function in ejection fraction (EF), fractional shortening (FS), and global longitudinal strain (GLS); left ventricular end-diastolic volume (EDV) and end-systolic volume (ESV); end-diastolic left ventricular mass (EDLVM) and end-systolic left ventricular mass (ESLVM); unpaired two-tailed *t*-tests. **g,** LV 3D (left) and six-segment longitudinal strain images (right) demonstrating wall motion abnormalities. Color-coded segments: Anterior Base (dark blue), Anterior Mid (yellow), Anterior Apex (magenta), Posterior Apex (cyan), Posterior Mid (light pink), and Posterior Base (green). Symbols indicate reduced contractility (stars), dyskinesis (triangles), and dyssynchrony (circles). **h,** LV segmental wall contractility detected by peak longitudinal strain; unpaired two-tailed *t*-tests. **i,** LV 3D (left) and 6-segment long-axis radial strain images (right) demonstrating wall motion abnormalities. **j,** LV segmental wall contractility detected by peak radial strain; unpaired two-tailed *t*-tests. **k,** LV mechanical dyssynchrony measured by intra-ventricular delay and standard deviation of time-to-peak strain based on peak longitudinal strain; unpaired two-tailed *t*-tests. f-k, n = 8 mice/group/time point. **l,** Protein levels of cleaved-caspase3, caspase3, Bax, and α-tubulin were measured by Western blot analysis in the AAR on day 3 post-MI. **m,** Quantification of protein levels in **(l)**. n = 5 mice/group/time point; unpaired two-tailed *t*-tests. All data are mean ± s.e.m.

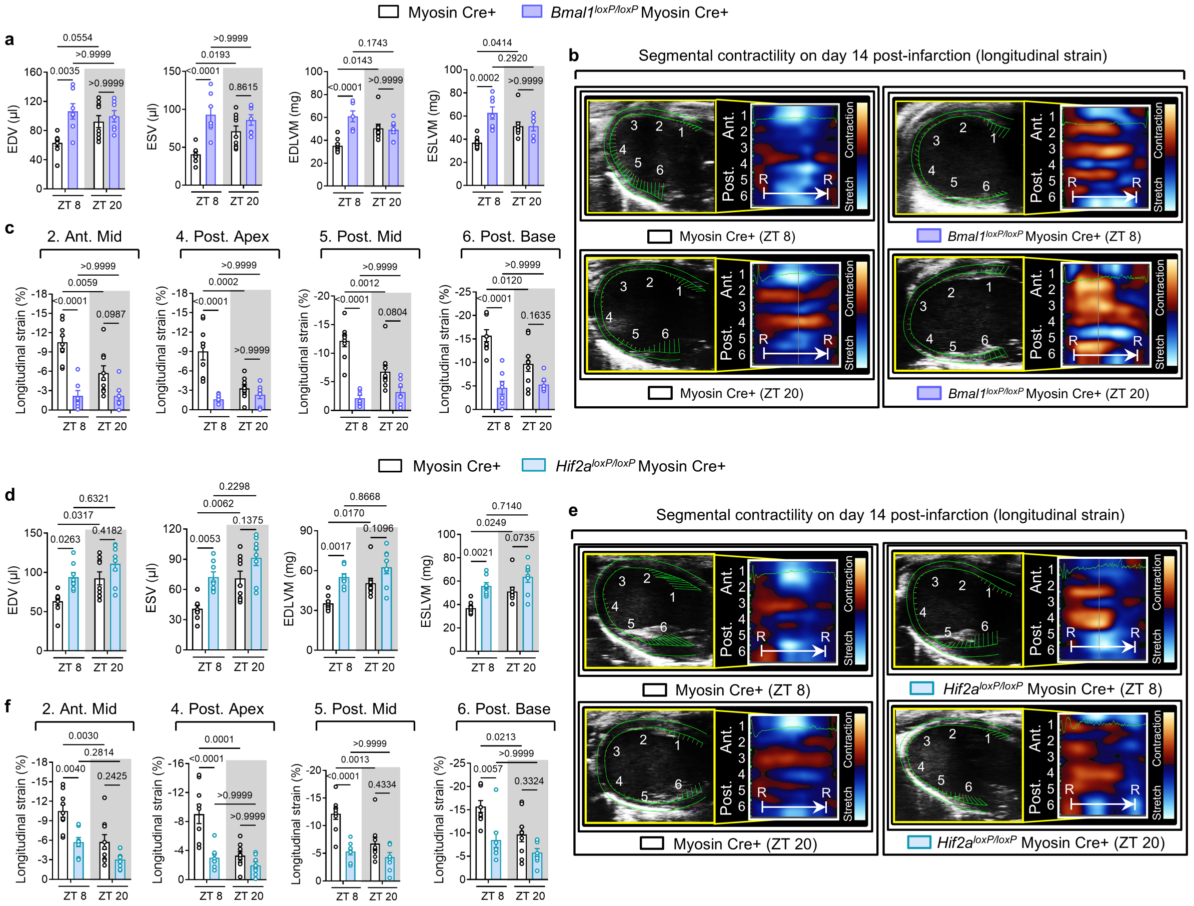

Fig. S2. Cardiomyocyte-specific *Bmal1* or *Hif2a* deletion diminishes the circadian variations of cardiac function impairment (see also **Fig. 1o-q, 2p-s**).

a-c, Cardiac function was evaluated by STE on day 14 post-MI in *Bmal1*$^{loxP/loxP}$ Myosin Cre+ mice and Myosin Cre+ mice subjected to IRI at ZT8 or ZT20. **a,** LV volume (EDV and ESV) and mass (EDLVM and ESLVM). **b,** B-mode images from the LV long-axis view with 2D longitudinal strain analysis showing 6 segments (1. Anterior Base; 2. Anterior Middle; 3. Anterior Apex; 4. Posterior Apex; 5. Posterior Middle; 6. Posterior Base). **c,** LV segmental wall contractility detected by peak longitudinal strain. a-c. n = 7/*Bmal1*$^{loxP/loxP}$ Myosin Cre+ mice/time point and n = 9 for Myosin Cre+ mice/time point. **d-f**, Cardiac function was evaluated by STE on day 14 post-MI in *Hif2a*$^{loxP/loxP}$ Myosin Cre+ mice and Myosin Cre+ mice subjected to IRI at ZT8 or ZT20. **d,** LV volume (EDV and ESV) and mass (EDLVM and ESLVM). **e,** Representative B-mode images from the LV long-axis view with 2D longitudinal strain analysis showing 6 segments. **f,** LV segmental wall contractility detected by peak longitudinal strain. n = 8/*Hif2a*$^{loxP/loxP}$ Myosin Cre+ mice/time point, n = 9/Myosin Cre+ mice/time point. Cardiac function assessment in *Bmal1*$^{loxP/loxP}$ Myosin Cre+ and *Hif2a*$^{loxP/loxP}$ Myosin Cre+ were compared to a shared Myosin Cre+ control group to minimize the number of animals used, ensuring ethical standards are met while maintaining experimental rigor. All data are mean ± s.e.m. Statistical analysis was performed using two-way ANOVA.

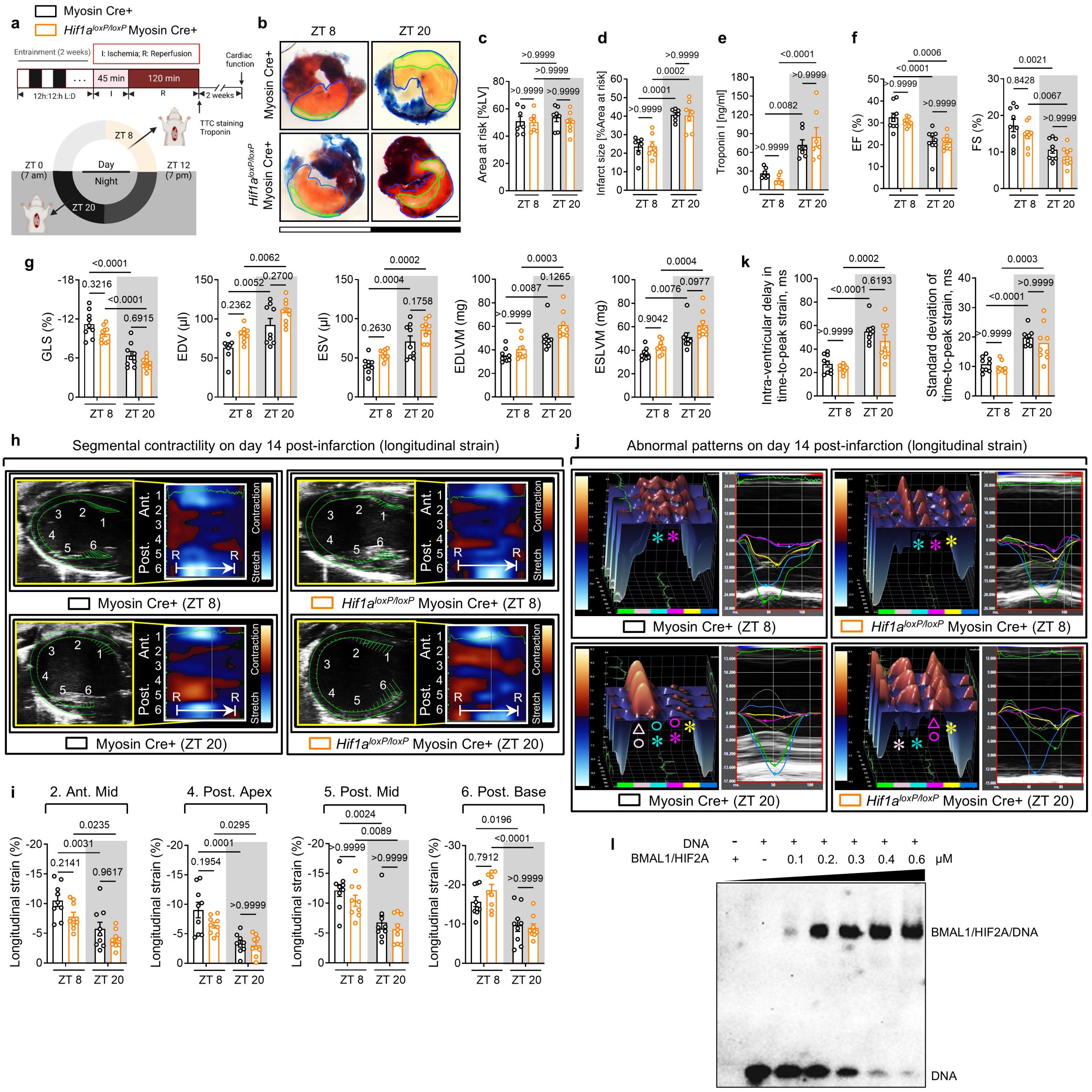

Fig. S3. Cardiomyocyte-specific *Hif1a* deletion does not influence circadian-dependent myocardial injury.

a, Experimental setup for evaluating myocardial injury and cardiac function in *Hif1a*$^{loxP/loxP}$ Myosin Cre+ mice and Myosin Cre+ mice subjected to IRI at ZT8 or ZT20. **b-e, b,** Heart slices subjected to Evan's blue and TTC double staining following 2h of reperfusion: infarct area (green line) and AAR (blue line); scale bar, 1 mm. Quantification of AAR as a percentage of LV **(c)**, infarct size as a percentage of AAR **(d)**, and serum troponin I levels **(e)** after 2h of reperfusion. n = 7 mice/group/time point. Statistical analysis was performed using two-way ANOVA. **f-k,** Cardiac function assessed on day 14 post-MI by STE. **f,** EF and FS. **g,** GLS, EDV, ESV, EDLVM, and ESLVM. **h,** Representative B-mode images from LV long-axis view with 2D longitudinal strain analysis, showing 6 segments (1. Anterior Base; 2. Anterior Middle; 3. Anterior Apex; 4. Posterior Apex; 5. Posterior Middle; 6. Posterior Base). **i,** LV segmental wall contractility detected by peak longitudinal strain. **j,** Representative LV 3D (left) and six-segment longitudinal strain images (right), demonstrating wall motion abnormalities. Color-

coded segments: Anterior Base (dark blue), Anterior Mid (yellow), Anterior Apex (magenta), Posterior Apex (cyan), Posterior Mid (light pink), and Posterior Base (green). Symbols indicate specific wall motion abnormalities: stars for reduced contractility, triangles for dyskinesis, and circles for dyssynchrony. **k,** LV mechanical dyssynchrony as measured by intra-ventricular delay in time-to-peak strain and standard deviation of time-to-peak strain based on peak longitudinal strain. f-k: n = 9 mice/group/time point. Cardiac function assessment in *Bmal1*$^{loxP/loxP}$ Myosin Cre+, *Hif2a*$^{loxP/loxP}$ Myosin Cre+, and *Hif1a*$^{loxP/loxP}$ Myosin Cre+ mice were compared to a shared Myosin Cre+ control group to minimize the number of animals used. Statistical analysis was performed using two-way ANOVA. All data are mean ± s.e.m. **l,** The electrophoretic mobility shift assay (EMSA) was used to investigate the binding of the BMAL1/HIF2A heterodimer to the biotinylated-HRE dsDNA. Five different protein concentrations were used, as specified. The 22 bp biotin end-labeled DNA duplex was detected using HRP-conjugated streptavidin with chemiluminescent substrates.

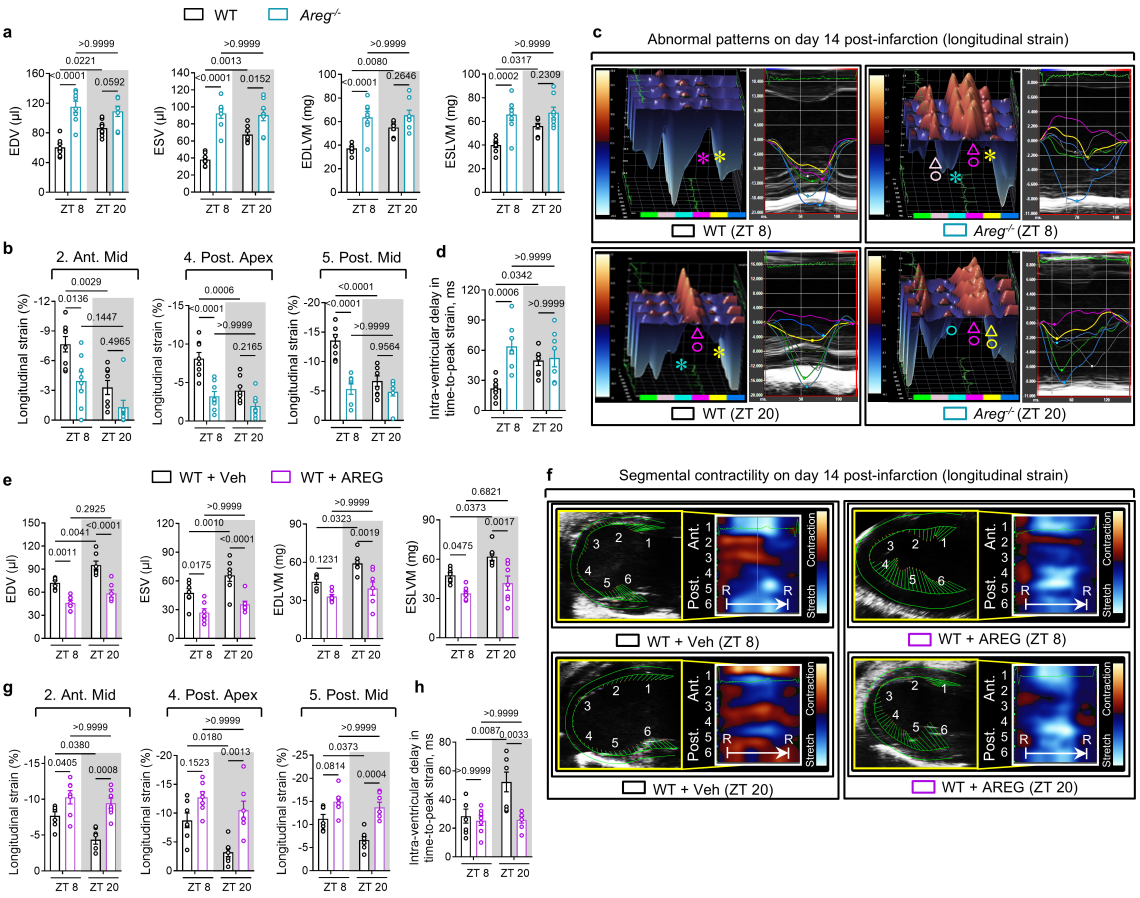

Fig. S4. ***Areg*** **deletion dampens cardiac function at ZT8, while AREG treatment at ZT20 improves it** (see also **Fig. 4f, g, q, r**).

a-d, Cardiac function was measured by STE on day 14 post-MI in *Areg*$^{-/-}$ and wild-type (WT) mice subjected to IRI at ZT8 or ZT20. **a,** EDV, ESV, EDLVM, and ESLVM. **b,** LV segmental wall contractility detected by peak longitudinal strain. **c,** LV 3D (left) and six-segment longitudinal strain images (right) demonstrating wall motion abnormalities. Color-coded segments: Anterior Base (dark blue), Anterior Mid (yellow), Anterior Apex (magenta), Posterior Apex (cyan), Posterior Mid (light pink), and Posterior Base (green). Symbols indicate specific wall motion abnormalities: stars for reduced contractility, triangles for dyskinesis, and circles for dyssynchrony. **d,** LV mechanical dyssynchrony as measured by intra-ventricular delay of time-to-peak strain based on longitudinal strain. n = 8/animals/group/time point. Cardiac function data from C57BL/6 mice were partially used as the control group for *Areg*$^{-/-}$ mice. **e-h,** To mimic the clinical scenario where treatment begins after myocardial infarction onset, AREG (10 μg) or vehicle (Veh) was administered at the start of reperfusion and subsequently given daily post-MI at either ZT8 or ZT20. Cardiac function was evaluated on day 14 post-MI by STE. **e,** EDV, ESV, EDLVM and ESLVM. **f,** Representative B-mode images from the LV long-axis view with 2D longitudinal strain analysis showing 6 segments (1. Anterior Base; 2. Anterior Middle; 3. Anterior Apex; 4. Posterior Apex; 5. Posterior Middle; 6. Posterior Base). **g,** LV segmental wall contractility detected by peak longitudinal strain. **h,** LV mechanical dyssynchrony measured by intra-ventricular delay in time-to-peak strain based on longitudinal strain. n = 7 mice/group/time point. Data are mean ± s.e.m. Statistical analysis was all performed using two-way ANOVA.

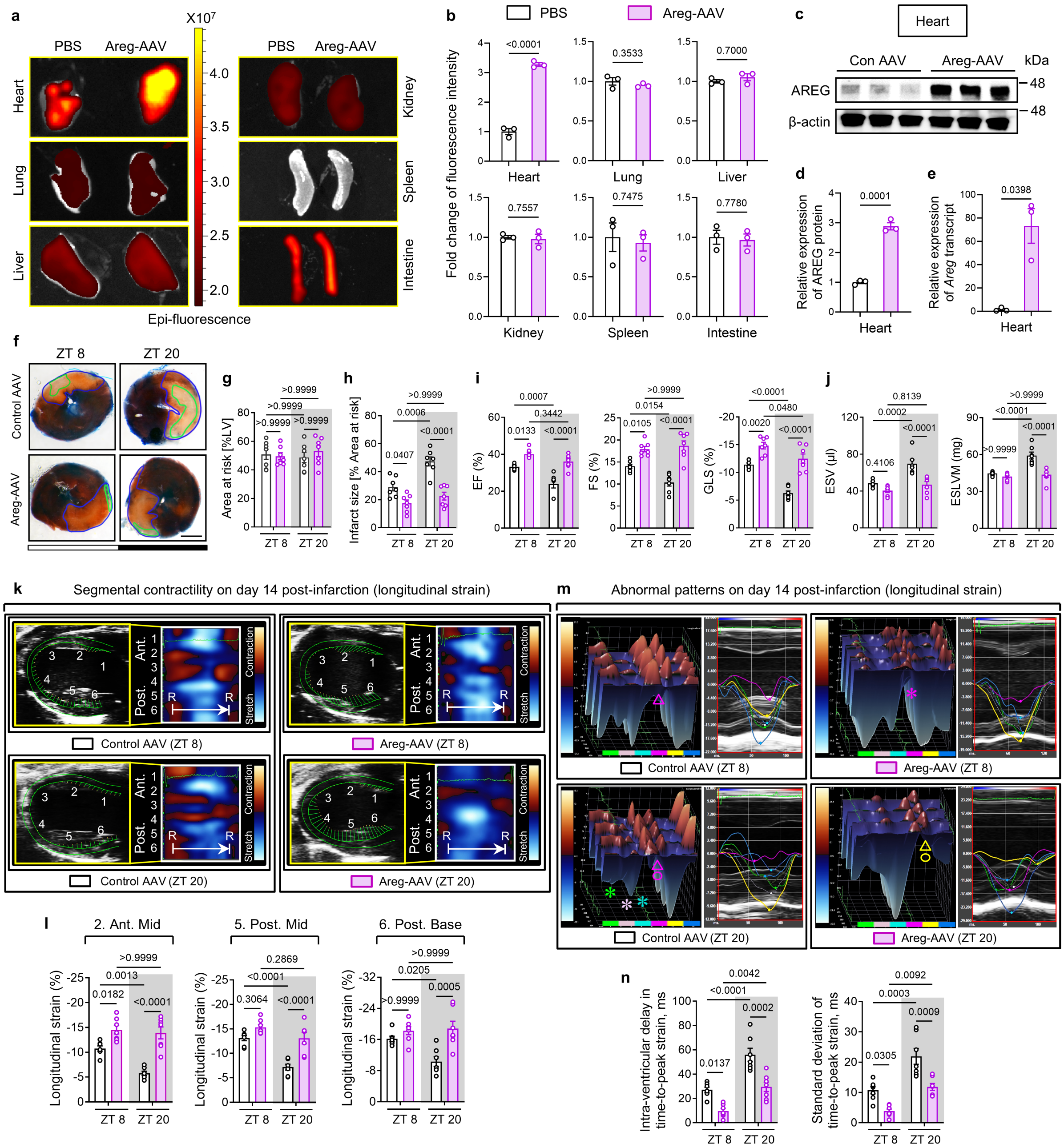

Fig. S5. Genetic overexpression of *Areg* provides circadian-dependent cardioprotection. **a** and **b**, *Ex vivo* eGFP fluorescence signals were measured in the heart, lung, liver, kidney, spleen, and intestine of C57BL/6J mice on day 28 following Areg-AAV administration (2 × 10^{11} GC per mouse). **a,** Representative eGFP fluorescence images. **b,** Quantification of fluorescence intensity in (**a**). n = 3 mice/group; The Mann-Whitney test was used to evaluate fluorescence intensity in the livers, while unpaired two-tailed *t*-tests were applied to the remaining panels. **c-e,** AREG expression in the hearts of mice on day 28 post-Areg-AAV or control-AAV (Con AAV) administration. **c,** Representative Western blot analysis. **d,** Quantification of AREG protein levels in (**c**); unpaired two-tailed *t*-tests. **e,** Real-time PCR analysis of *Areg* transcript levels; Welch's *t*-tests. c-e: n = 3 mice/group. **f-h,** C57BL/6J mice were subjected to myocardial IRI at ZT8 or ZT20 on day 28 following Areg-AAV or control AAV administration. **f,** Evan's blue/TTC staining on day 1 post-MI showing infarct area (green

line) and AAR (blue line); scale bar, 1 mm. **g,** Percentage of AAR relative to LV size. **h,** Infarct size as percentage of AAR. f-h: n = 7 mice/group/time point; two-way ANOVA. **i-n,** Cardiac function by STE on day 14 post-MI. **i,** EF, FS, and GLS. **j,** ESV and ESLVM. **k,** B-mode LV long-axis images with 2D longitudinal strain analysis showing 6 segments (1. Anterior Base; 2. Anterior Middle; 3. Anterior Apex; 4. Posterior Apex; 5. Posterior Middle; 6. Posterior Base). **l,** LV segmental wall contractility. **m,** LV 3D (left) and six-segment longitudinal strain images demonstrating wall motion abnormalities (stars: reduced contractility; triangles: dyskinesis; circles: dyssynchrony). Color-coded segments: Anterior Base (dark blue), Anterior Mid (yellow), Anterior Apex (magenta), Posterior Apex (cyan), Posterior Mid (light pink), and Posterior Base (green). **n,** LV mechanical dyssynchrony. i-n: n = 7 mice/group/time point; two-way ANOVA. All data are mean ± s.e.m.

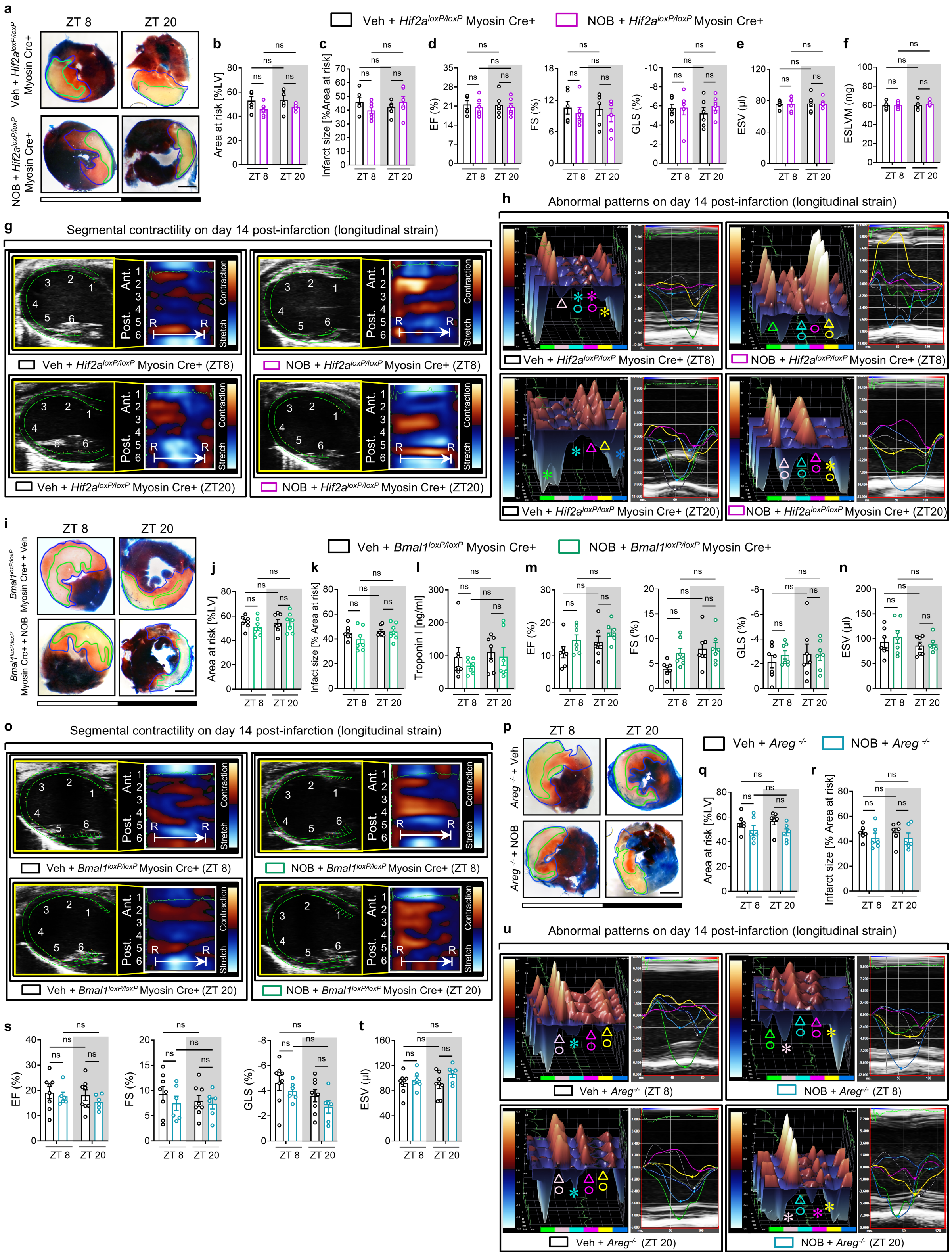

Fig. S6. NOB failed to alleviate myocardial injury in *Hif2a*-, *Bmal1*-, and *Areg*-deficient mice.

a-h, NOB-treated (200 mg/kg, i.p., every other day) or Veh-treated *Hif2a*$^{loxP/loxP}$ Myosin Cre+ mice were subjected to myocardial IRI at ZT8 or ZT20. **a-c, a,** Representative heart slices subjected to Evan's blue and TTC double staining on day 1 post-MI: infarct area (green line)

and AAR (blue line); scale bar, 1 mm. **b,** Percentage of AAR relative to LV. **c,** Infarct size represented as percentage of AAR. a-c: n = 6 mice/group/time point. **d-h,** Cardiac function was evaluated by STE on day 14 post-MI. **d,** EF, FS, and GLS. **e,** ESV. **f,** ESLVM. **g,** B-mode images from LV long-axis view with 2D longitudinal strain analysis showing 6 segments. **h,** LV 3D (left) and six-segment longitudinal strain images demonstrating wall motion abnormalities. d-h: n = 6 mice/group/time point. **i-o**, NOB-treated or Veh-treated *Bmal1*$^{loxP/loxP}$ Myosin Cre+ mice were subjected to myocardial IRI at ZT8 or ZT20. **i-l, i,** Representative heart slices subjected to Evan's blue and TTC double staining after 2 h of reperfusion: infarct area (green line) and AAR (blue line); scale bar, 1 mm. **j,** Percentage of AAR relative to LV. **k,** Infarct size represented as percentage of AAR. **l,** Serum troponin I levels. i-l: n = 7 mice/group/time point. **m-o,** Cardiac function was evaluated by STE on day 14 post-MI. **m,** EF, FS, and GLS. **n,** ESV. **o,** B-mode images from LV long-axis view with 2D longitudinal strain analysis showing 6 segments. m-o: n = 7 mice/group/time point. **p-u,** NOB-treated or Veh-treated *Areg*$^{-/-}$ mice were subjected to myocardial IRI at ZT8 or ZT20. **p-r, p,** Evan's blue and TTC double staining of heart slices after 2h of reperfusion: infarct area (green line) and AAR (blue line); scale bar, 1 mm. **q,** Percentage of AAR relative to LV size. **r,** Infarct size represented as percentage of AAR. p-r: n = 6 animals/group/time point. **s-u** Cardiac function was evaluated by STE on day 14 post-MI. **s,** EF, FS, and GLS. **t,** ESV. **u,** LV 3D (left) and six-segment longitudinal strain images (right) demonstrating wall motion abnormalities. s-u: n = 8/Veh-treated *Areg*$^{-/-}$/time point and n = 6/NOB-treated *Areg*$^{-/-}$/time point. Cardiac function data from *Bmal1*$^{loxP/loxP}$ Myosin Cre+ mice were used as controls for comparing NOB-treated *Bmal1*$^{loxP/loxP}$ Myosin Cre+ mice. Similarly, cardiac function data from *Areg*$^{-/-}$ mice were served as controls for comparing NOB-treated *Areg*$^{-/-}$ mice. Data are mean ± s.e.m. Statistical analysis was all performed using two-way ANOVA. ns: not significant.

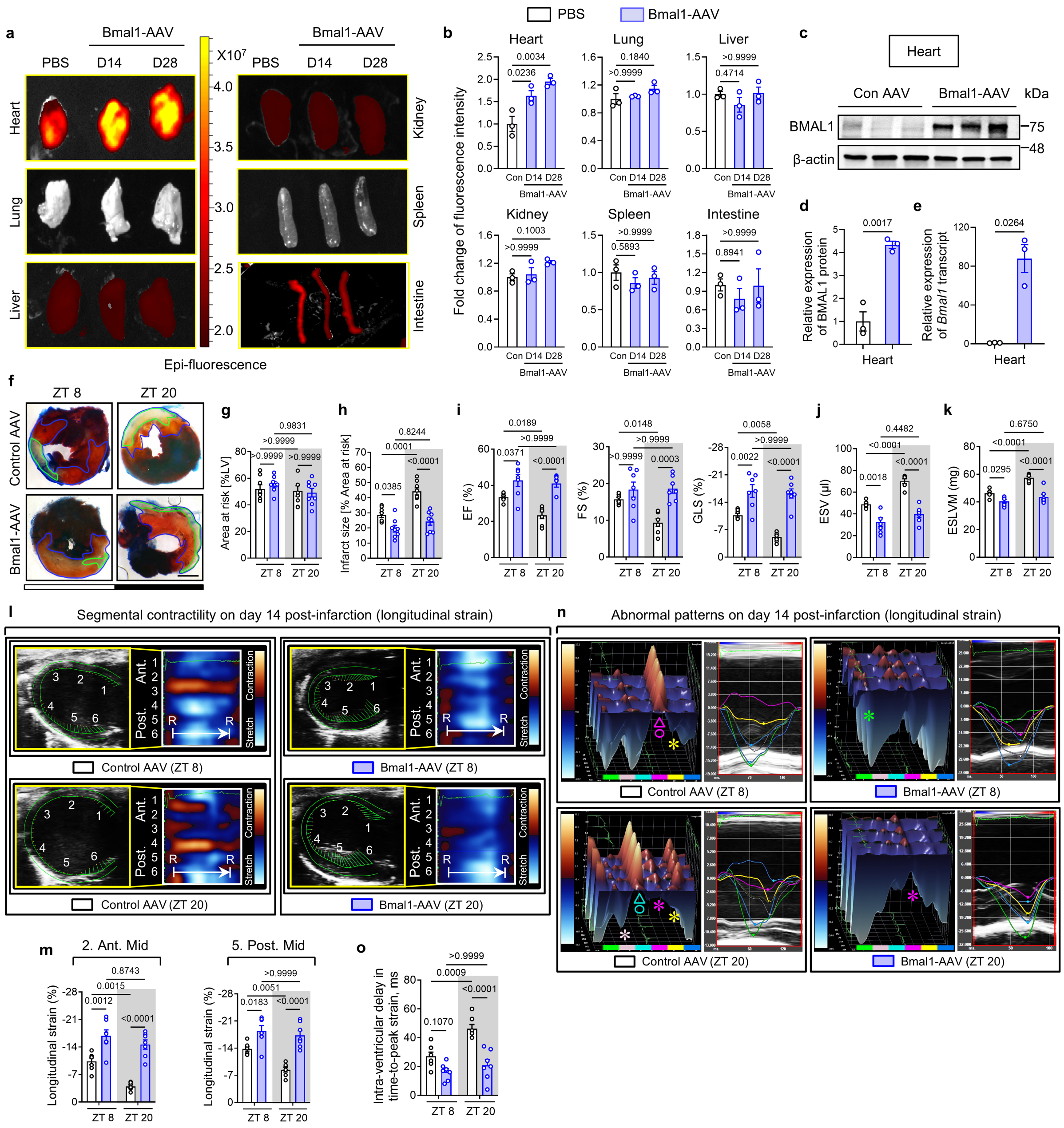

Fig. S7. Genetic overexpression of *Bmal1* provides circadian-dependent cardioprotection. **a** and **b**, *Ex vivo* eGFP fluorescence was measured in various organs (heart, lung, liver, kidney, spleen, and intestine) of C57BL/6J mice on days 14 and 28 following Bmal1-AAV administration (2 × 10^{11} GC per mouse). **a,** Representative fluorescence images. **b,** Quantification of the fluorescence intensity in (**a**). n = 3 mice/group; one-way ANOVA. **c-e,** BMAL1 expression in mouse hearts on day 28 following Bmal1-AAV or Con AAV administration. **c,** Western blot analysis. Samples in (**c**) were also used in **Extended Data Fig. 8g**, sharing the β-actin blot. **d,** Quantification of BMAL1 protein levels in (**c**). n = 3 mice/group; unpaired two-tailed *t*-tests. **e,** Real-time PCR analysis of *Bmal1* transcript levels. n = 3 mice/group; Welch's *t*-tests. **f-h,** These mice were subjected to myocardial IRI at ZT8 or ZT20. **f,** Evan's blue/TTC staining on day 1 post-MI, showing infarct area (green) and AAR (blue); scale bar, 1 mm. **g,** Percentage of AAR relative to LV size. **h,** Infarct size as percentage of AAR. f-h: n = 8 mice/group/time point; two-way ANOVA. **i-o,** Cardiac function evaluated by STE on

day 14 post-MI. **i,** EF, FS, and GLS. **j,** ESV. **k,** ESLVM. **l,** B-mode images from LV long-axis view with 2D longitudinal strain analysis showing 6 segments (1. Anterior Base; 2. Anterior Middle; 3. Anterior Apex; 4. Posterior Apex; 5. Posterior Middle; 6. Posterior Base). **m,** LV segmental wall contractility. **n,** LV 3D (left) and six-segment longitudinal strain images (right) demonstrating wall motion abnormalities (stars: reduced contractility; triangles: dyskinesis; circles: dyssynchrony). Color-coded segments: Anterior Base (dark blue), Anterior Mid (yellow), Anterior Apex (magenta), Posterior Apex (cyan), Posterior Mid (light pink), and Posterior Base (green). **o,** LV mechanical dyssynchrony. i-o: n = 7 mice/group/time point; two-way ANOVA. All data are mean ± s.e.m.

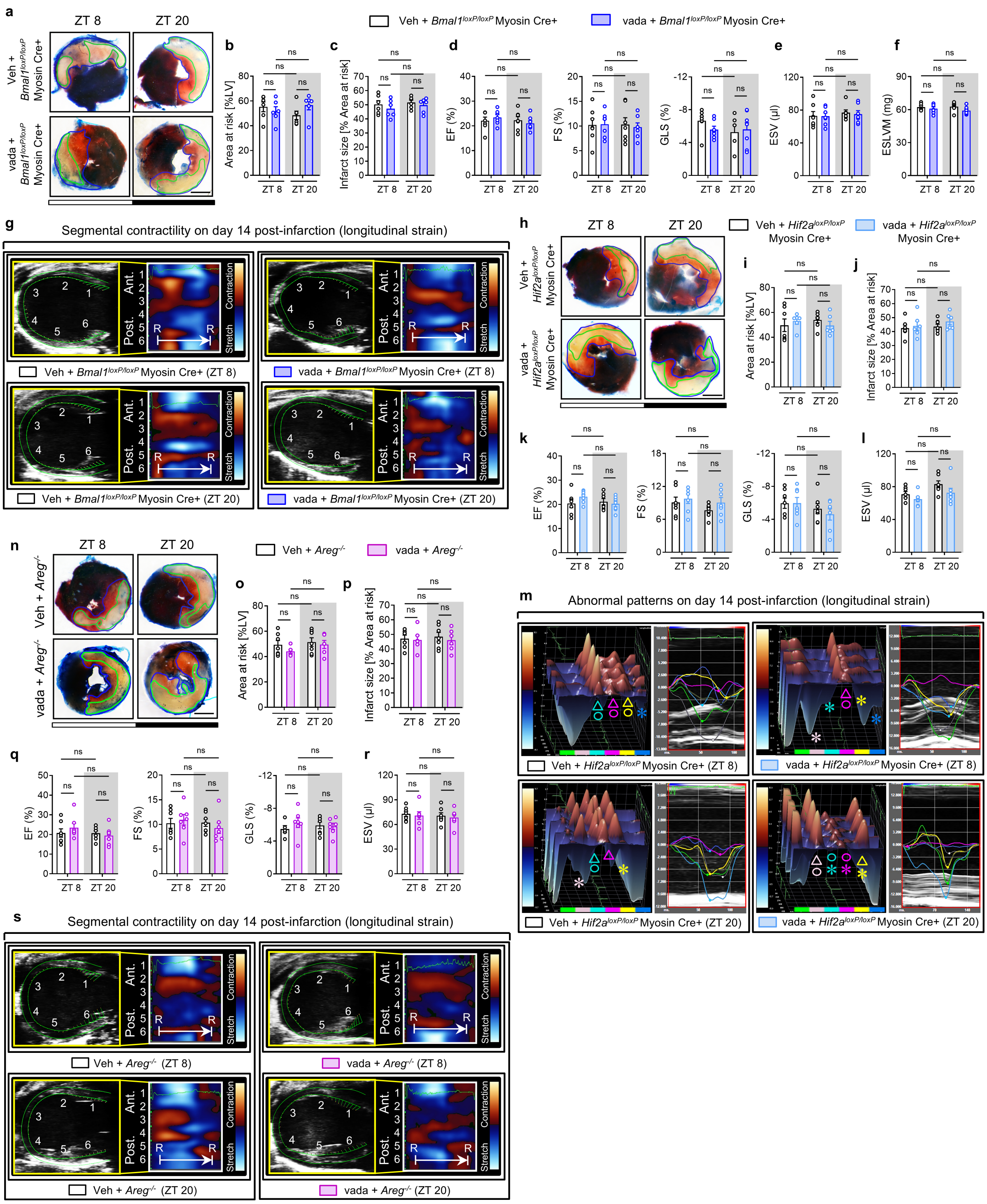

Fig. S8. Vadadustat treatment fails to mitigate myocardial injury in *Hif2a*-, *Bmal1*-, and *Areg*-deficient mice.

a-g, *Bmal1*$^{loxP/loxP}$ Myosin Cre+ mice were treated with vadadustat (vada, 50 mg/kg, i.p., daily) or vehicle control for three consecutive days at ZT8 or ZT20, followed by myocardial IRI at ZT8 or ZT20. **a,** Representative Evan's blue/TTC-stained heart slices on day 1 post-MI: infarct area (green line) and AAR (blue line); scale bar, 1 mm. **b,** Percentage of AAR relative to LV size. **c,** Infarct size as percentage of AAR. a-c: n = 6 mice/group/time point. **d-g,** Cardiac function in *Bmal1*$^{loxP/loxP}$ Myosin Cre+ on day 14 post-MI. **d,** EF, FS, and GLS. **e,** ESV. **f,**

ESLVM. **g,** B-mode LV long-axis images with 2D longitudinal strain analysis showing 6 segments (1. Anterior Base; 2. Anterior Middle; 3. Anterior Apex; 4. Posterior Apex; 5. Posterior Middle; 6. Posterior Base). d-g: n = 7 mice/group/time point. **h-m,** *Hif2a*$^{loxP/loxP}$ Myosin Cre+ mice treated as in **a-g. h,** Evan's blue/TTC-stained heart slices; scale bar, 1 mm. **i,** Percentage of AAR relative to LV size. **j,** Infarct size as percentage of AAR. h-j: n = 7 mice/Veh-treated/ZT8, n = 7 mice/Veh-treated/ZT20, n = 6 mice/vada-treated/ZT8, and n = 6 mice/vada-treated/ZT20. **k-m,** Cardiac function in *Hif2a*$^{loxP/loxP}$ Myosin Cre+ mice on day 14 post-MI. **k,** EF, FS, and GLS. **l,** ESV. **m,** LV 3D and six-segment strain images. Symbols indicate wall motion abnormalities: stars (reduced contractility), triangles (dyskinesis), and circles (dyssynchrony). Color-coded segments: Anterior Base (dark blue), Anterior Mid (yellow), Anterior Apex (magenta), Posterior Apex (cyan), Posterior Mid (light pink), and Posterior Base (green). k-m: n = 7 mice/group/time point. **n-s,** *Areg*$^{-/-}$ mice were treated as in **a-g. n,** Evan's blue/TTC-stained heart slices; scale bar, 1 mm. **o,** Percentage of AAR relative to LV size. **p,** Infarct size as percentage of AAR. n-p: n = 7 *Areg*$^{-/-}$ mice/Veh-treated/ZT8, n = 7 *Areg*$^{-/-}$ mice/Veh-treated/ZT20, n = 6 *Areg*$^{-/-}$ mice/vada-treated/ZT8, and n = 6 *Areg*$^{-/-}$ mice/vada-treated/ZT20. **q-s,** Cardiac function in *Areg*$^{-/-}$ mice on day 14 post-MI. **q,** EF, FS, and GLS. **r,** ESV. **s,** B-mode LV long-axis images with 2D longitudinal strain analysis. q-s: n = 7 mice/group/time point. All data are mean ± s.e.m. Statistical analysis was all performed using two-way ANOVA. ns: not significant.

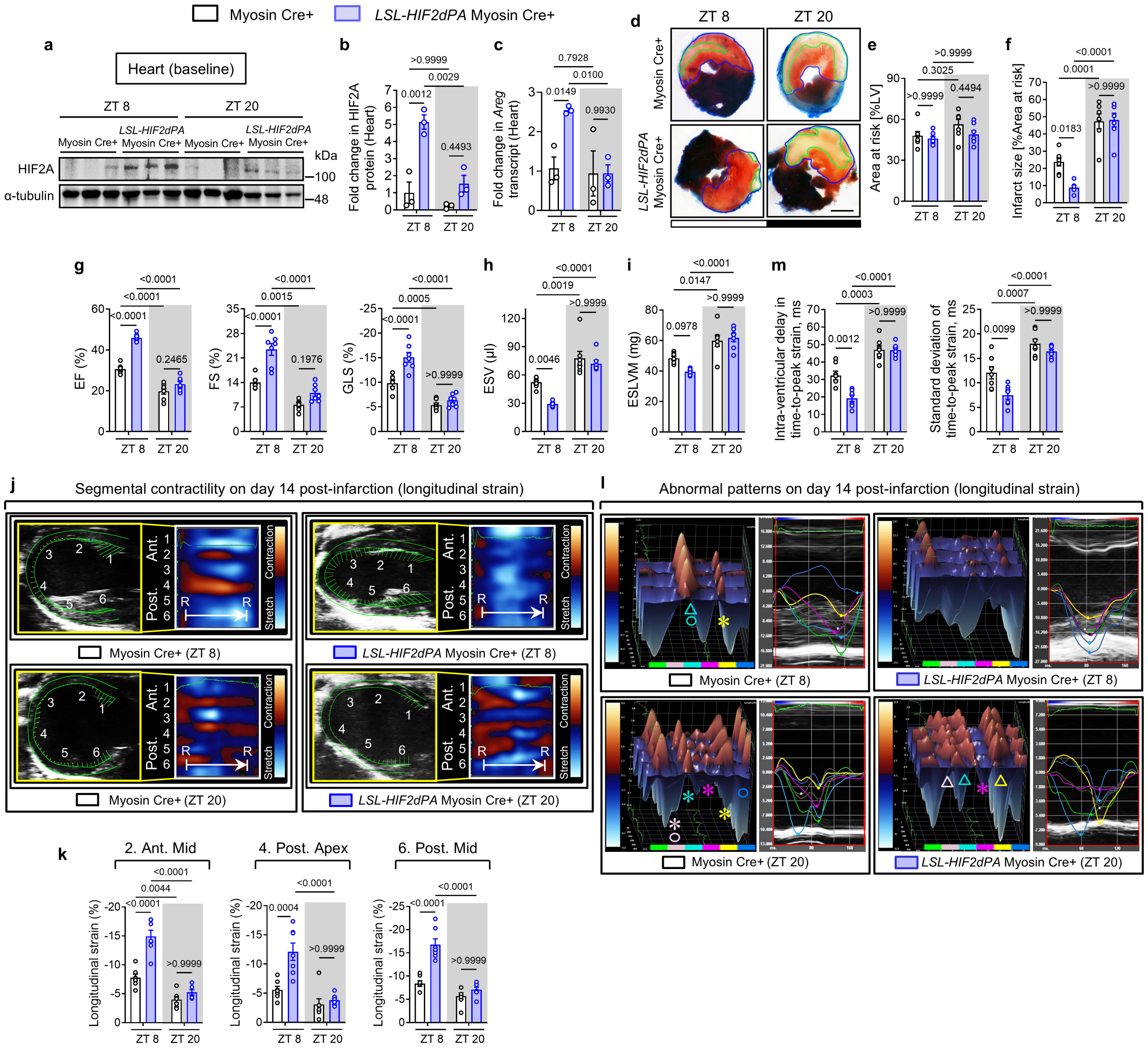

Fig. S9. Cardiomyocyte-specific *HIF2A* knock-in confers circadian-dependent cardioprotection in mice.

a and **b**, **a,** HIF2A protein levels in hearts from *LSL-HIF2dPA* (*HIF2A* knock-in) Myosin Cre+ mice and Myosin Cre+ mice were assessed by Western blotting (**a**), with quantification shown (**b**). n = 3 mice/group/time point. **c,** *Areg* transcript levels by real-time PCR. n = 3 mice/time point. **d-f:** These mice were subjected to myocardial IRI at ZT8 or ZT20. **d,** Evan's blue/TTC-stained heart slices following 2h of reperfusion: infarct area (green line) and AAR (blue line); scale bar, 1 mm. **e,** Percentage of AAR relative to LV. **f,** Infarct size as percentage of AAR. d-f: n = 7 mice/group/time point. **g-m,** Cardiac function evaluated by STE on day 14 post-MI. **g,** EF, FS, and GLS. **h,** ESV. **i,** ESLVM. **j,** B-mode LV long-axis images with 2D longitudinal strain analysis showing 6 segments (1. Anterior Base; 2. Anterior Middle; 3. Anterior Apex; 4. Posterior Apex; 5. Posterior Middle; 6. Posterior Base). **k,** LV segmental wall contractility. **l,** LV 3D (left) and six-segment longitudinal strain images (right) demonstrating wall motion abnormalities. Symbols indicate specific wall motion abnormalities: stars for reduced contractility, triangles for dyskinesis, and circles for dyssynchrony. Color-coded segments: Anterior Base (dark blue), Anterior Mid (yellow), Anterior Apex (magenta), Posterior Apex (cyan), Posterior Mid (light pink), and Posterior Base (green). **m,** LV mechanical dyssynchrony.

g-m: n = 7 mice/group/time point. All data are mean ± s.e.m. Statistical analysis was all performed using two-way ANOVA.

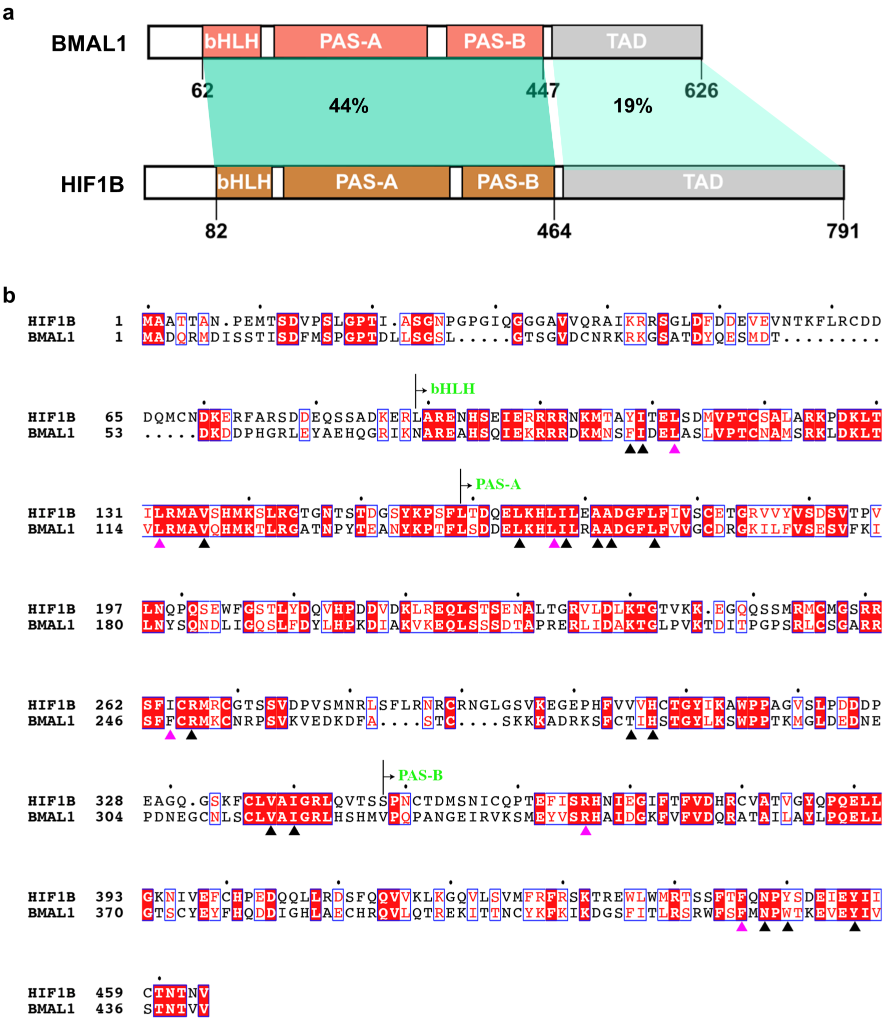

Fig. S10. Sequence alignment between BMAL1 and HIF1B.

a, Sequence identities of the bHLH-PAS and the C-terminal regions between BMAL1 and HIF1B. **b,** Sequence alignment of the N-terminal regions between BMAL1 and HIF1B. The conserved key residues of BMAL1 involved in HIF2A binding are indicated by triangles. Residues marked with magenta triangles indicate BMAL1 mutations that disrupt the interaction with HIF2A.

Supplementary Note 2: Supplementary Tables

Supplementary Table 1. Full gene list for mouse RNA-seq analysis.

Supplementary Table 2. GO enrichment analysis for mouse RNA-seq study.

Supplementary Table 3. Patient demographics, preoperative and intraoperative characteristics in human RNA-seq study.

	**Morning surgery (n = 56)**	**Afternoon surgery (n = 17)**	**p-value**
**Demographics**			
Sample collected time (median)	10:32 am	5:15 pm	
Age, years	70.57 (13.47)	72.23 (11.32)	0.85
Male sex	32 (57.14%)	13 (76.47%)	0.25
**Risk factors and comorbidities**			
Diabetes mellitus	20 (35.71%)	5 (29.41%)	0.77
Hypertension	44 (78.57%)	9 (52.94%)	0.06
Renal disease	3 (5.36%)	0 (0.00%)	0.99
Pulmonary disease	12 (22.64%)	3 (17.65%)	0.99
Smoking	31 (55.36%)	5 (29.41%)	0.10
**Cardiac status**			
New York Heart Association class	2.38 (0.63)	2.59 (0.51)	0.30
Left ventricular ejection fraction, %	60.59 (7.82)	58.69 (13.59)	0.78
Chronic atrial fibrillation	7 (12.50%)	3 (17.65%)	0.69
paroxysmal atrial fibrillation	3 (5.36%)	0 (0.00%)	0.99
Previous MI	4 (7.41%)	1 (5.88%)	0.99
**Preoperative medication**			
Aspirin	33 (64.71%)	11 (78.57%)	0.52
Beta-blockers	30 (58.82%)	7 (50.00%)	0.56
Angiotensin-converting enzyme inhibitors	23 (45.10%)	6 (42.86%)	0.99
Statins	40 (78.43%)	11 (78.57%)	0.99
Diuretics	22 (41.51%)	6 (42.86%)	0.99
**Surgery characteristics**			
Cardiopulmonary bypass duration, min	100.40 (35.33)	102.20 (42.33)	0.92
Aortic cross-clamp duration, min	78.36 (24.33)	82.12 (36.36)	0.90
Concomitant coronary artery bypass graft	23 (41.07%)	9 (52.94%)	0.42

Data are mean (SD) or n (%).

Supplementary Table 4. Full gene list in human RNA-seq study conducted on pre-clamping patient samples.

Supplementary Table 5. Covariates data in human RNA-seq study.

Supplementary Table 6. Full gene list in human RNA-seq study conducted on post-clamping patient samples.

Supplementary Table 7. GO enrichment analysis for human RNA-seq study conducted on post-clamping patient samples.

Supplementary Table 8. GO enrichment analysis for interactors with BMAL1.

Supplementary Table 9. Exclusively upregulated genes following myocardial IRI in Myosin Cre+ mice compared to the sham group.

Supplementary Table 10. PCR-based array analysis of the top 20 putative HIF2A target genes in mouse AAR following 2h of reperfusion.

	**Gene Symbol**	**Fold change (ZT8 vs.ZT20)**
1	*Areg*	5.336429281
2	*Camp*	4.776067317
4	*Ppp2r3a*	2.050049876
3	*Ereg*	1.385175458
5	*Fam84a*	1.089538281
6	*Ifit3*	1.008826154
7	*Olr1*	0.961149995
8	*Trim6*	0.906638861
9	*Cmpk2*	0.848709008
10	*Serpina3n*	0.796317083
11	*Ngp*	0.726639657
12	*4930402F11Rik*	0.699162661
13	*Gbp5*	0.680533104
14	*Otud1*	0.601659987
15	*Cd274*	0.541292184
16	*Ifit2*	0.516523501
17	*Csf3*	0.423441291
18	*Cxcl10*	0.423184196
19	*Rsad2*	0.397828297
20	*Cxcl11*	0.32245024

Supplementary Table 11. Cryo-EM data collection, processing, and refinement.

**Data collection**	
Microscope	Titan Krios
Voltage (KV)	300
Camera	GIF Quantum K2
Defocus range (μm)	1.0 to 2.5
Pixel size (Å)	0.85
Movies	18,039
Frames per movie	45
Exposure time per frame (ms)	200
Magnification	165,000x
Dose rate (e^-/pixel/sec)	6
Total dose per movie (e^-/Å^2)	75
**Data processing**	
Map	BMAL1/HIF2A/DNA
Particles	43,098
Symmetry imposed	C1
Map Resolution (Å)	3.6
Map sharpening B factor (Å^2)	-61
**Model building and refinement**	
Model	BMAL1/HIF2A/DNA
Initial model used (PDB)	4ZP4; 4F3L
Protein residues	512
Nucleotide	36
Metals	0
Other atoms	0
R.m.s deviations	
Bond angles (°)	0.727
Bond lengths (Å)	0.004
Ramachandran plot	
Favored (%)	91.29
Outlier (%)	0.00
Validation	
Clash score	9.29
MolProbity score	2.33

Supplementary Table 12. Key resources table

REAGENT or RESOURCE	SOURCE	IDENTIFIER
**Antibodies**		
Rabbit polyclonal anti-HIF1A	Novus Biologicals	Cat # NB100-479 RRID:AB_10000633
Mouse monoclonal anti-HIF1A	Novus Biologicals	Cat # NB100-105 RRID:AB_10001154
Rabbit monoclonal anti-HIF1A	Bethyl Laboratories	Cat # 700-001 RRID:AB_2631882
Rabbit polyclonal anti-HIF2A	Novus Biologicals	Cat # NB100-122 RRID:AB_10002593
Mouse monoclonal anti-HIF2A	Novus Biologicals	Cat # NB100-132 RRID:AB_10000898
Rabbit monoclonal anti-HIF2A	Bethyl Laboratories	Cat # 700-003 RRID:AB_2631884
Rabbit monoclonal anti-HIF1B	Cell Signaling Technology	Cat # 5537 RRID:AB_10694232
Rabbit monoclonal anti-BMAL1	Cell Signaling Technology	Cat # 14020 RRID:AB_2728705
Rabbit polyclonal anti-BMAL1	Abcam	Cat # ab3350 RRID:AB_303729
Rabbit monoclonal anti-CLOCK	Cell Signaling Technology	Cat # 5157 RRID:AB_10695411
Rabbit polyclonal anti-RORα	Abcam	Cat # ab60134 RRID:AB_945289
Mouse monoclonal anti-AREG	Santa Cruz Biotechnology	Cat # sc-74501 RRID:AB_1118939
Rabbit polyclonal anti-caspase-3	Cell Signaling Technology	Cat # 9662 RRID:AB_331439
Rabbit polyclonal anti-cleaved caspase-3	Cell Signaling Technology	Cat # 9661 RRID:AB_2341188
Rabbit polyclonal anti-Bax	Cell Signaling Technology	Cat # 2772 RRID:AB_10695870
Mouse monoclonal anti-FLAG	Sigma-Aldrich	Cat # F1804 RRID:AB_262044
Rabbit monoclonal anti-Lamin B1	Cell Signaling Technology	Cat # 12586 RRID:AB_2650517
Mouse monoclonal anti-Lamin A/C	Cell Signaling Technology	Cat # 4777 RRID:AB_10545756
Mouse monoclonal anti-Ubiquitin	Santa Cruz	Cat # 8017 RRID:AB_628423

Rabbit polyclonal anti-TBP	Cell Signaling Technology	Cat # 8515<br>RRID:AB_10949159
Rabbit polyclonal anti-α-tubulin	Cell Signaling Technology	Cat # 2144<br>RRID:AB_2210548
Mouse monoclonal anti-β-actin	Santa Cruz	Cat # 47778<br>RRID:AB_626632
Rabbit monoclonal IgG	Abcam	Cat # ab172730<br>RRID:AB_2687931
Mouse monoclonal IgG	Cell Signaling Technology	Cat # 5415<br>RRID:AB_10829607
Rabbit polyclonal anti-α-sarcomeric	Abcam	Cat # ab137346<br>RRID:AB_2909405
Rabbit monoclonal anti-vimentin	Cell Signaling Technology	Cat # 5741<br>RRID:AB_10695459
Rabbit monoclonal anti-α-smooth muscle actin	Cell Signaling Technology	Cat # 19245<br>RRID:AB_2734735
Alexa Fluor 488 conjugated WGA	ThermoFisher Scientific	Cat # W11261
Mouse monoclonal anti-His	ThermoFisher Scientific	Cat # MA1-21315
Mouse monoclonal anti-GST	Genscript	Cat # A00865<br>RRID:AB_914654
**Recombinant DNA**		
pcDNA3 *mHif2a*-P405A/P531A	Addgene	Plasmid # 18956
pcDNA3 *mHif2a*-P405A/P530V/N851A	Addgene	Plasmid # 44027
pcDNA3 *mHif2a*	Addgene	Plasmid # 18950
pLV6 *Bmal1*-luc	Addgene	Plasmid # 68833
pcDNA3 *mBmal1*	Addgene	Plasmid # 31367
pcDNA3 *mHif1a*-P402A/P577A/N813A	Addgene	Plasmid # 44028
pcDNA3 HA-Ubiquitin	Addgene	Plasmid # 18712
pGL4.22-PGK1-HRE dLUC	Addgene	Plasmid # 128095
**Chemicals**		
Polyethylene glycol (15)-hydroxy stearate	Millipore Sigma	Cat # 42966
Recombinant mouse AREG	R&D Systems	Cat # 989-AR
Dimethylallyl glycine	Cayman Chemical	Cat # 71210
2,3,5-Triphenyltetrazolium chloride	Sigma-Aldrich	Cat # T8877
Evans blue dye	Sigma-Aldrich	Cat # E2129
Paraformaldehyde	Millipore Sigma	Cat # P6148
Trypsin-EDTA	ThermoFisher Scientific	Cat #15400054
Heparin	StemCell Technologies	Cat # 07980
Anhydrous ethyl alcohol	Commercial Alcohols	Cat # P016EAAN
Hydrochloric acid (HCl)	Laboratoire Mat	Cat # CR-0166

Dimethyl sulfoxide	Millipore Sigma	Cat # D2650
Sodium Chloride (NaCl)	ThermoFisher Scientific	Cat # S271-3
Potassium chloride (KCl)	Millipore Sigma	Cat # P9541
Sodium bicarbonate ($NaHCO_3$)	Millipore Sigma	Cat # S5761
Sucrose	ThermoFisher Scientific	Cat # S5-3
Triton X-100	Amresco	Cat # M143
Protein A/G resin	ThermoFisher Scientific	Cat # 53133
anti-Flag M2 beads	Millipore Sigma	Cat # A2220
Glutathione magnetic agarose beads	GE Healthcare	Cat # 17-0756-01
Cycloheximide	Sigma-Aldrich	Cat # C4859
MG132	Sigma-Aldrich	Cat # M7449
Vadadustat	Cayman Chemical	Cat # 15295
**Critical commercial assays**		
NE-PER™ Nuclear and cytoplasmic extraction kit	ThermoFisher Scientific	Cat # 78835
SimpleChIP® enzymatic chromatin IP kit	Cell Signaling Technology	Cat # 9002
Mouse cardiac troponin-I SPARCL™ kit	Life Diagnostics	Cat # CTNI-SP-1
Click-iT™ Plus TUNEL assay kits	ThermoFisher Scientific	Cat # C10617
Duolink® In Situ PLA® Probe Anti-Rabbit PLUS	Millipore Sigma	Cat # DUO92002
Duolink® In Situ PLA® Probe Anti-Mouse MINUS	Millipore Sigma	Cat # DUO92004
Secrete-pair dual luminescence assay kit	GeneCopoeia	Cat # LF031
Dual-Luciferase® Reporter Assay System	Promega	Cat # E1690
**Experimental models: Cell lines**		
HEK-293	ATCC	Cat # CRL-1573
Human Cardiac Myocytes	ScienCell	Cat # 6200
**Experimental models: organisms/strains**		
Mouse: STOCK *A1cf*$^{Tg[Myh6–cre/Esr1*]1Jmk}$/J	Jackson	RRID:IMSR_JAX:005650
Mouse: B6.129-*Hif1a*^{tm3Rsjo}/J	Jackson	RRID:IMSR_JAX:007561
Mouse: STOCK *Epas1*^{tm1Mcs}/J	Jackson	RRID:IMSR_JAX:008407
Mouse: B6.129S4(Cg)-*Bmal1*^{tm1Weit}/J	Jackson	RRID:IMSR_JAX:007668
Mouse: B6;129-*Areg*^{tm1Dle}/Mmnc	MMRRC	MMRRC_011533-UNC

Mouse: STOCK *Gt(ROSA)26Sor*$^{tm4(HIF2A*)Kael}$/J	Jackson	RRID:IMSR_JAX:009674
**Oligonucleotides**		
HIF1A siRNA	ThermoFisher Scientific	Cat # 4390825 (s6539)
HIF2A siRNA	ThermoFisher Scientific	Cat # 4390825 (s4698)
BMAL1 siRNA	ThermoFisher Scientific	Cat # 4392420 (s1613)
HIF1B siRNA	ThermoFisher Scientific	Cat # 4392420 (s1616)
CLOCK siRNA	ThermoFisher Scientific	Cat # 4392420 (s18392)

Supplementary Note 3: Supplementary Video

Supplementary Video 1. Structural rearrangement of BMAL1 upon binding with various partners.

Supplementary Note 4: Supplementary Discussion

Acute injury and long-term outcomes of myocardial infarction exhibit a circadian pattern[1-6], yet the underlying mechanisms have remained elusive. The current studies newly identified a molecular mechanism governing this phenomenon: BMAL1 directly interacts with HIF2A to form a transcriptionally active heterodimer, which drives the circadian variation of myocardial injury in both the acute and long-term phases in mice post-MI. Our biophysical and biochemical studies further provide the structural basis for understanding how BMAL1 and HIF2A interact and recognize DNA. Intriguingly, we identify *Areg* as a rhythmic transcriptional target gene of the BMAL1/HIF2A heterodimer in determining the daytime variation of cardiac injury. Furthermore, BMAL1 plays a dual role by both enhancing HIF2A's transcriptional activity and stabilizing HIF2A under hypoxic conditions, thereby orchestrating the circadian modulation of hypoxic responses during myocardial injury. Leveraging this mechanistic insight, we demonstrate that pharmacological or genetic targeting of the BMAL1/HIF2A-AREG pathway significantly alleviates myocardial injury and improves cardiac function in a time-of-day-dependent manner.

In cardiomyocytes, the core circadian transcription factor BMAL1 is implicated in various aspects of cardiac health and diseases, including mitochondrial function[7-9], heart development[10], contractile function[7,8], cell death[11], and heart failure[2,7,12]. Despite this extensive involvement, a significant knowledge gap has persisted regarding its functional roles in the diurnal variation of myocardial injury post-MI. Our findings break new ground by demonstrating that cardiomyocyte-specific deletion of *Bmal1* eliminates inherent circadian variation in myocardial injury and cardiac function impairment during both the acute phase and extended

reperfusion periods. Our research primarily focuses on BMAL1 in cardiomyocytes, given its central role in cardiac dysfunction and post-MI heart failure. However, considering the prevalence of peripheral molecular clocks in numerous cell types within the heart[3], BMAL1's function in these cells may also contribute to the circadian modulation of myocardial injury. Studies have shown that endothelial BMAL1 governs diurnal variation in both thrombotic responses[13] and the activation of plasminogen activator inhibitor-1 (PAI-1)[14], a protein crucial for regulating the fibrinolytic system. Given the pivotal role of the dynamic balance between thrombus formation and fibrinolysis in increased MI risks and reduced efficacy of thrombolytic therapies during the morning hours, BMAL1's involvement in this context is of significant importance. Furthermore, BMAL1's role in circadian-dependent myocardial injury appears to be highly cell-specific. For instance, targeted deletion of *Bmal1* in smooth muscle cells disrupts myogenic reactivity, resulting in the suspension of myogenic tone at the circadian minimum[15]. This phenomenon leads to reduced total peripheral resistance and improved cardiac function post-MI[15]. Another study demonstrated that BMAL1 governs diurnal aging in neutrophils, and persistent aged neutrophils in the vasculature elevate thrombo-inflammation risks[16]. Thus, neutrophil-specific deletion of *Bmal1* results in defective aging, contributing to reduced infarction post-MI[16]. These findings, combined with our current research, underscore the diverse and highly cell-specific functions of BMAL1 in modulating circadian variations of myocardial injury.

While extensive research has focused on BMAL1’s interaction with its canonical partner, CLOCK, in the fine-tuning of clock-controlled genes, there remains a significant gap in our understanding of BMAL1's interactions with other partners in specific pathological contexts,

particularly during IRI. Although previous studies have demonstrated that BMAL1 can associate with both HIF1A and HIF2A to regulate gene expression[17-19], much of this research has been limited to cellular systems, leaving the functional significance of these interactions in vivo and under disease conditions not yet fully understood. For instance, BMAL1 has been shown to interact with HIF1A during hypoxia to regulate E-box-containing genes such as *Cry1*[19] and *Per2*[18,19]. Moreover, studies have indicated that BMAL1 can also form functional DNA-binding complexes with HIF2A[17,20,21]. Co-IP experiments have further indicated endogenous interactions between HIF2A and BMAL1[17,21], as well as between HIF1A and BMAL1[22] in cellular models. However, the direct and stable interaction between BMAL1 and HIF2A had not been conclusively demonstrated in a pathophysiological context until a recent study involving plateau pika[21], where a highland-adaptive mutation in HIF2A facilitated binding with BMAL1, forming a stable complex capable of recognizing E-box DNA[21]. Building on these findings, our work provides the first evidence of a circadian-dependent interaction between endogenous BMAL1 and HIF2A in human cardiomyocytes under hypoxic conditions, and in mouse AAR following myocardial IRI. This discovery offers new insights into the dynamic interplay between circadian and hypoxic regulation during IRI. To further gain a molecular-level understanding of heterodimerization of BMAL1 with HIF2A for DNA binding, we determined the cryo-EM structure of the BMAL1/HIF2A/DNA complex. This structure provides compelling structural evidence that supports the long-theorized formation of the BMAL1/HIF2A heterodimer and its intrinsic ability to bind to DNA. Future research can delve into potential therapeutic applications or interventions based on this structure, especially considering the significance of these molecules in circadian rhythms and the cellular hypoxia

response.

HIF2A plays a crucial role in the body's adaptive response to hypoxia[23-27]. Under normoxic conditions, HIF2A undergoes rapid degradation. In contrast, during hypoxic conditions, HIF2A is stabilized. Once stabilized, it dimerizes with HIF1B and initiates the transcription of target genes that confer protective effects during myocardial IRI[25,26,28]. Both genetic and pharmacological strategies aimed at inhibiting HIF degradation consistently demonstrated cardioprotection[26,29-34]. However, the precise stabilization pattern of HIF2A and its role in the diurnal variation of myocardial injury remains unclear. Our research reveals that hypoxic conditions and myocardial IRI induce circadian-dependent stabilization of HIF2A both in vitro and in vivo. Furthermore, we demonstrate that cardiomyocyte-specific deletion of *Hif2a*, similar to *Bmal1* ablation, abolishes the endogenous cardioprotection observed at ZT8 when HIF2A stabilization is significantly enhanced in control mice. Supporting this, our pharmacological studies show that vadadustat treatment selectively stabilizes HIF2A in mouse hearts at ZT8, resulting in circadian-dependent cardioprotection, accompanied by increased BMAL1 levels and a stronger BMAL1/HIF2A interaction at this time.

One of the key findings of our study is the mechanism driving the time-of-day-dependent stabilization of HIF2A. Specifically, BMAL1 stabilizes HIF2A by inhibiting its ubiquitin-mediated degradation, uncovering an additional layer of circadian regulation in hypoxic responses. BMAL1 thus plays a dual role by both enhancing HIF2A's transcriptional activity and stabilizing it post-translationally, thereby increasing the heart's resilience to ischemic stress in a time-dependent manner. Importantly, BMAL1's interaction with HIF2A under hypoxia does not disrupt the HIF2A/HIF1B interaction or its transcriptional activity. Instead, BMAL1

likely stabilizes HIF2A, creating an additional pool that interacts with BMAL1 at circadian peaks, such as ZT8, while maintaining the function of the HIF2A/HIF1B complex. These findings suggest that BMAL1 exerts its cardioprotective effects predominantly through the BMAL1/HIF2A complex, functioning independently of the traditional HIF2A/HIF1B axis. This highlights the therapeutic potential of targeting circadian biology in ischemic heart disease. By selectively activating HIF2A at specific times of the day, we may optimize its cardioprotective effects while minimizing side effects associated with prolonged use of HIF activators, such as polycythemia and increased blood viscosity, which potentially lead to a heightened risk of thromboembolism and MI[34,35], as well as other concerns like tumor growth and metabolic disturbances. However, further research is needed to translate these findings into viable clinical therapies.

Given the well-established circadian patterns of myocardial injury and the protective role of the BMAL1/HIF2A complex, it is crucial to consider how physiological factors, such as cortisol, might influence this rhythmic response. Studies have shown a strong correlation between the morning surge in circulating cortisol and the increased incidence and severity of MI[36,37]. Cortisol helps synchronize peripheral clocks in various tissues, including cardiomyocytes, by interacting with the glucocorticoid receptor (GR), which binds to glucocorticoid response elements (GREs) in the promoters of clock genes such as *PER1*[38,39] and *REV-ERBα*[40]. Although direct evidence of GR binding to *BMAL1* promoter is lacking, cortisol may influence BMAL1 indirectly through REV-ERBα, a known repressor of *BMAL1* transcription. Given the critical role of BMAL1/CLOCK in the regulation of circadian rhythms in cardiac function—including genes involved in metabolism, stress responses, and redox

homeostasis—it is plausible that cortisol, by synchronizing the cardiac clock, modulates the heart's response to the physiological stress during MI. Furthermore, considering BMAL1's essential role in HIF2A-mediated circadian-dependent cardioprotection, cortisol could also impact this pathway. However, the precise relationship between cortisol, BMAL1, and HIF2A during MI remains unclear. Further research is needed to elucidate how cortisol and circadian rhythms coordinate to regulate this axis and impact the heart's response to ischemic stress.

Ischemic tissue injury poses a significant challenge across various clinical scenarios. While patients undergoing cardiac surgery benefit from protective measures such as cardioplegia and hypothermia to reduce myocardial injury, those with acute MI experience severe tissue hypoxia. This hypoxia stabilizes HIF2A, which in turn elevates AREG levels in the hearts of ischemic heart disease patients[26]. Our previous studies confirm that AREG, a key target of HIF2A and a member of the epidermal growth factor family[41,42], significantly contributes to cardioprotection[26,43]. AREG is initially synthesized as a transmembrane precursor (pro-AREG), which is cleaved by ADAM proteases into soluble forms essential for autocrine and paracrine signaling[41]. Soluble AREG then interacts with epidermal growth factor receptor (EGFR, also referred to as ERBB1)[26,43], initiating a positive feedback loop ("autoinduction") that enhances *AREG* mRNA stability[44-46]. Hypoxia further activates ADAM proteases, promoting pro-AREG shedding and increasing soluble AREG production, which stabilizes *AREG* mRNA and elevates protein levels[47]. This mechanism likely contributes to the synchronous appearance of *AREG* mRNA and protein in hypoxic cardiomyocytes (Fig. 3g-i) and mouse hearts following IRI (Fig. 3b-d). Our findings show that exogenous recombinant AREG increases AREG protein levels in the AAR post-MI (Fig. 4k, l), suggesting that recombinant AREG may activate the same

feedback mechanism as endogenous AREG.

In addition to this autoinduction mechanism, several other factors appear to enhance *AREG* mRNA stability and facilitate rapid translation under hypoxia. For instance, RNA-binding proteins (RBPs), such as HuR, bind to AU-rich elements in the 3'UTR of *AREG* mRNA, protecting it from degradation[44], a process that is enhanced under hypoxia[48]. m^{6}A modification by methyltransferase-like 3 (METTL3)[49-51], along with the suppression of miR-33a-3p[52] Under hypoxic conditions, AREG mRNA stability is further increased. Additionally, hypoxia inhibits nonsense-mediated decay (NMD)[53], potentially extending the half-life of *AREG* mRNA[54] and allowing for prolonged translation. Mechanisms related to rapid translation initiation also contribute to the synchronized peaks of *AREG* mRNA and protein. AU-rich elements in the 3'UTR of *AREG* mRNA can expedite translation, particularly during stress responses like hypoxia[41,44]. Internal ribosome entry sites (IRES) may allow *AREG* mRNA to bypass the typical cap-dependent translation, enabling faster protein synthesis under hypoxic conditions when cap-dependent translation is typically downregulated[55,56]. Finally, HIF2A, beyond its role as a transcription factor, also participates in specialized translation initiation complexes, facilitating rapid AREG protein synthesis[57,58]. Together, these mRNA stabilization and rapid translation mechanisms likely explain the minimal time lag between *AREG* mRNA and protein expression at ZT8 in ischemic mouse hearts. This synchronized regulation ensures that AREG is rapidly available at critical times to support cardiomyocyte survival and adaptation during hypoxic stress, reinforcing its essential role in endogenous cardioprotection during IRI.

AREG exerts its cardioprotective effects primarily through its interaction with ERBB1,

which activates the phosphatidylinositol 3-kinase and Akt/Protein Kinase B (PI3K/Akt) signaling pathway, critical for cardiomyocyte survival and recovery post-injury[26]. Our study supports this, as we observed increased apoptotic cardiomyocytes in *Areg*-deficient mice and reduced apoptosis following recombinant AREG administration (Fig. 4 h, i, s, t). Furthermore, using *ErbB1* knockout mice, ERBB1 inhibitors such as AG1478, and the monoclonal antibody cetuximab (targeting ERBB1's extracellular domain), we consistently observed increased myocardial injury[26], mirroring the phenotype seen in *Areg*-deficient mice. Notably, AREG treatment proved ineffective in cardiomyocyte-specific *ErbB1* knockout mice, underscoring the necessity of ERBB1 for AREG's cardioprotective efficacy[43]. Intriguingly, we also found that HIF2A-dependent induction of ERBB1 protein in mouse hearts enhances cardioprotection by activating the PI3K/Akt signaling pathway[43], suggesting a dual role for HIF2A. HIF2A orchestrates AREG signaling through both transcriptional upregulation of *Areg* and enhancing ERBB1 protein levels post-transcriptionally. Collectively, our findings, together with previously published data[26,43], demonstrate that AREG's cardioprotective effects are critically dependent on its interaction with the ERBB1 receptor on cardiomyocytes.

While our study primarily focuses on AREG's role in cardiomyocytes, notably, AREG is also secreted by other cell types, including macrophages[59,60] and regulatory T cells (Tregs)[61], and participates in various biological processes. For instance, AREG promotes autophagy[62] and suppresses immune responses through Tregs[59,63], both of which are crucial for reducing inflammation and promoting tissue repair following myocardial injury. In addition, AREG's proangiogenic effects may aid in the restoration of blood flow post-injury[64]. These multifaceted functions highlight AREG's broader importance in cardiac biology, suggesting that targeting

AREG could provide novel therapeutic strategies by leveraging its effects across multiple cell types that contribute to cardiac protection and repair.

A novel aspect of our research is the revelation of a diurnal pattern in *AREG* induction, observed both in hypoxic cardiomyocytes and in ischemic mouse hearts. We further elucidate the core mechanism underlying this rhythmic induction: BMAL1 and HIF2A interact and bind directly to a highly conserved motif (CAGGTG) within the *AREG* promoter during ambient hypoxia. Moreover, this interaction is indispensable for the synergistic transactivation of *AREG*. Intriguingly, mice lacking *Areg* exhibit diminished daytime variations of myocardial injury post-MI, underscoring a circadian-dependent cardioprotective role of AREG. Thus, our research illuminates a previously unexplored, endogenous regulatory mechanism, wherein the BMAL1/HIF2A-AREG pathway dictates circadian variation in myocardial injury. We further demonstrate that timed AREG administration, particularly during its endogenous expression trough, presents an exciting avenue for enhancing cardioprotection. These findings highlight the importance of considering the timing of drug administration to optimize treatment strategies—especially when a strong correlation exists between the pharmacodynamic effect and the plasma or tissue levels of a drug targeting a circadian-regulated pathway[65]. This concept is highly relevant, considering that over half of the top-selling 100 drugs in the United States target genes with circadian rhythms and have half-lives of less than six hours[66]. For instance, nighttime administration of hypertensive medications and aspirin improves efficacy during the morning vulnerable period and reduces side effects[65,67,68]. Furthermore, in alignment with our findings that directly targeting circadian rhythm through pharmacological enhancement (NOB treatment) could reduce myocardial injury, other studies have shown that circadian rhythm

disruption, such as in shift work, exacerbates myocardial infarction reperfusion injury[69].

More importantly, our findings reveal that the cardioprotective effects of NOB and vadadustat are driven by the synergistic interaction between BMAL1 and HIF2A, rather than their independent actions. On top of these findings, our structural analysis of the BMAL1/HIF2A/DNA complex reveals the ability of BMAL1 to undergo significant conformational rearrangements when interacting with different partners in response to diverse regulatory conditions. This structural insight also lays the foundation for future targeted drug discovery aimed at enhancing the BMAL1/HIF2A interaction to potentially boost its cardioprotective effects. Taken together, our study unravels a novel mechanism by which the BMAL1/HIF2A-AREG axis modulates the circadian variation of myocardial injury. Targeting this pathway through chronotherapy—by optimizing the timing of drug administration or enhancing circadian rhythms—could significantly improve cardioprotection in MI treatment. Building on this, tailoring therapies to individual circadian profiles offers a promising advancement for precision medicine in cardiac care.

Supplementary References

1 Muller, J. E. *et al.* Circadian variation in the frequency of onset of acute myocardial infarction. *New England Journal of Medicine* **313**, 1315-1322 (1985).

2 El Jamal, N., Lordan, R., Teegarden, S. L., Grosser, T. & FitzGerald, G. The Circadian Biology of Heart Failure. *Circ Res* **132**, 223-237, doi:10.1161/CIRCRESAHA.122.321369 (2023).

3 Crnko, S., Du Pré, B. C., Sluijter, J. P. & Van Laake, L. W. Circadian rhythms and the molecular clock in cardiovascular biology and disease. *Nature Reviews Cardiology* **16**, 437-447 (2019).

4 Ruan, W., Yuan, X. & Eltzschig, H. K. Circadian rhythm as a therapeutic target. *Nat Rev Drug Discov* **20**, 287-307, doi:10.1038/s41573-020-00109-w (2021).

5 Reiter, R., Swingen, C., Moore, L., Henry, T. D. & Traverse, J. H. Circadian dependence of infarct size and left ventricular function after ST elevation myocardial infarction. *Circulation research* **110**, 105-110 (2012).

6 Thosar, S. S., Butler, M. P. & Shea, S. A. Role of the circadian system in cardiovascular disease. *J Clin*

Invest **128**, 2157-2167, doi:10.1172/JCI80590 (2018).
7 Young, M. E. *et al.* Cardiomyocyte-specific BMAL1 plays critical roles in metabolism, signaling, and maintenance of contractile function of the heart. *Journal of biological rhythms* **29**, 257-276 (2014).
8 Bray, M. S. *et al.* Disruption of the circadian clock within the cardiomyocyte influences myocardial contractile function, metabolism, and gene expression. *American Journal of Physiology-Heart and Circulatory Physiology* **294**, H1036-H1047 (2008).
9 Kohsaka, A. *et al.* The circadian clock maintains cardiac function by regulating mitochondrial metabolism in mice. *PLoS One* **9**, e112811 (2014).
10 Lefta, M., Campbell, K. S., Feng, H.-Z., Jin, J.-P. & Esser, K. A. Development of dilated cardiomyopathy in Bmal1-deficient mice. *American Journal of Physiology-Heart and Circulatory Physiology* **303**, H475-H485 (2012).
11 Rabinovich-Nikitin, I., Lieberman, B., Martino, T. A. & Kirshenbaum, L. A. Circadian-Regulated Cell Death in Cardiovascular Diseases. *Circulation* **139**, 965-980, doi:10.1161/CIRCULATIONAHA.118.036550 (2019).
12 Ingle, K. A. *et al.* Cardiomyocyte-specific Bmal1 deletion in mice triggers diastolic dysfunction, extracellular matrix response, and impaired resolution of inflammation. *American Journal of Physiology-Heart and Circulatory Physiology* **309**, H1827-H1836 (2015).
13 Westgate, E. J. *et al.* Genetic components of the circadian clock regulate thrombogenesis in vivo. *Circulation* **117**, 2087-2095 (2008).
14 Schoenhard, J. A. *et al.* Regulation of the PAI-1 promoter by circadian clock components: differential activation by BMAL1 and BMAL2. *Journal of molecular and cellular cardiology* **35**, 473-481, doi:10.1016/s0022-2828(03)00051-8 (2003).
15 Kroetsch, J. T. *et al.* Disrupting circadian control of peripheral myogenic reactivity mitigates cardiac injury following myocardial infarction. *Cardiovasc Res*, doi:10.1093/cvr/cvac174 (2022).
16 Adrover, J. M. *et al.* A Neutrophil Timer Coordinates Immune Defense and Vascular Protection. *Immunity* **50**, 390-402 e310, doi:10.1016/j.immuni.2019.01.002 (2019).
17 Hogenesch, J. B., Gu, Y.-Z., Jain, S. & Bradfield, C. A. The basic-helix–loop–helix-PAS orphan MOP3 forms transcriptionally active complexes with circadian and hypoxia factors. *Proceedings of the National Academy of Sciences* **95**, 5474-5479 (1998).
18 Wu, Y. *et al.* Reciprocal regulation between the circadian clock and hypoxia signaling at the genome level in mammals. *Cell metabolism* **25**, 73-85 (2017).
19 Peek, C. B. *et al.* Circadian Clock Interaction with HIF1alpha Mediates Oxygenic Metabolism and Anaerobic Glycolysis in Skeletal Muscle. *Cell Metab* **25**, 86-92, doi:10.1016/j.cmet.2016.09.010 (2017).
20 Saito, T. *et al.* Transcriptional regulation of endochondral ossification by HIF-2alpha during skeletal growth and osteoarthritis development. *Nat Med* **16**, 678-686, doi:10.1038/nm.2146 (2010).
21 Liu, N. *et al.* A highland-adaptation mutation of the Epas1 protein increases its stability and disrupts the circadian clock in the plateau pika. *Cell Rep* **39**, 110816, doi:10.1016/j.celrep.2022.110816 (2022).
22 Vaughan, M. E. *et al.* Cryptochromes Suppress HIF1alpha in Muscles. *iScience* **23**, 101338, doi:10.1016/j.isci.2020.101338 (2020).
23 Kaelin, W. G. & Ratcliffe, P. J. Oxygen sensing by metazoans: the central role of the HIF hydroxylase pathway. *Molecular cell* **30**, 393-402 (2008).
24 Pugh, C. W. & Ratcliffe, P. J. Regulation of angiogenesis by hypoxia: role of the HIF system. *Nature medicine* **9**, 677-684 (2003).

25 Ruan, W. *et al.* The Hypoxia-Adenosine Link during Myocardial Ischemia—Reperfusion Injury. *Biomedicines* **10**, 1939 (2022).
26 Koeppen, M. *et al.* Hypoxia-inducible factor 2-alpha-dependent induction of amphiregulin dampens myocardial ischemia-reperfusion injury. *Nature communications* **9**, 816 (2018).
27 Eltzschig, H. K. & Eckle, T. Ischemia and reperfusion—from mechanism to translation. *Nature medicine* **17**, 1391-1401 (2011).
28 Eltzschig, H. K., Bratton, D. L. & Colgan, S. P. Targeting hypoxia signalling for the treatment of ischaemic and inflammatory diseases. *Nat Rev Drug Discov* **13**, 852-869, doi:10.1038/nrd4422 (2014).
29 Du, M. *et al.* Renalase is a novel target gene of hypoxia-inducible factor-1 in protection against cardiac ischaemia–reperfusion injury. *Cardiovascular Research* **105**, 182-191 (2015).
30 Ong, S.-G. *et al.* HIF-1 reduces ischaemia–reperfusion injury in the heart by targeting the mitochondrial permeability transition pore. *Cardiovascular research* **104**, 24-36 (2014).
31 Adluri, R. S. *et al.* Disruption of hypoxia-inducible transcription factor-prolyl hydroxylase domain-1 (PHD-1−/−) attenuates ex vivo myocardial ischemia/reperfusion injury through hypoxia-inducible factor-1α transcription factor and its target genes in mice. *Antioxidants & redox signaling* **15**, 1789-1797 (2011).
32 Hölscher, M. *et al.* Cardiomyocyte-specific prolyl-4-hydroxylase domain 2 knock out protects from acute myocardial ischemic injury. *Journal of Biological Chemistry* **286**, 11185-11194 (2011).
33 Hyvärinen, J. *et al.* Hearts of hypoxia-inducible factor prolyl 4-hydroxylase-2 hypomorphic mice show protection against acute ischemia-reperfusion injury. *Journal of Biological Chemistry* **285**, 13646-13657 (2010).
34 Yuan, X., Ruan, W., Bobrow, B., Carmeliet, P. & Eltzschig, H. K. Targeting hypoxia-inducible factors: therapeutic opportunities and challenges. *Nat Rev Drug Discov* **23**, 175-200, doi:10.1038/s41573-023-00848-6 (2024).
35 Chertow, G. M. *et al.* Vadadustat in Patients with Anemia and Non-Dialysis-Dependent CKD. *N Engl J Med* **384**, 1589-1600, doi:10.1056/NEJMoa2035938 (2021).
36 Crawford, A. A. *et al.* Morning plasma cortisol as a cardiovascular risk factor: findings from prospective cohort and Mendelian randomization studies. *European journal of endocrinology* **181**, 429-438 (2019).
37 Mohd Azmi, N. A. S. *et al.* Cortisol on circadian rhythm and its effect on cardiovascular system. *International journal of environmental research and public health* **18**, 676 (2021).
38 Balsalobre, A. *et al.* Resetting of circadian time in peripheral tissues by glucocorticoid signaling. *Science* **289**, 2344-2347, doi:10.1126/science.289.5488.2344 (2000).
39 So, A. Y., Bernal, T. U., Pillsbury, M. L., Yamamoto, K. R. & Feldman, B. J. Glucocorticoid regulation of the circadian clock modulates glucose homeostasis. *Proc Natl Acad Sci U S A* **106**, 17582-17587, doi:10.1073/pnas.0909733106 (2009).
40 Torra, I. P. *et al.* Circadian and glucocorticoid regulation of Rev-erbalpha expression in liver. *Endocrinology* **141**, 3799-3806, doi:10.1210/endo.141.10.7708 (2000).
41 Berasain, C. & Avila, M. A. Amphiregulin. *Semin Cell Dev Biol* **28**, 31-41, doi:10.1016/j.semcdb.2014.01.005 (2014).
42 Shoyab, M., Plowman, G. D., McDonald, V. L., Bradley, J. G. & Todaro, G. J. Structure and function of human amphiregulin: a member of the epidermal growth factor family. *Science* **243**, 1074-1076 (1989).
43 Lee, J. W. *et al.* Transcription-independent induction of ERBB1 through hypoxia-inducible factor 2A provides cardioprotection during ischemia and reperfusion. *Anesthesiology* **132**, 763-780 (2020).

44 Nakayama, H. *et al.* Human antigen R-mediated mRNA stabilization is required for ultraviolet B-induced autoinduction of amphiregulin in keratinocytes. *Journal of Biological Chemistry* **288**, 10338-10348 (2013).

45 Huang, L. *et al.* Positive feedback of the amphiregulin-EGFR-ERK pathway mediates PM2.5 from wood smoke-induced MUC5AC expression in epithelial cells. *Sci Rep* **7**, 11084, doi:10.1038/s41598-017-11541-1 (2017).

46 Kefaloyianni, E. *et al.* Proximal Tubule-Derived Amphiregulin Amplifies and Integrates Profibrotic EGF Receptor Signals in Kidney Fibrosis. *J Am Soc Nephrol* **30**, 2370-2383, doi:10.1681/ASN.2019030321 (2019).

47 Uetani, T. *et al.* Insufficiency of pro-heparin-binding epidermal growth factor-like growth factor shedding enhances hypoxic cell death in H9c2 cardiomyoblasts via the activation of caspase-3 and c-Jun N-terminal kinase. *J Biol Chem* **284**, 12399-12409, doi:10.1074/jbc.M900463200 (2009).

48 Masuda, K., Abdelmohsen, K. & Gorospe, M. RNA-binding proteins implicated in the hypoxic response. *J Cell Mol Med* **13**, 2759-2769, doi:10.1111/j.1582-4934.2009.00842.x (2009).

49 Chen, Y. *et al.* METTL3 facilitates renal cell carcinoma progression by PLOD2 m(6)A-methylation under prolonged hypoxia. *Cell Death Dis* **15**, 62, doi:10.1038/s41419-023-06411-w (2024).

50 Su, X. *et al.* miR-33a-3p regulates METTL3-mediated AREG stability and alters EMT to inhibit pancreatic cancer invasion and metastasis. *Sci Rep* **13**, 13587, doi:10.1038/s41598-023-39506-7 (2023).

51 Sun, P. *et al.* Extracellular vesicle-packaged mitochondrial disturbing miRNA exacerbates cardiac injury during acute myocardial infarction. *Clin Transl Med* **12**, e779, doi:10.1002/ctm2.779 (2022).

52 Guo, X. F., Wang, A. Y. & Liu, J. HIFs-MiR-33a-Twsit1 axis can regulate invasiveness of hepatocellular cancer cells. *Eur Rev Med Pharmacol Sci* **20**, 3011-3016 (2016).

53 Gardner, L. B. Hypoxic inhibition of nonsense-mediated RNA decay regulates gene expression and the integrated stress response. *Mol Cell Biol* **28**, 3729-3741, doi:10.1128/MCB.02284-07 (2008).

54 Yaojia Cheng, Y. X. *et al.* Aberrant expression of the UPF1 RNA surveillance gene disturbs keratinocyte homeostasis by stabilizing AREG. *Int J Mol Med* **45**, 1163-1175, doi:10.3892/ijmm.2020.4487 (2020).

55 Stein, I. *et al.* Translation of vascular endothelial growth factor mRNA by internal ribosome entry: implications for translation under hypoxia. *Mol Cell Biol* **18**, 3112-3119, doi:10.1128/MCB.18.6.3112 (1998).

56 King, H. A., Cobbold, L. C. & Willis, A. E. The role of IRES trans-acting factors in regulating translation initiation. *Biochem Soc Trans* **38**, 1581-1586, doi:10.1042/BST0381581 (2010).

57 Uniacke, J. *et al.* An oxygen-regulated switch in the protein synthesis machinery. *Nature* **486**, 126-129, doi:10.1038/nature11055 (2012).

58 Uniacke, J., Perera, J. K., Lachance, G., Francisco, C. B. & Lee, S. Cancer cells exploit eIF4E2-directed synthesis of hypoxia response proteins to drive tumor progression. *Cancer Res* **74**, 1379-1389, doi:10.1158/0008-5472.CAN-13-2278 (2014).

59 Ko, J. H., Kim, H. J., Jeong, H. J., Lee, H. J. & Oh, J. Y. Mesenchymal Stem and Stromal Cells Harness Macrophage-Derived Amphiregulin to Maintain Tissue Homeostasis. *Cell Rep* **30**, 3806-3820 e3806, doi:10.1016/j.celrep.2020.02.062 (2020).

60 Minutti, C. M. *et al.* A Macrophage-Pericyte Axis Directs Tissue Restoration via Amphiregulin-Induced Transforming Growth Factor Beta Activation. *Immunity* **50**, 645-654 e646, doi:10.1016/j.immuni.2019.01.008 (2019).

61 Burzyn, D. *et al.* A special population of regulatory T cells potentiates muscle repair. *Cell* **155**, 1282-1295, doi:10.1016/j.cell.2013.10.054 (2013).

62 Li, N. *et al.* Amphiregulin improves ventricular remodeling after myocardial infarction by modulating autophagy and apoptosis. *FASEB J* **38**, e23488, doi:10.1096/fj.202302385R (2024).

63 Zaiss, D. M. *et al.* Amphiregulin enhances regulatory T cell-suppressive function via the epidermal growth factor receptor. *Immunity* **38**, 275-284 (2013).

64 Thapa, S. *et al.* Amphiregulin Exerts Proangiogenic Effects in Developing Murine Lungs. *Antioxidants (Basel)* **13**, doi:10.3390/antiox13010078 (2024).

65 Cederroth, C. R. *et al.* Medicine in the fourth dimension. *Cell metabolism* **30**, 238-250 (2019).

66 Zhang, R., Lahens, N. F., Ballance, H. I., Hughes, M. E. & Hogenesch, J. B. A circadian gene expression atlas in mammals: implications for biology and medicine. *Proceedings of the National Academy of Sciences* **111**, 16219-16224 (2014).

67 Tofler, G. H. *et al.* Concurrent morning increase in platelet aggregability and the risk of myocardial infarction and sudden cardiac death. *New England Journal of Medicine* **316**, 1514-1518 (1987).

68 Hermida, R. C., Ayala, D. E., Mojón, A. & Fernández, J. R. Influence of circadian time of hypertension treatment on cardiovascular risk: results of the MAPEC study. *Chronobiology international* **27**, 1629-1651 (2010).

69 Zhao, Y. *et al.* Disruption of circadian rhythms by shift work exacerbates reperfusion injury in myocardial infarction. *Journal of the American College of Cardiology* **79**, 2097-2115 (2022).

Supplementary Note 5: Uncropped gel blot images for main Figures

Fig. 2b.

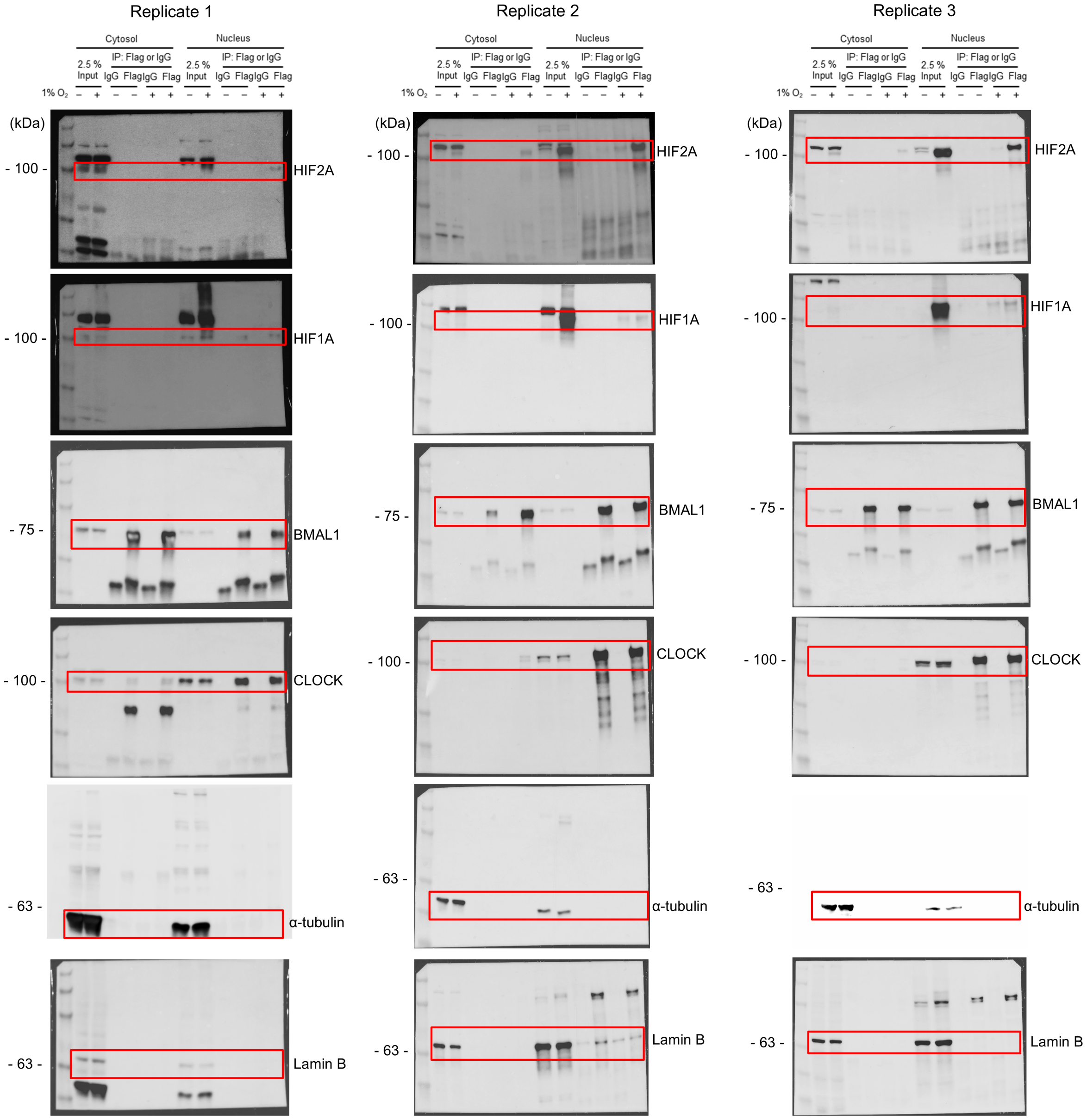

Uncropped western blot (WB) images for Fig. 2b. Co-IP analysis of BMAL1-Flag interaction with HIF2A, HIF1A, and CLOCK in nuclear and cytosolic fractions of HEK293 cells transfected with BMAL1-Flag, under normoxic or hypoxic conditions (1% O_2, 4 h) (n = 3 biological replicates). Samples were run on the same gels, with α-tubulin used as a loading control for cytosolic extracts and Lamin B used as a loading control for nuclear extracts.

Fig. 2d.

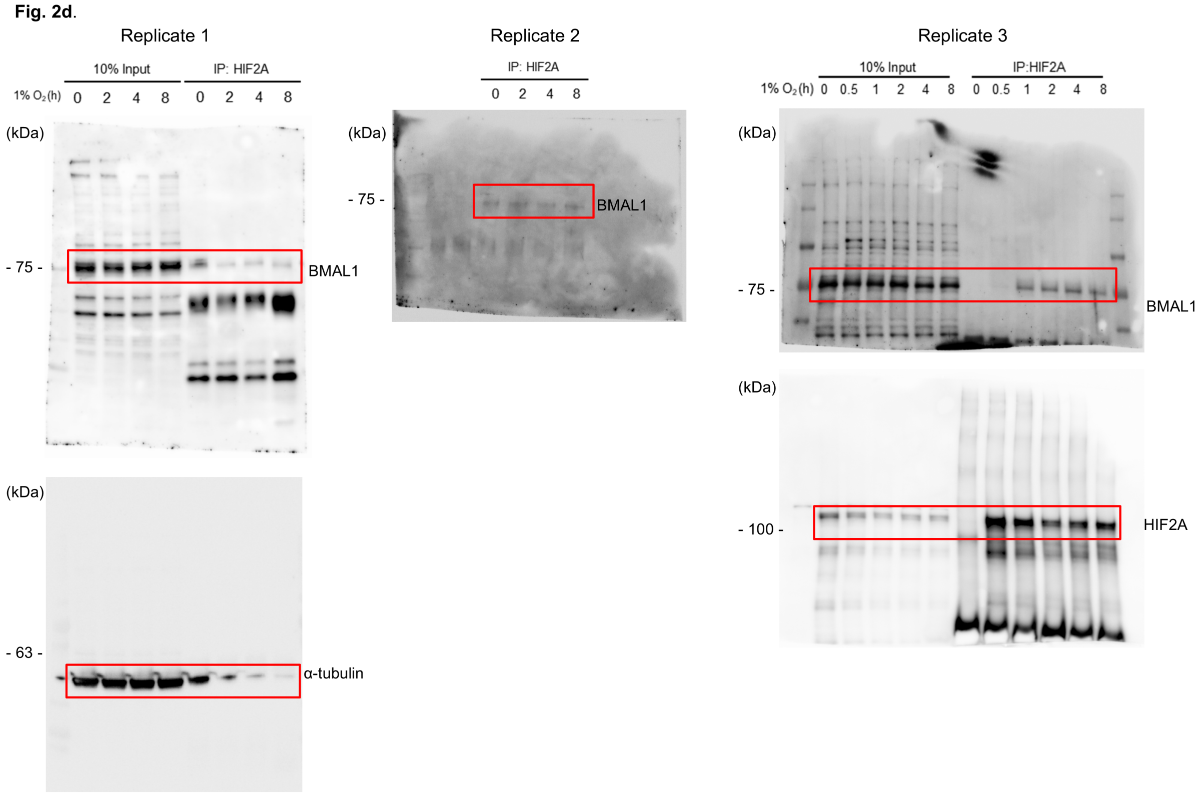

Uncropped WB images for Fig. 2d. Co-IP of HIF2A and BMAL1 in hypoxia-treated HCMs (n = 3 biological replicates). Samples were run on same gels, with α-tubulin as loading controls.

Fig. 2h

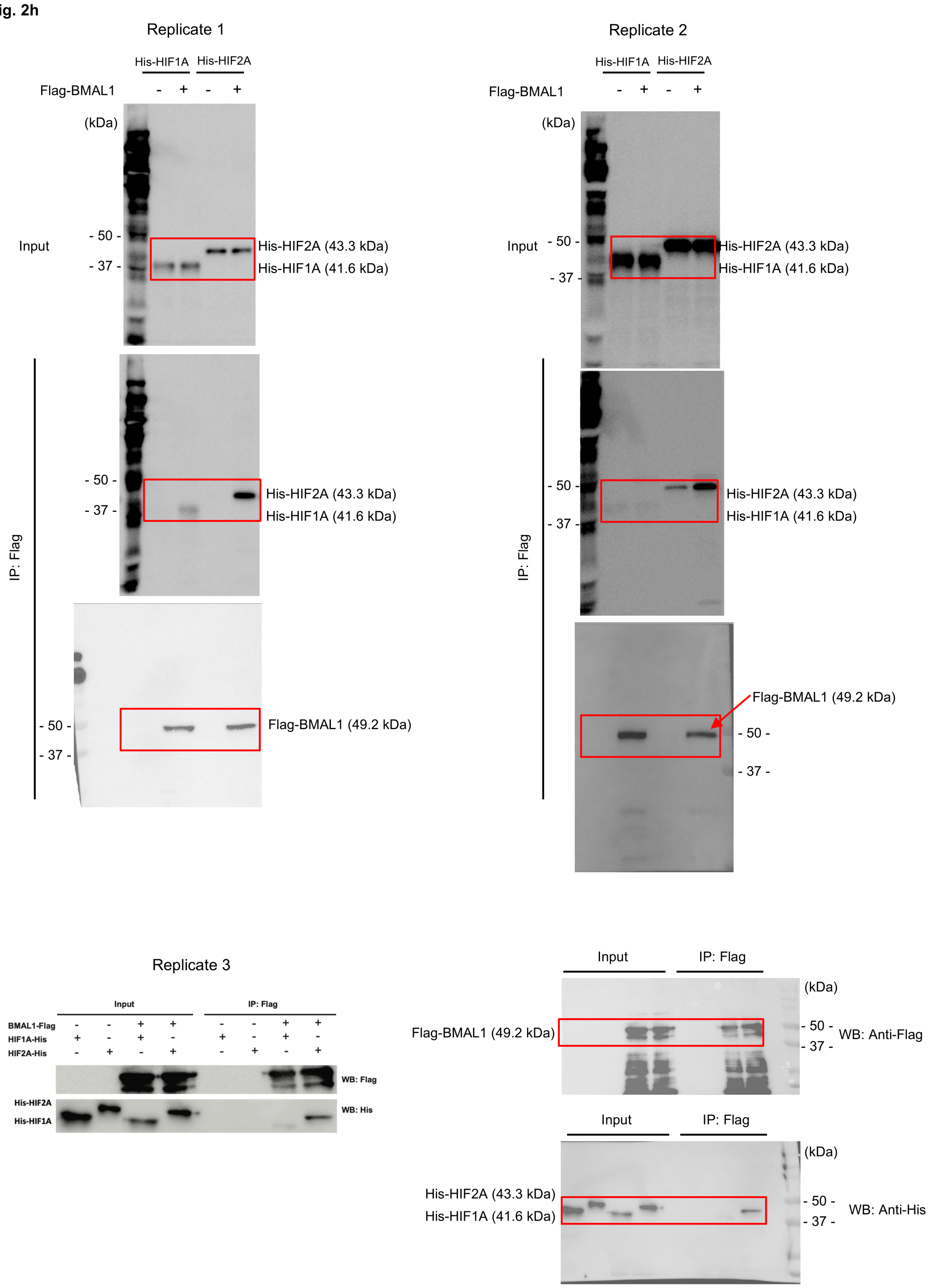

Uncropped WB images for Fig. 2h. An interaction analysis of BMAL1 with HIF2A and HIF1A. His-tagged HIF2A and HIF1A, along with Flag-tagged BMAL1 (bait), were detected by western blotting using anti-His and anti-flag antibodies, respectively (n = 3 biological replicates). Due to the similar molecular weights of these proteins, the samples were run and detected on separate gels with equal loading volumes. The input levels of HIF2A and HIF1A were normalized.

Fig. 2i

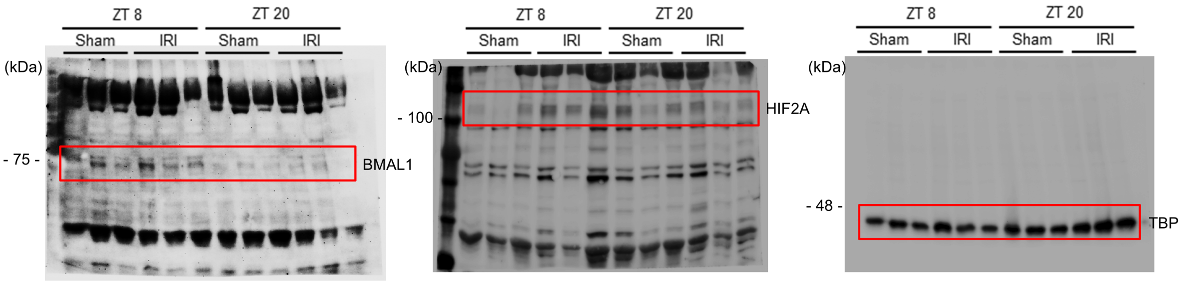

Uncropped WB images for Fig. 2i. BMAL1 and HIF2A nuclear protein levels in the AAR of C57BL/6J mice subjected to IRI at ZT8 or ZT20 assessed by Western blot after 2h of reperfusion (n = 3 biological replicates). Due to the presence of non-specific bands, HIF2A was run and detected on a separate gel with equal loading volumes, using TBP as the sample processing control.

Fig. 3c

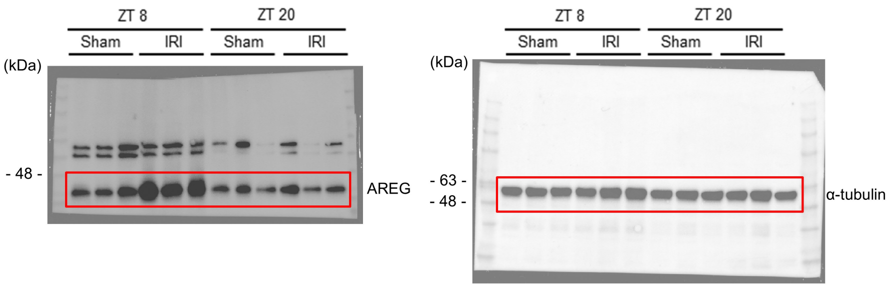

Uncropped WB images for Fig. 3c. AREG protein levels in cytosolic extracts were assessed by Western blot in the AAR of C57BL/6 mice subjected to IRI at ZT8 and ZT20 (n = 3 biological replicates). Due to the similar molecular weights of the target proteins, samples were run on separate gels with equal loading volumes, using α-tubulin as the sample processing control.

Fig. 3h

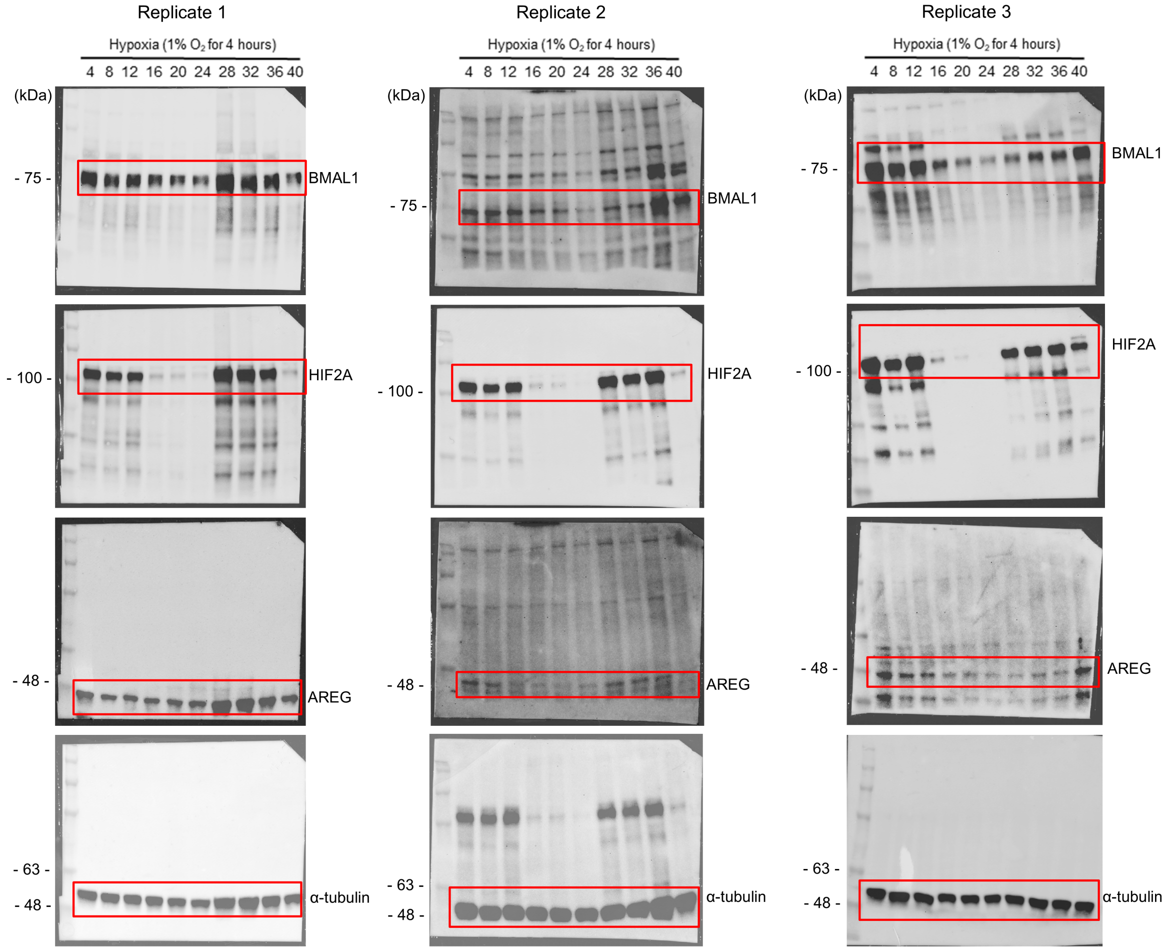

Uncropped WB images for Fig. 3h. Synchronized HCMs were exposed to hypoxia (1% O_2, 4 h) at various circadian times (CT0–CT40) as indicated, and protein levels of BMAL1, HIF2A, and AREG were assessed by Western blot (n = 3 biological replicates). Samples from replicates 1 and 2 were run on the same gels with α-tubulin serving as the loading control. Samples from replicate 3 were run on separate gels with equal loading volumes, using α-tubulin as the sample processing control.

Fig. 3I

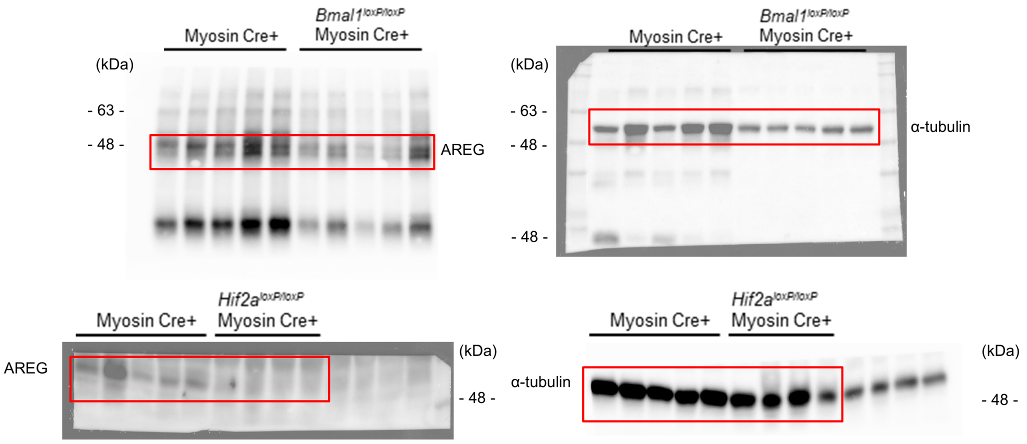

Uncropped WB images for Fig. 3l. AREG protein levels in Myosin Cre+ mice, *Bmal1*$^{loxP/loxP}$ Myosin Cre+ mice, and *Hif2a*$^{loxP/loxP}$ Myosin Cre+ mice after 2h of reperfusion (n = 5/Myosin Cre+ mice, n = 5/*Bmal1*$^{loxP/loxP}$ Myosin Cre+ mice, and n = 4 *Hif2a*$^{loxP/loxP}$ Myosin Cre+ mice, biological replicates). Samples were run on the same gels, with α-tubulin used as a loading control.

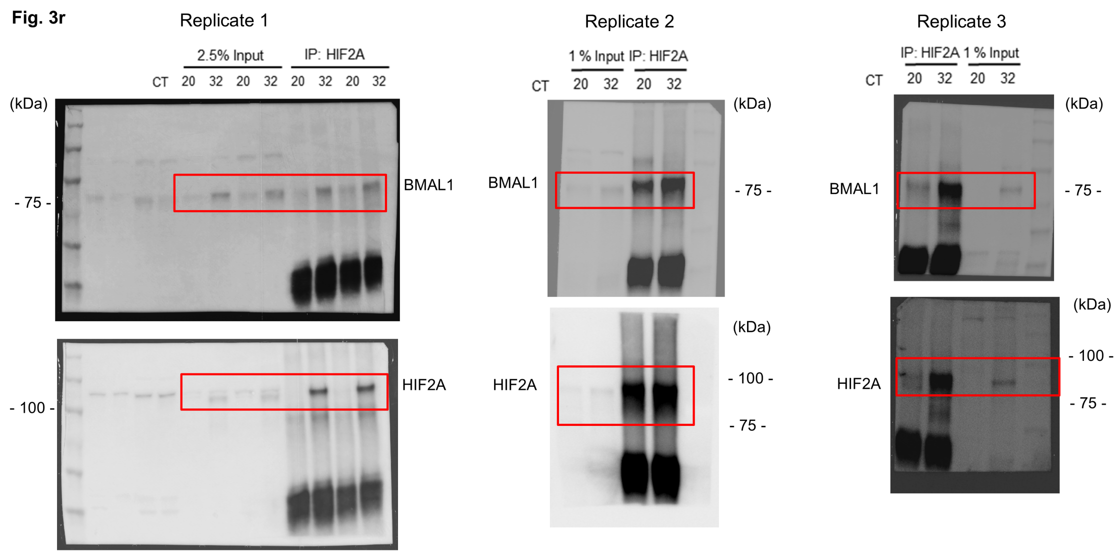

Uncropped WB images for Fig. 3r. Immunoprecipitation of nuclear protein extracts at CT20 or CT32 showing HIF2A-BMAL1 interaction (n = 3 biological replicates). Samples were run on the same gels with equal loading volumes.

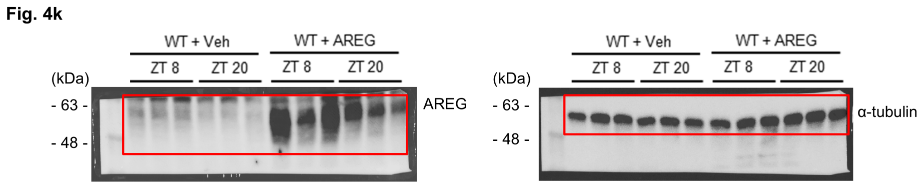

Uncropped WB images for Fig. 4k. AREG-treated or vehicle-treated mice were subjected to IRI at ZT8 or ZT20, and AREG protein levels in the AAR were assessed by Western blot (n = 3 biological replicates). Samples were run on the same gels, with α-tubulin used as a loading control.

Fig. 5e

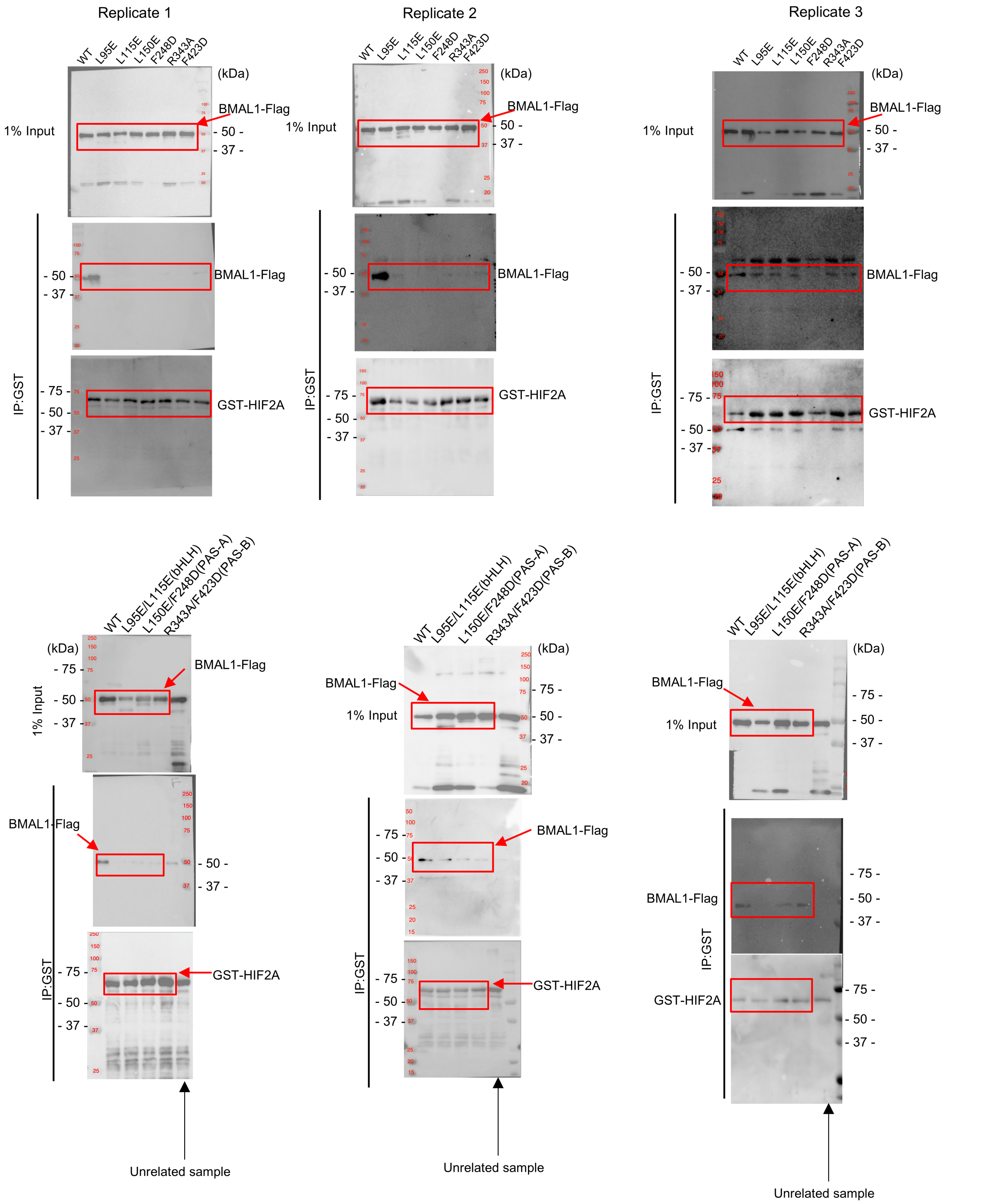

Uncropped WB images for Fig. 5e. Comparison of HIF2A interactions with wildtype BMAL1 and its mutants in vitro. GST-tagged HIF2A (bait) and Flag-tagged BMAL1 were detected by western blotting using anti-GST and anti-Flag antibodies, respectively (n = 3 biological replicates). Due to the difficulty of stripping antibodies from membranes, samples were run on separate gels with equal loading volumes. The input levels of wildtype BMAL1 and its mutants were normalized. Unrelated samples were indicated.

Fig. 5g

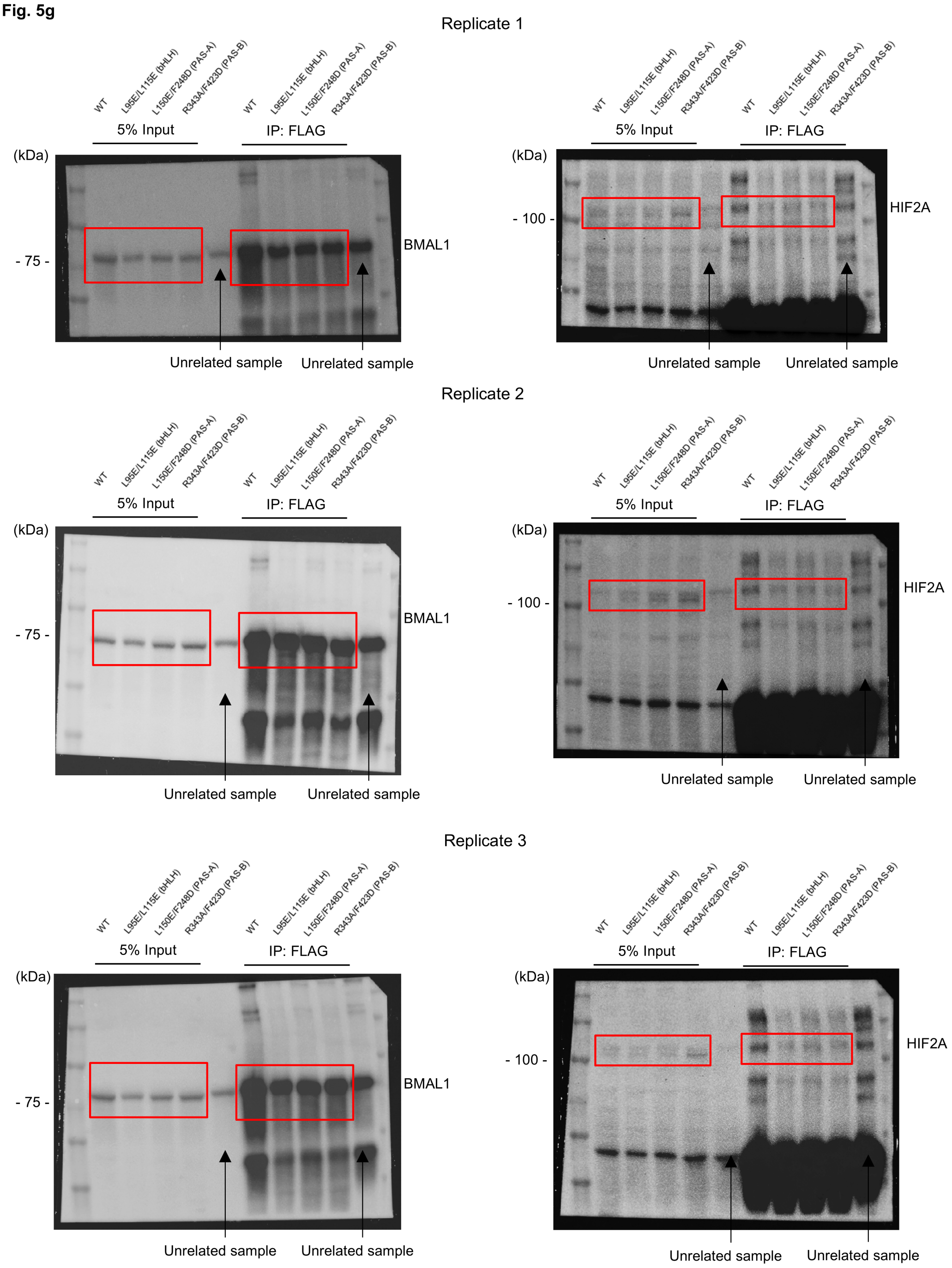

Uncropped WB images for Fig. 5g. HEK293 cells overexpressing WT or mutated Flag-tagged BMAL1 were exposed to ambient hypoxia (1% O_2) for 4h, then followed by immunoprecipitation with Flag antibody and blotted with BMAL1 and HIF2A antibodies (n = 3 biological replicates). Samples were run on the same gels with equal loading volumes. The input levels of wildtype BMAL1 and its mutants were normalized. Unrelated samples were indicated.

Supplementary Note 6: Uncropped gel blot images for Extended Data Figures.

Extended Data Fig. 2b

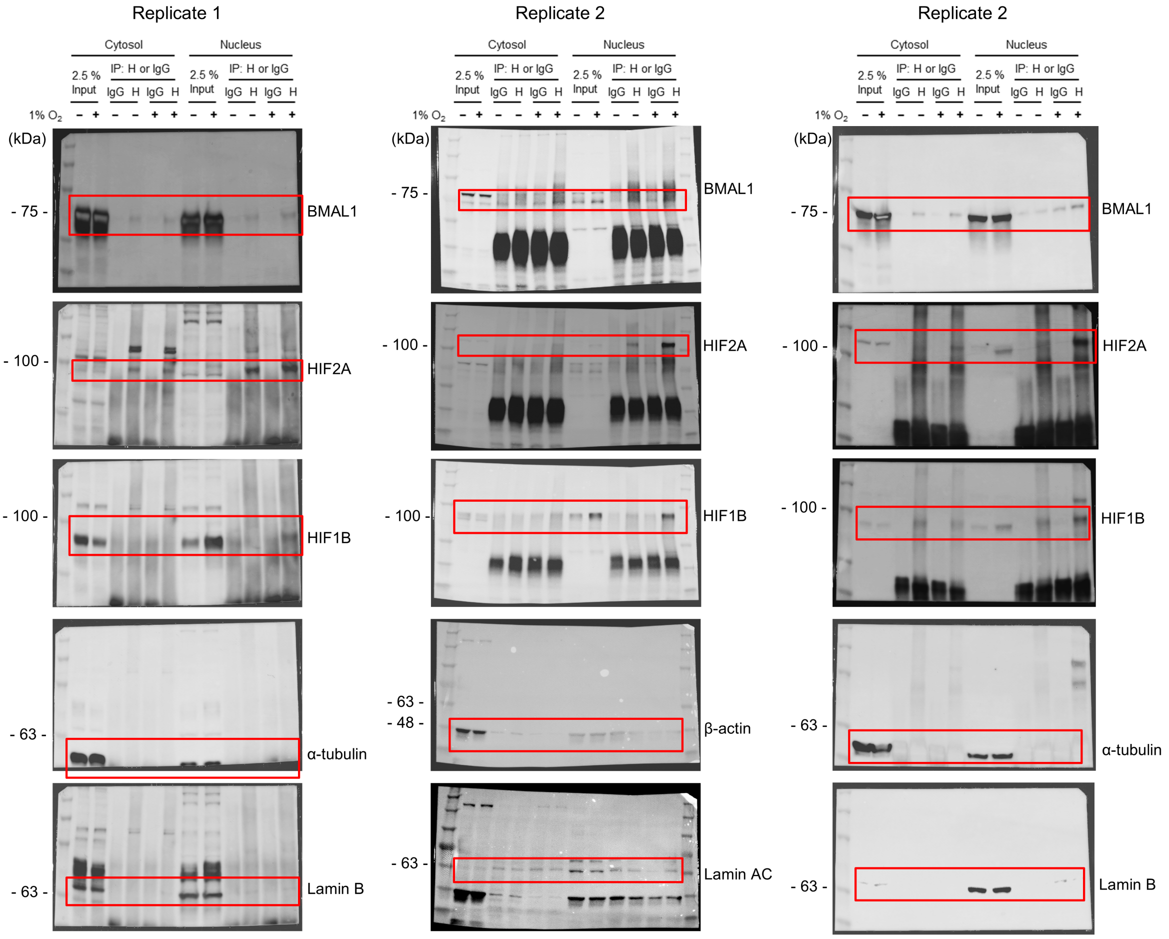

Uncropped WB images for Extended Data Fig. 2b. Western Blot analysis of reciprocal co-IP with HIF2A in hypoxia-treated (1% O_2, 4h) or normoxia-treated HEK293 cells. Cytosolic and nuclear protein extracts were immunoprecipitated with HIF2A and blotted with anti-BMAL1, anti-HIF2A, anti-HIF1B, anti-β-actin, anti-Lamin B, and anti-Lamin A/C antibodies (n = 3 biological replicates). Samples were run on the same gels, with α-tubulin or β-actin used as loading controls for cytosolic extracts, and Lamin B or Lamin A/C used as loading controls for nuclear extracts. An IgG control affirmed procedure specificity. H indicates HIF2A.

Extended Data Fig. 5c

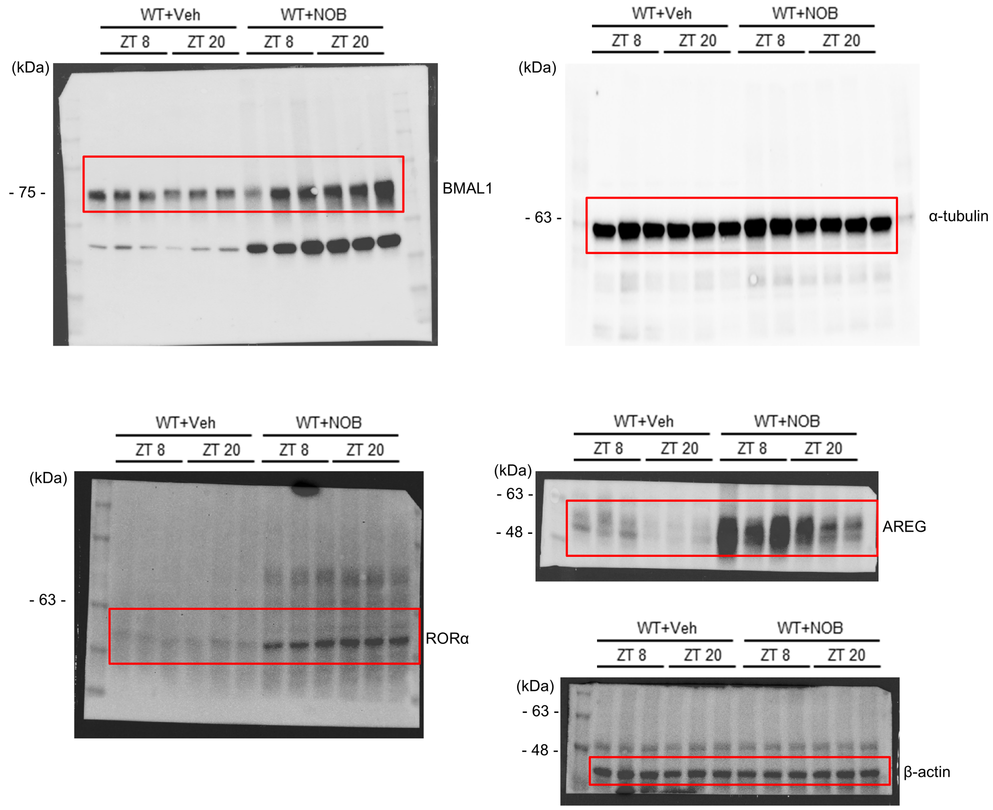

Uncropped WB images for Extended Data Fig. 5c. NOB-treated (200 mg/kg, i.p., every other day) or vehicle-treated C57BL/6J mice were subjected to myocardial IRI at ZT8 or ZT20. BMAL1, RORα, and AREG protein levels in the AAR, after 2 hours of reperfusion, were assessed by Western blot (n = 3 biological replicates). Due to similar molecular weight of proteins, some samples were run on separate gels with equal loading volumes, using α-tubulin or β-actin as sample processing controls.

Extended Data Fig. 6a

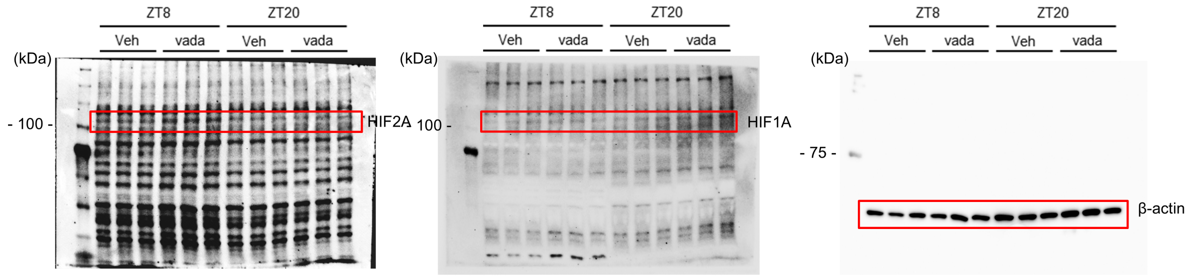

Uncropped WB images for Extended Data Fig. 6a. C57BL/6 mice were treated with vadadustat (Vada, 50 mg/kg, i.p., daily) or vehicle for three days at ZT8 or ZT20. HIF2A and HIF1A protein levels were assessed by Western blot in mouse hearts (n = 3 biological replicates). Samples were run on the same gels, with β-actin used as loading controls.

Extended Data Fig. 6o

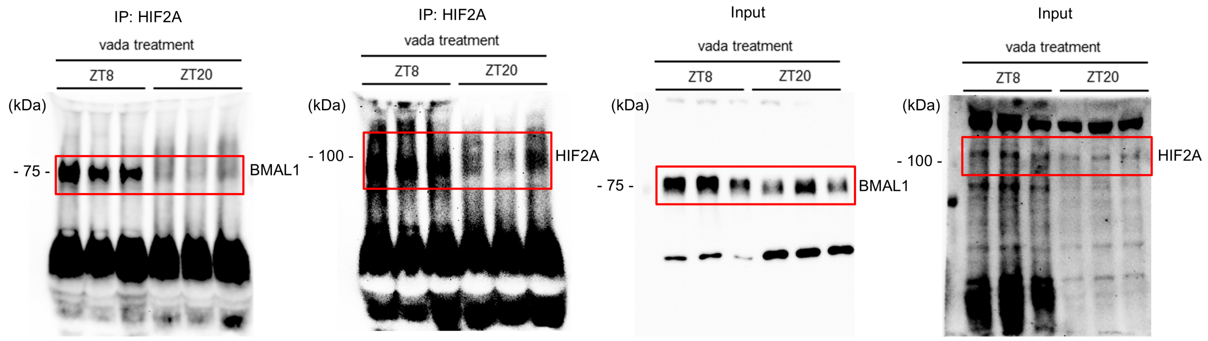

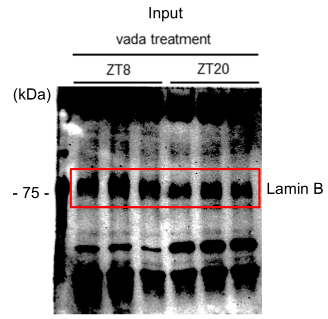

Extended Data Fig. 7e

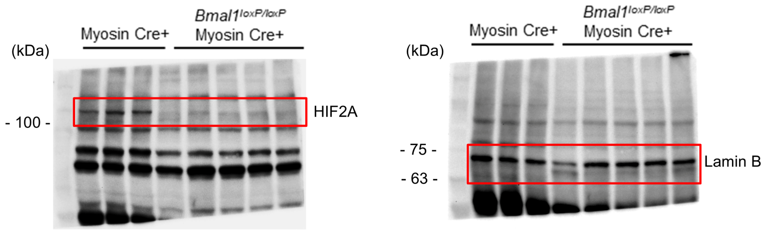

Uncropped WB images for Extended Data Fig. 6o. Mice treated with vadadustat for three days at ZT8 or ZT20, followed by IRI. Nuclear fractions from the AAR 3h post-reperfusion were immunoprecipitated with HIF2A and blotted for HIF2A, BMAL1, and Lamin B (n = 3 biological replicates). Samples were run on the same gels, with Lamin B used as loading controls.

Uncropped WB images for Extended Data Fig. 7e. HIF2A protein levels in the nuclear fractions of the AAR were assessed by Western blot after 2 hours of reperfusion in *Bmal1*$^{loxP/loxP}$ Myosin Cre+ mice and Myosin Cre+ mice (n = 3/Myosin Cre+ and n = 5/*Bmal1*$^{loxP/loxP}$ Myosin Cre+, biological replicates). Samples were run on the same gels, with Lamin B used as loading controls.

Extended Data Fig. 7g

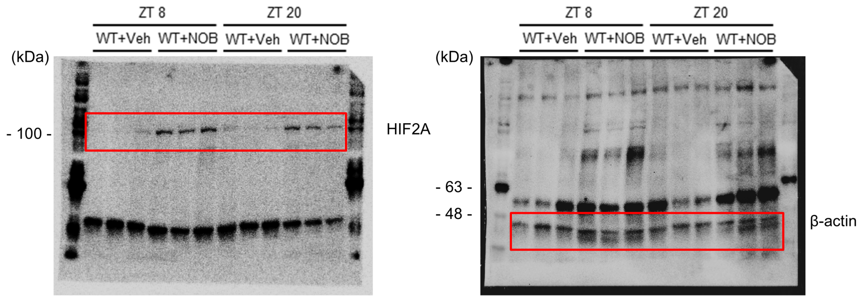

Uncropped WB images for Extended Data Fig. 7g. C57BL/6J mice treated with NOB (200 mg/kg, i.p.) or vehicle control, and HIF2A protein levels were assessed by Western blot at ZT8 or ZT20 (n = 3 biological replicates). Samples were run on the same gels, with β-actin used as loading controls.

Extended Data Fig. 7m (The β-actin blot is shared with **Extended Data Fig. 8j**)

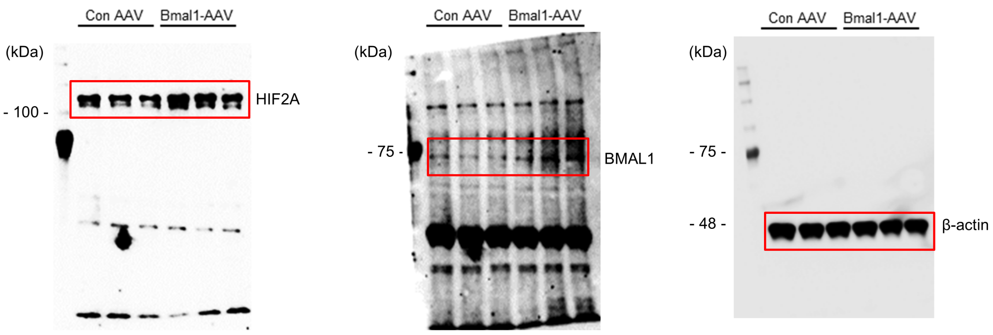

Uncropped WB images for Extended Data Fig. 7m. HCMs transduced with Bmal1-AAV or con-AAV were treated with 1% O_2 for 4 hours, and BMAL1 and HIF2A protein levels were assessed by Western blot (n = 3 biological replicates). Samples were run on the same gels, with β-actin used as loading controls. The samples used in **(m)** were also used in **Extended Data Fig. 8j**, sharing the β-actin blot.

Extended Data Fig. 7p (The β-actin blot is shared with **Extended Data Fig. 8m**)

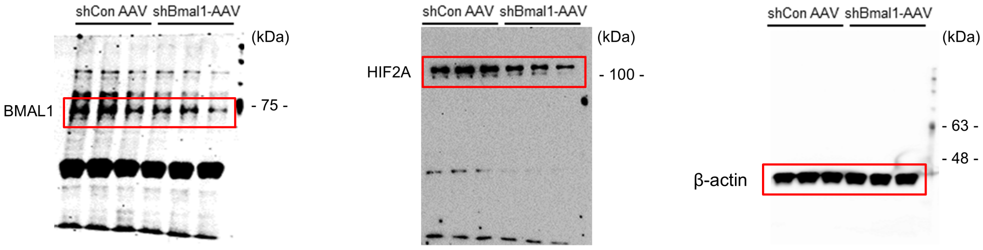

Uncropped WB images for Extended Data Fig. 7p. HCMs transduced with shBmal1-AAV or shCon AAV were treated with 1% O_2 for 4 hours, and BMAL1 and HIF2A protein levels were assessed by Western blot (n = 3 biological replicates). Samples were run on the same gels, with β-actin used as loading controls. The samples used in **(p)** were also used in **Extended Data Fig. 8m**, sharing the β-actin blot.

Extended Data Fig. 7s

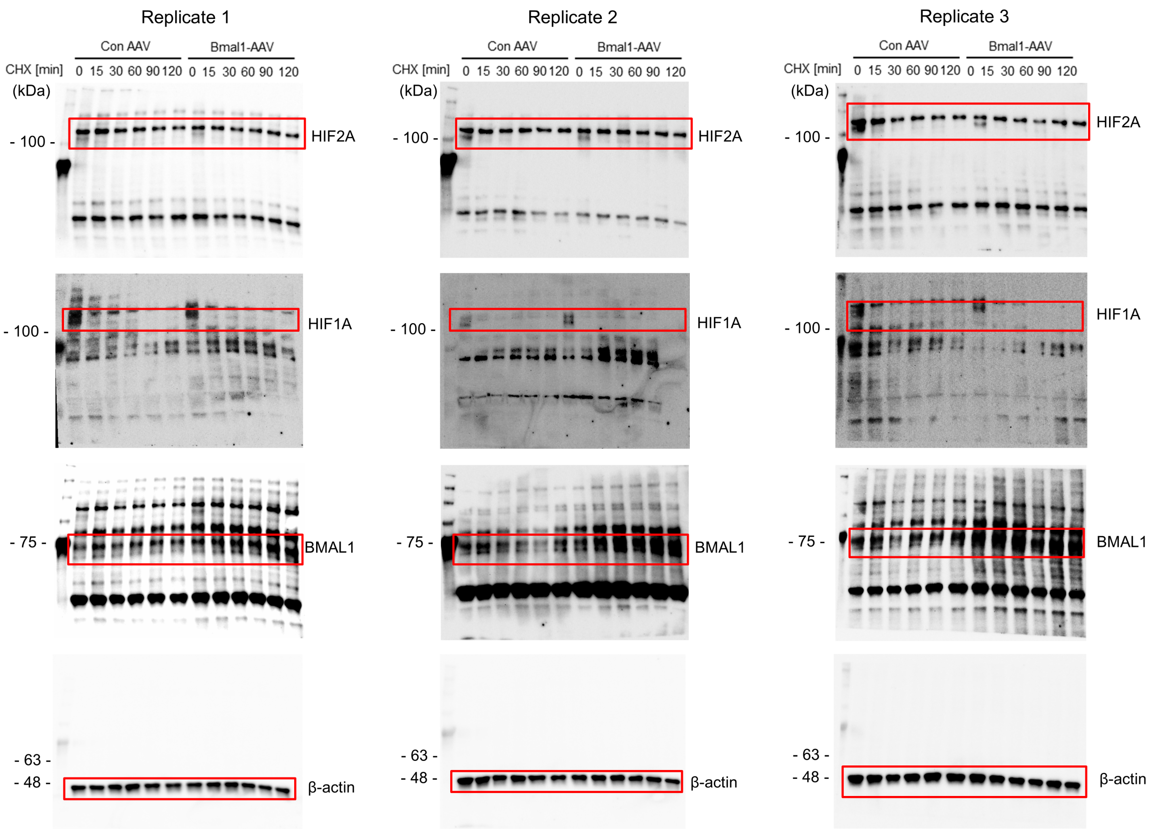

Uncropped WB images for Extended Data Fig. 7s. HCMs transduced with Bmal1-AAV or con AAV were exposed to 1% O_2 for 4 hours and treated with CHX to assess protein degradation (n = 3 biological replicates). Samples were run on the same gels, with β-actin used as the loading control.

Extended Data Fig. 7u

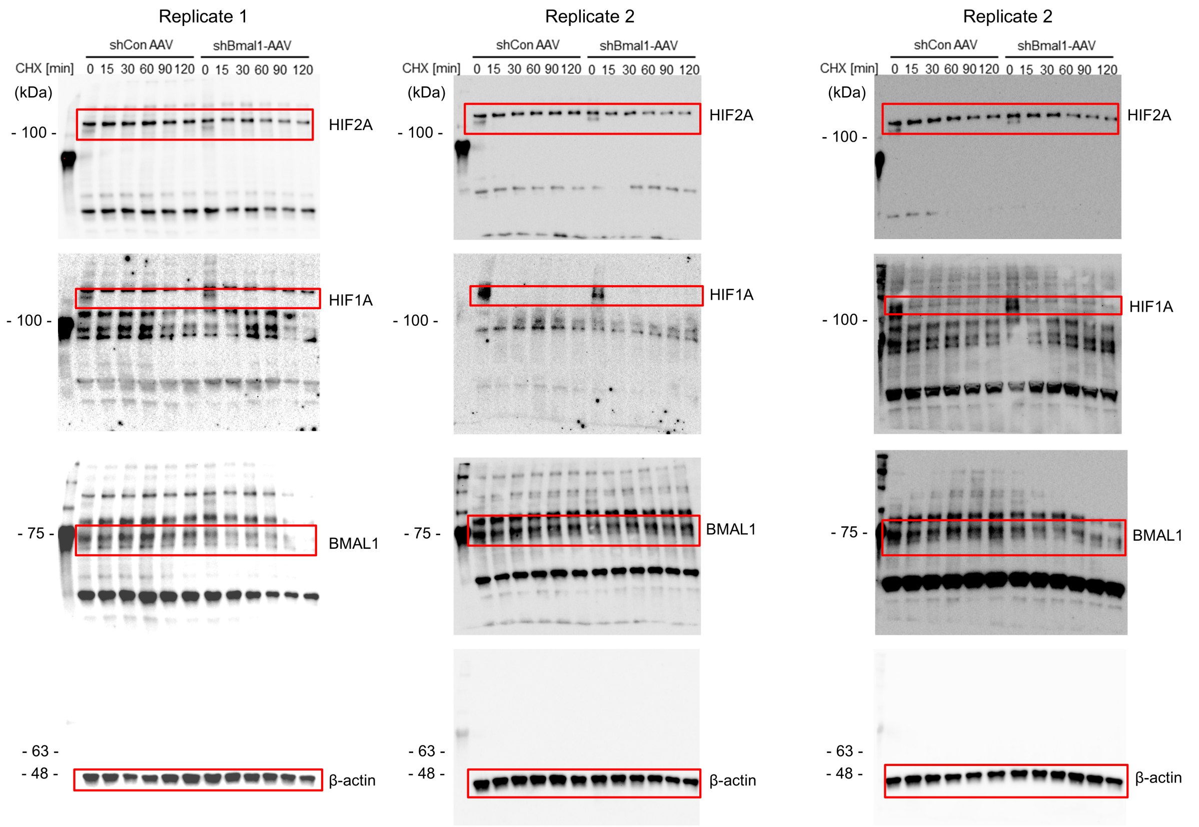

Uncropped WB images for Extended Data Fig. 7u. HCMs transduced with shBmal1-AAV or shCon AAV were exposed to 1% O_2 for 4 hours and treated with CHX to assess protein degradation (n = 3 biological replicates). Samples were run on the same gels, with β-actin used as the loading control.

Extended Data Fig. 7w (WB for HIF2A input was performed on a single membrane, which was then cut into three parts for the three replicates)

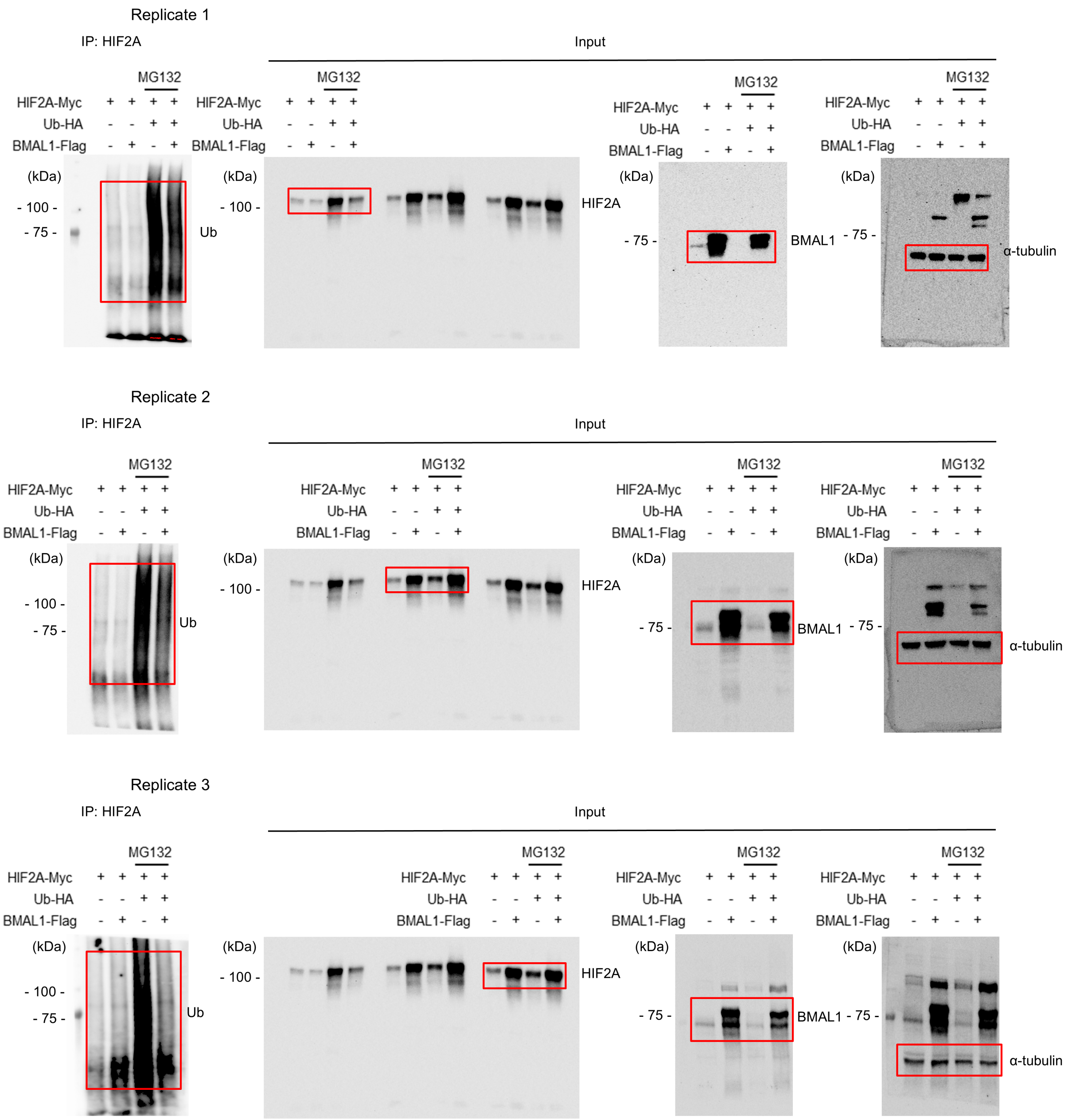

Uncropped WB images for Extended Data Fig. 7w. HEK293 cells transfected with HIF2A-Myc, Ub-HA, and BMAL1-Flag were treated with MG132 and subjected to HIF2A immunoprecipitation to analyze ubiquitination levels (n = 3 biological replicates). Samples were run on the same gels, with α-tubulin used as the loading control.

Extended Data Fig. 8d

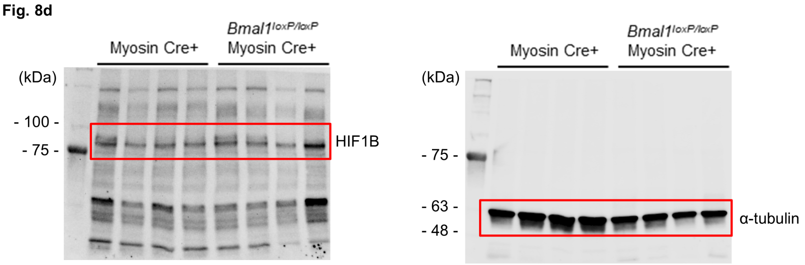

Uncropped WB images for Extended Data Fig. 8d. HIF1B protein levels in the AAR were assessed by Western blot on day 1 post-IRI in Myosin Cre+ and *Bmal1*$^{loxP/loxP}$ Myosin Cre+ mice (n = 4 biological replicates). Samples were run on the same gels, with α-tubulin used as the loading control.

Extended Data Fig. 8g (The β-actin blot is shared with **Supplementary Fig. 7c**)

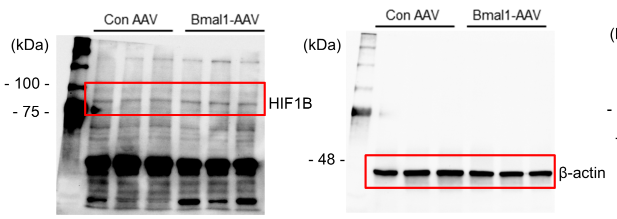

Extended Data Fig. 8j (The β-actin blot is shared with **Extended Data Fig. 7m**)

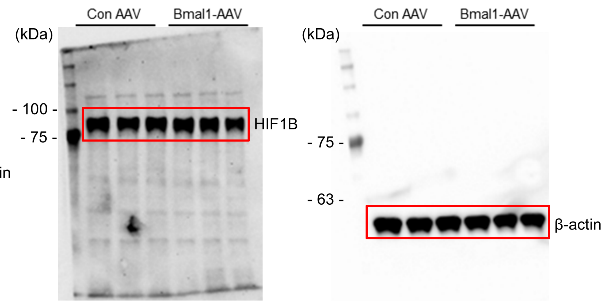

Uncropped WB images for Extended Data Fig. 8g and 8j. **g**, HIF1B protein levels in mouse hearts were assessed by Western blot 28 days after Bmal1-AAV or control AAV injection (n = 3 biological replicates). Samples were run on the same gels, with β-actin used as the loading control. The same samples in (**g**) were also used in **Supplementary Fig. 7c**, sharing the β-actin blot. **j**, HIF1B protein levels in HCMs transduced with Bmal1-AAV or control AAV under hypoxia were assessed by Western blot (n = 3 biological replicates). Samples were run on the same gels, with β-actin used as the loading control. The same samples used in (**j**) were also used in **Extended Data Fig. 7m**, sharing the β-actin blot.

Extended Data Fig. 8m (The β-actin blot is shared with **Extended Data Fig. 7p**)

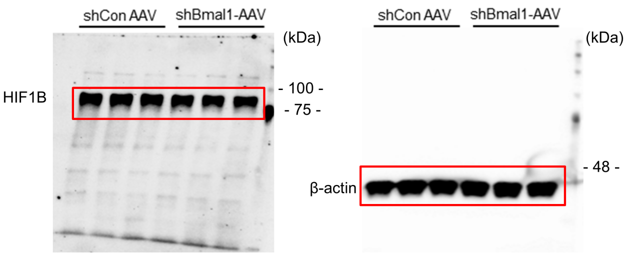

Uncropped WB images for Extended Data Fig. 8m. HIF1B protein levels in HCMs transduced with shBmal1-AAV or shcontrol AAV under hypoxia were assessed by Western blot (n = 3 biological replicates). Samples were run on the same gels, with β-actin used as the loading control. The same samples in (**m**) were also used in **Extended Data Fig. 7p**, sharing the β-actin blot.

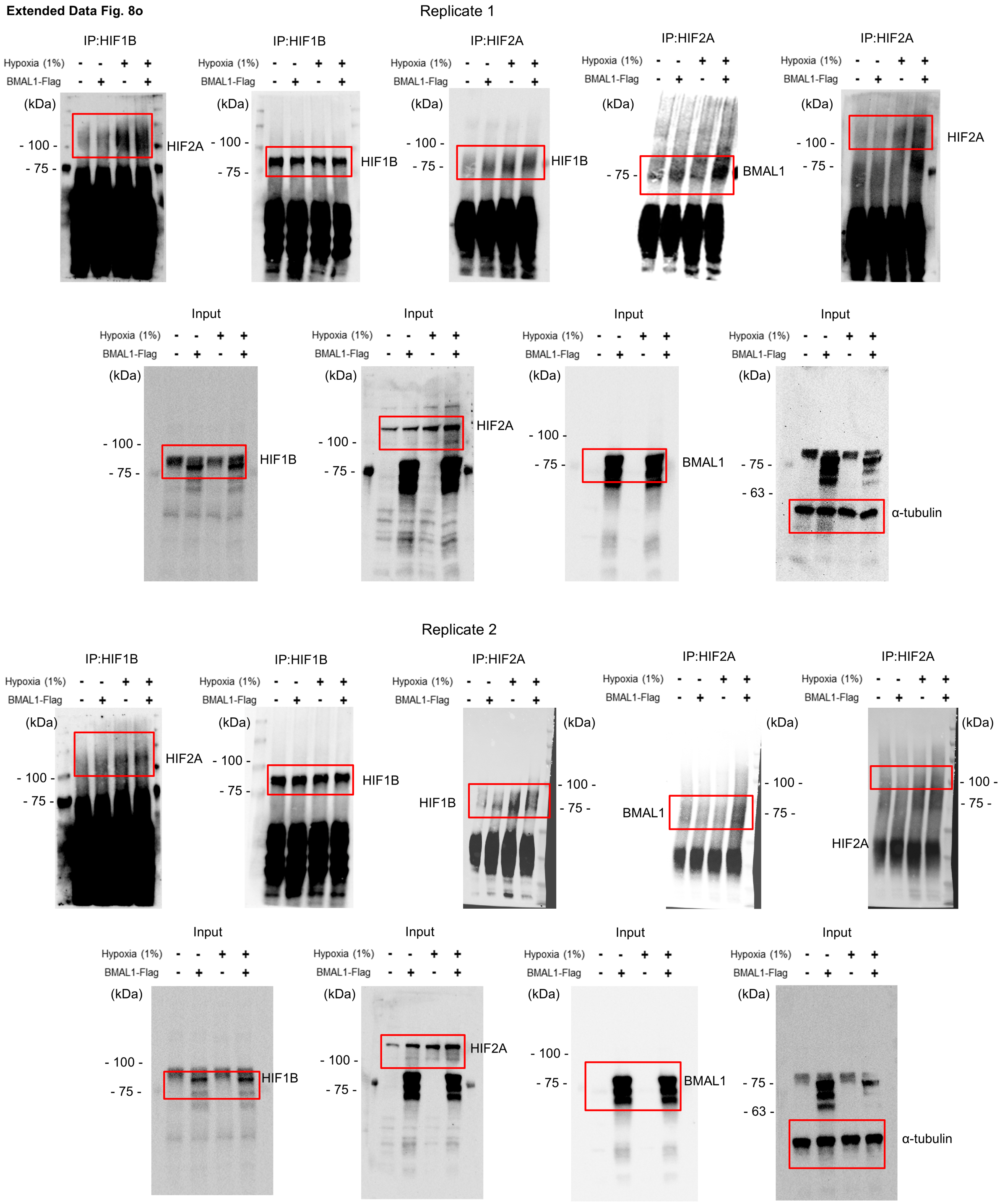

Uncropped WB images for Extended Data Fig. 8o. Immunoprecipitation was performed using HIF1B and HIF2A antibodies in HEK293 cells transfected with BMAL1-Flag or control plasmid under hypoxia or normoxia, followed by analysis by Western blot (n = 3 biological replicates). Samples were run on the same gels, with α-tubulin used as the loading control.

Extended Data Fig. 8o (continued)

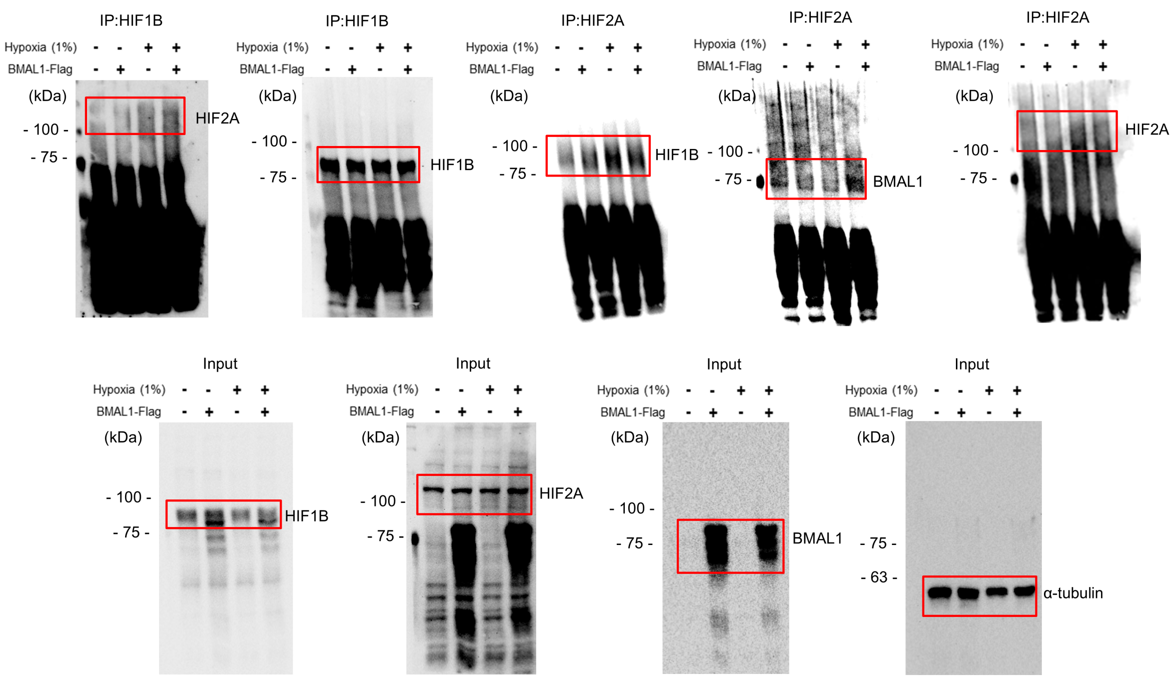

Extended Data Fig. 8s

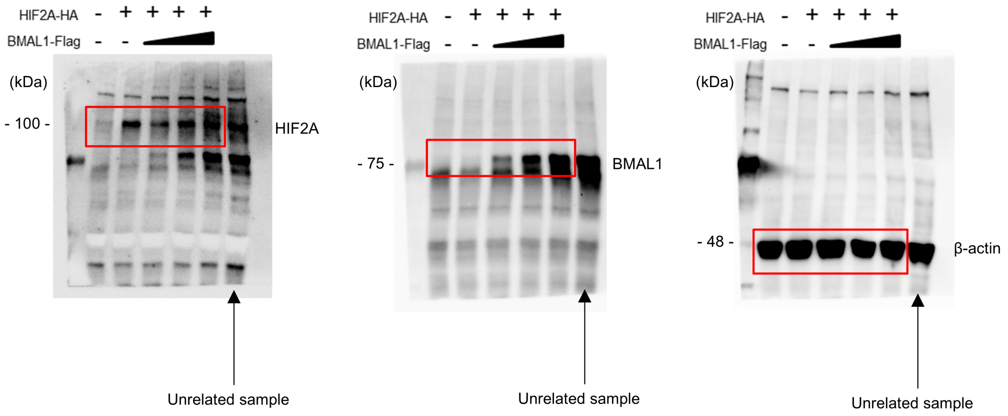

Uncropped WB images for Extended Data Fig. 8s. Luciferase assays in HEK293 cells transfected with BMAL1-Flag (0–200 nM), HIF2A-HA, and a luciferase reporter for the human *PGK1* promoter (n = 1). Samples were run on the same gels, with β-actin used as the loading control.

Supplementary Note 7: Uncropped gel blot images for Supplementary Figures

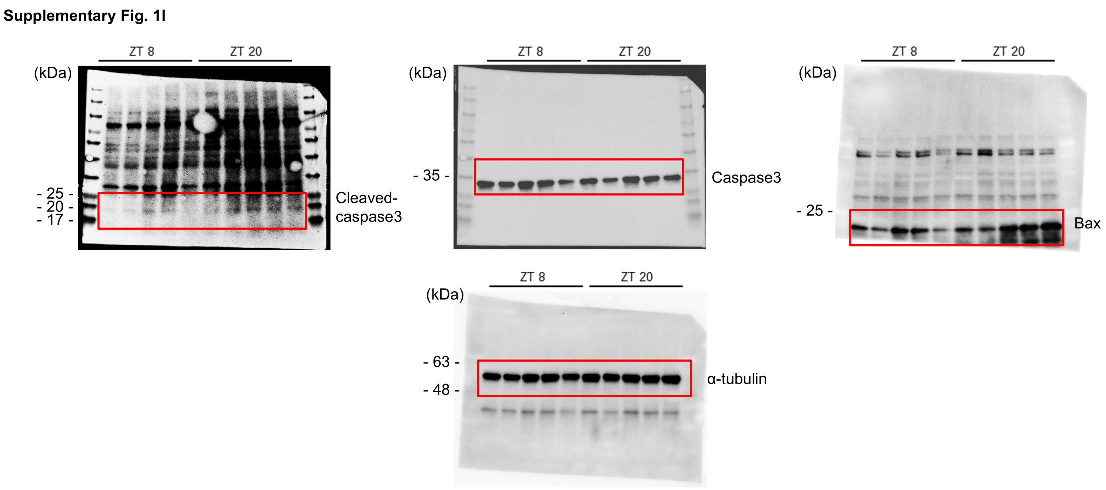

Uncropped WB images for Supplementary Fig. 1l. C57BL/6J mice were subjected to myocardial IRI at ZT8 or ZT20. Protein levels of cleaved-caspase3, caspase3, Bax, and α-tubulin in the AAR were measured in mouse hearts by Western blot analysis on day 3 post-MI (n = 5 biological replicates). Samples were run on the same gels, with α-tubulin used as the loading control.

Supplementary Fig. 3l

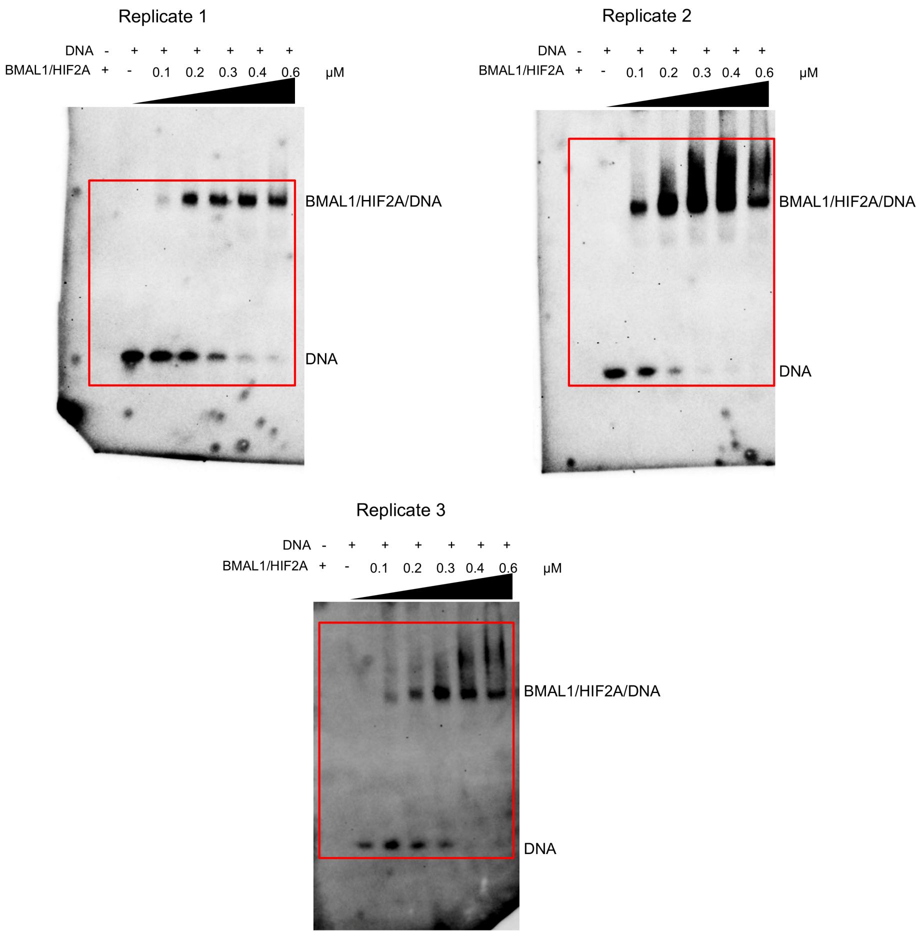

Uncropped WB images for Supplementary Fig. 3l. The electrophoretic mobility shift assay (EMSA) reveals the DNA binding capacity of the BMAL1/HIF2A heterodimer. DNA was labeled by biotin and detected using HRP-conjugated streptavidin with chemiluminescent substrates (n = 3 biological replicates).

Supplementary Fig. 5c

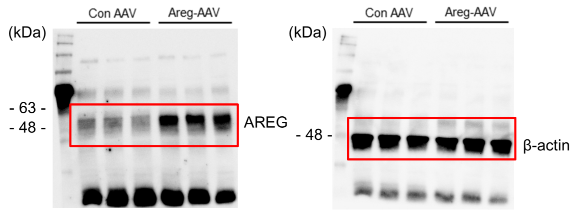

Supplementary Fig. 7c (β-actin blot is shared with **Extended Data Fig. 8g**)

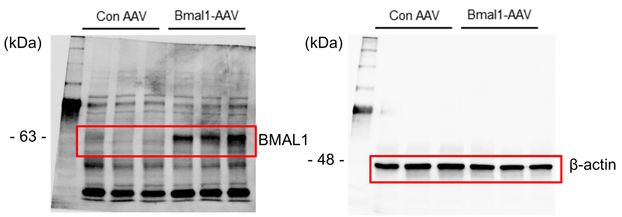

Uncropped WB images for Supplementary Fig. 5c. AREG expression in the hearts of mice on day 28 post-Areg-AAV or Con AAV administration was evaluated by Western blot analysis (n = 3 biological replicates). Samples were run on the same gels, with β-actin used as the loading control.

Uncropped WB images for Supplementary Fig. 7c. BMAL1 expression in mouse hearts on day 28 following Bmal1-AAV or Con AAV administration was evaluated by Western blot analysis (n = 3 biological replicates). Samples were run on the same gels, with β-actin used as

the loading control. Samples in **(c)** were also used in **Extended Data Fig. 8g**, sharing the β-actin blot.

Supplementary Fig. 9a

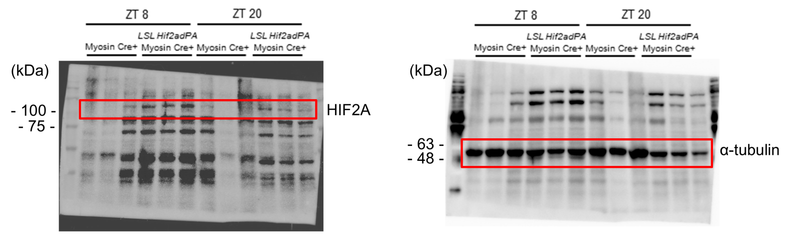

Uncropped WB images for Supplementary Fig. 9a. HIF2A protein levels in the hearts of *LSL-HIF2dPA* Myosin Cre+ mice and Myosin Cre+ mice were assessed by Western blot (n = 3 biological replicates). Samples were run on the same gels, with α-tubulin used as the loading control.
