# Supplementary material for: BMAL1–HIF2A heterodimer modulates circadian variations of myocardial injury
Source: Nature. 2025 Apr 23;641(8064):1017–28. doi: 10.1038/s41586-025-08898-z (PMC12095075; doi:10.1038/s41586-025-08898-z)
Supplement: Supplementary file 2 — Reporting Summary [file 41586_2025_8898_MOESM2_ESM.pdf]

nature portfolio

Corresponding author(s): Holger K. Eltzschig

Last updated by author(s): Feb 10, 2025

Reporting Summary

Nature Portfolio wishes to improve the reproducibility of the work that we publish. This form provides structure for consistency and transparency in reporting. For further information on Nature Portfolio policies, see our Editorial Policies and the Editorial Policy Checklist.

Statistics

For all statistical analyses, confirm that the following items are present in the figure legend, table legend, main text, or Methods section.

n/a	Confirmed	
☐	☒	The exact sample size (*n*) for each experimental group/condition, given as a discrete number and unit of measurement
☐	☒	A statement on whether measurements were taken from distinct samples or whether the same sample was measured repeatedly
☐	☒	The statistical test(s) used AND whether they are one- or two-sided<br>*Only common tests should be described solely by name; describe more complex techniques in the Methods section.*
☐	☒	A description of all covariates tested
☐	☒	A description of any assumptions or corrections, such as tests of normality and adjustment for multiple comparisons
☐	☒	A full description of the statistical parameters including central tendency (e.g. means) or other basic estimates (e.g. regression coefficient) AND variation (e.g. standard deviation) or associated estimates of uncertainty (e.g. confidence intervals)
☐	☒	For null hypothesis testing, the test statistic (e.g. *F*, *t*, *r*) with confidence intervals, effect sizes, degrees of freedom and *P* value noted<br>*Give P values as exact values whenever suitable.*
☒	☐	For Bayesian analysis, information on the choice of priors and Markov chain Monte Carlo settings
☒	☐	For hierarchical and complex designs, identification of the appropriate level for tests and full reporting of outcomes
☐	☒	Estimates of effect sizes (e.g. Cohen's *d*, Pearson's *r*), indicating how they were calculated

Our web collection on statistics for biologists contains articles on many of the points above.

Software and code

Policy information about availability of computer code

Data collection	EPU v2.10 (Thermofisher), Nikon Eclipse Ti2 confocal microscope (Nikon), Gen5 (Ver 3.12)
Data analysis	ImageJ software (Fiji v2.1.0), GraphPad Prism (v10.0), Vevo lab (FUJIFILM VisualSonics, v5.7.1), JASPAR (2022), Human Reference Interactome, STAR software (v2.7.10a), WebGestalt tool (v2019), TRRUST (v2), ChIPBase (v2), STRING database (v11.5), Cutadapt (v4.1), kallisto (v0.46.1), GENCODE human transcript (v44), DESeq2 (v1.34.0), GO annotation (v1.1), R package KEGGREST (v1.36.0), Cytoscape (v3.10.0), GEO2R (NCBI), NIS Element AR software (v6.10.01), MotionCor2 (v1.4.0), Gctf (v1.18), crYOLO (v1.10), RELION (v3.1), Cryosparc(v2.5), Chimera (v1.15), Chimera X (v1.7), Phenix (v1.21), Coot (v1.1), PYMOL (v2.5.5), cryoEF (v1.1.0), 3D FSC (v3.0), DeepEMhancer (v0.14), TraceDrawer (v.1.9.2).

For manuscripts utilizing custom algorithms or software that are central to the research but not yet described in published literature, software must be made available to editors and reviewers. We strongly encourage code deposition in a community repository (e.g. GitHub). See the Nature Portfolio guidelines for submitting code & software for further information.

Data

Policy information about availability of data

All manuscripts must include a data availability statement. This statement should provide the following information, where applicable:

- Accession codes, unique identifiers, or web links for publicly available datasets
- A description of any restrictions on data availability
- For clinical datasets or third party data, please ensure that the statement adheres to our policy

The mouse myocardial ischemia and reperfusion injury heart bulk RNA-seq data are available in the NCBI GEO database under accession number GSE255307. Analyses of the mouse RNA-seq data were performed using the Mus musculus reference genome assembly GRCm38 (Genome Reference Consortium Mouse Build 38), which is available from the NCBI under accession number GCF_000001635.20. Human surgical left ventricular bulk RNA-seq data are available under controlled access through the NIH database of Genotypes and Phenotypes (dbGaP) under accession number phs001679.v1.p1 (https://www.ncbi.nlm.nih.gov/projects/gap/cgi-bin/study.cgi?study_id=phs001679.v1.p1&phv=495897&phd=&pha=&pht=11851&phvf=&phdf=&phaf=&phtf=&dssp=1&consent=&temp=1). Access to these data is restricted due to privacy and ethical considerations. Requests must be submitted via dbGaP's Data Access Request (DAR) process. Interested researchers should apply for access through dbGaP by contacting the NIH Data Access Committee (DAC) and providing a detailed research proposal outlining the intended use of the data. The DAC typically reviews requests within two weeks, and access is granted subject to compliance with data use agreements (DUA). For further details on controlled access policies, data use agreements, and any additional restrictions, please refer to the dbGaP study page linked above. Questions regarding data access can be directed to the dbGaP helpdesk. The microarray assay for gene expression transcript levels in post-ischemic myocardium from Myosin Cre+ or Hif2aloxP/loxP Myosin Cre+ mice was re-analyzed using data obtained from the GEO database (accession number GSE67308). Cryo-EM map of the BMAL1/HIF2A/DNA complex was deposited to the EMDataBank with accession number EMD-43237. The corresponding atomic model was deposited to the RCSB Protein Data Bank with accession number 8VHG. Source data are provided with this paper.

Research involving human participants, their data, or biological material

Policy information about studies with human participants or human data. See also policy information about sex, gender (identity/presentation), and sexual orientation and race, ethnicity and racism.

Reporting on sex and gender	In our study, participants' sex data, based on information assigned at birth from hospital records, were collected at admission and are presented in Supplementary Table 5. Biological sex was included as a demographic variable to inform our investigation into transcriptomic profiles, which were designed to have universal applicability without requiring disaggregation by sex or gender. Self-reported gender identity was not collected or analyzed, as the study focused on its specific aims rather than exploring sex- or gender-based differences. Consent for reporting and sharing individual-level sex data was obtained during the hospital admission process.
Reporting on race, ethnicity, or other socially relevant groupings	None.
Population characteristics	The covariate-related population characteristics of the participants are detailed in Supplementary Table 5.
Recruitment	We examined samples from a prospective study of myocardial injury in humans during cardiac surgery (clinicaltrials.gov: NCT00281164). The study population consisted of consecutive patients (aged ≥20 years) with aortic stenosis referred to our cardiovascular surgery department at Brigham and Women's Hospital (MA, USA) for aortic valve replacement (with or without coronary artery bypass graft) between Jan 1, 2009, and Dec 31, 2014. Patients enrolled in a concurrent drug or device trial were excluded. This ongoing study involved 56 patients in the morning (samples collected between 8:00 am-12:00 pm, median time 10:32 am) and 17 patients who underwent the same procedure in the afternoon (samples collected between 3:00-9:00 pm, median time 5:15 pm). Patients whose surgery fell outside of these time periods were excluded from the analysis. The ethics committee of our institution approved the protocol, and written informed consent was obtained from all patients.
Ethics oversight	Committee for the Protection of Human Subjects from Research Risks of Brigham and Women's Hospital.

Note that full information on the approval of the study protocol must also be provided in the manuscript.

Field-specific reporting

Please select the one below that is the best fit for your research. If you are not sure, read the appropriate sections before making your selection.

☒ Life sciences ☐ Behavioural & social sciences ☐ Ecological, evolutionary & environmental sciences

For a reference copy of the document with all sections, see nature.com/documents/nr-reporting-summary-flat.pdf

Life sciences study design

All studies must disclose on these points even when the disclosure is negative.

Sample size	For all experiments, we employed a sample size of three or more biological replicates, guided by previous data/experiments from Eltzschig's lab to ensure adequate statistical power. Additionally, power analysis was performed to estimate the number of experimental mice.

Data exclusions	Data was not excluded, unless suggested via statistical testing (GraphPad, Identification of Outliers, ROUT method, Q=1%).
Replication	Three or more independent biological replicates were performed for all experiments, and all attempts at replication were successful, yielding consistent and reproducible results.
Randomization	Mice were randomly allocated into different experiment groups. For human RNA-seq data, randomisation was not relevant due to the study design where human left ventricular tissues from distinct time windows were used on availability.
Blinding	Blinding was implemented for the individuals analyzing the data, including those calculating infarct size and measuring serum troponin I levels, to ensure unbiased data interpretation. However, blinding the surgeons performing the surgeries was logistically challenging due to the nature of the procedures. Despite this, the surgeries were carried out by experienced surgeons, ensuring consistency. To confirm the similarity of conditions, we calculated the AAR/LV, which showed comparable values across ZT and treatment/vehicle groups. This suggests that any differences observed in the study are likely due to experimental conditions rather than variations in the surgical procedure. All ultrasound imaging acquisitions and subsequent offline measurements were conducted by a single investigator who was blinded to the grouping of the animals.

Reporting for specific materials, systems and methods

We require information from authors about some types of materials, experimental systems and methods used in many studies. Here, indicate whether each material, system or method listed is relevant to your study. If you are not sure if a list item applies to your research, read the appropriate section before selecting a response.

Materials & experimental systems

n/a	Involved in the study
☐	☒ Antibodies
☐	☒ Eukaryotic cell lines
☒	☐ Palaeontology and archaeology
☐	☒ Animals and other organisms
☐	☒ Clinical data
☒	☐ Dual use research of concern
☒	☐ Plants

Methods

n/a	Involved in the study
☒	☐ ChIP-seq
☒	☐ Flow cytometry
☒	☐ MRI-based neuroimaging

Antibodies

Antibodies used	Rabbit polyclonal anti-HIF1A (Novus Biologicals, Cat # NB100-479); Mouse monoclonal anti-HIF1A (Novus Biologicals, Cat # NB100-105, H1alpha67); Rabbit monoclonal anti-HIF1A (Bethyl Laboratories, Cat # 700-001, BL-124-3F7); Rabbit polyclonal anti-HIF2A (Novus Biologicals, Cat # NB100-122); Mouse monoclonal anti-HIF2A (Novus Biologicals, Cat # NB100-132, ep190b); Rabbit monoclonal anti-HIF2A (Bethyl Laboratories, Cat # 700-003, BL-95-1A2); Rabbit monoclonal anti-HIF1B (Cell Signaling Technology, Cat # 5537, D28F3); Rabbit monoclonal anti-BMAL1 (Cell Signaling Technology, Cat # 14020, D2L7G); Rabbit polyclonal anti-BMAL1 (Abcam, Cat # ab3350); Rabbit monoclonal anti-CLOCK (Cell Signaling Technology, Cat # 5157, D45B10); Rabbit polyclonal anti-RORα (Abcam, Cat # ab60134); Mouse monoclonal anti-AREG (Santa Cruz Biotechnology, Cat # sc-74501, G-4); Rabbit polyclonal anti-caspase-3 (Cell Signaling Technology, Cat # 9662); Rabbit polyclonal anti-cleaved caspase-3 (Cell Signaling Technology, Cat # 9661); Rabbit polyclonal anti-Bax (Cell Signaling Technology, Cat # 2772); Mouse monoclonal anti-FLAG (Sigma-Aldrich, Cat # F1804, M2); Rabbit monoclonal anti-Lamin B1 (Cell Signaling Technology, Cat # 12586, D4Q4Z); Rabbit polyclonal anti-TBP (Cell Signaling Technology, Cat # 8515); Mouse monoclonal anti-Lamin A/C (Cell Signaling Technology; Cat # 4777, 4C11); Mouse monoclonal anti-Ubiquitin (Santa Cruz, Cat # 8017, P4D1); Rabbit polyclonal anti-α-tubulin (Cell Signaling Technology, Cat # 2144); Mouse monoclonal anti-β-actin (Santa Cruz, Cat # 47778); Rabbit monoclonal IgG (Abcam, Cat # ab172730, EPR25A); Mouse monoclonal IgG (Cell Signaling Technology, Cat # 5415, G3A1); Rabbit polyclonal anti-α-sarcomeric (Abcam, Cat # ab137346); Rabbit monoclonal anti-vimentin (Cell Signaling Technology, Cat # 5741); Rabbit monoclonal anti-α-smooth muscle actin (Cell Signaling Technology, Cat # 19245, D4K9N); Alexa Fluor 488 conjugated WGA (ThermoFisher Scientific, Cat # W11261); Mouse monoclonal anti-His (ThermoFisher Scientific, Cat # MA1-21315, HIS.H8); Mouse monoclonal anti-GST (Genscript, Cat # A00865, 2F10B9); Alexa Fluor® 488-AffiniPure Goat Anti-Mouse IgG (H+L) (Jackson Immuno Research Laboratories, Cat # 115-545-062); Alexa Fluor® 594 AffiniPure Goat Anti-Rabbit IgG (H+L) (Jackson Immuno Research Laboratories, Cat # 115-585-144).
Validation	All antibodies used in this study were commercially developed and validated by the companies.<br><br>Rabbit polyclonal anti-HIF1A (Novus Biologicals, Cat # NB100-479), https://www.novusbio.com/products/hif-1-alpha-antibody_nb100-479?srsltid=AfmBOooHqExEKyTWCCQaLnJWu9oUfB2PkwIjB_jAvGq314yxl5l5KQm-<br>Mouse monoclonal anti-HIF1A (Novus Biologicals, Cat # NB100-105, H1alpha67), https://www.novusbio.com/products/hif-1-alpha-antibody-h1alpha67_nb100-105?srsltid=AfmBOoo9yujYZTK6W_vPivKCFsVSsIiuyWTqJZsHIslahj333D7weNJG<br>Rabbit monoclonal anti-HIF1A (Bethyl Laboratories, Cat # 700-001, BL-124-3F7), https://www.fortislife.com/products/primary-antibodies/rabbit-anti-hif1-alpha-recombinant-monoclonal-antibody-bl-124-3f7/BETHYL-A700-001<br>Rabbit polyclonal anti-HIF2A (Novus Biologicals, Cat # NB100-122), https://www.novusbio.com/products/hif-2-alpha-epas1-antibody_nb100-122?srsltid=AfmBOorJWrM2PUWCRCZ2QlCHB1yFlhcog9l9a_c8Zc7KWXCuOPsJFnJZ<br>Mouse monoclonal anti-HIF2A (Novus Biologicals, Cat # NB100-132, ep190b), https://www.novusbio.com/products/hif-2-alpha-epas1-antibody-ep190b_nb100-132?srsltid=AfmBOoqJ-r429Csf8bfly5oZmx9LTNviLGyfY-TULkRB8jE8UAllRPd1<br>Rabbit monoclonal anti-HIF2A (Bethyl Laboratories, Cat # 700-003, BL-95-1A2), https://www.fortislife.com/products/primary-

antibodies/rabbit-anti-hif2-alpha-recombinant-monoclonal-antibody-bl-95-1a2/BETHYL-A700-003
Rabbit monoclonal anti-HIF1B (Cell Signaling Technology, Cat # 5537, D28F3), https://www.cellsignal.com/products/primary-antibodies/hif-1b-arnt-d28f3-xp-rabbit-mab/5537?srsltid=AfmBOopRYtRtwa6pVhjKJAJVeVbj8R8JStwQc93anKZFZxDdXa1gemtd
Rabbit monoclonal anti-BMAL1 (Cell Signaling Technology, Cat # 14020, D2L7G), https://www.cellsignal.com/products/primary-antibodies/bmal1-d2l7g-rabbit-mab/14020?srsltid=AfmBOop34mdRT3kuyDYa0W47b-861uJvVsdAwZithAj1HxbCpX61LMGz
Rabbit polyclonal anti-BMAL1 (Abcam, Cat # ab3350), https://www.abcam.com/en-us/products/primary-antibodies/bmal1-antibody-ab3350?srsltid=AfmBOorZMLJnwYGCXPUx437nH7pdiAdE6ssweKU4tNH6g1Jso86QFCiJ
Rabbit monoclonal anti-CLOCK (Cell Signaling Technology, Cat # 5157, D45B10), https://www.cellsignal.com/products/primary-antibodies/clock-d45b10-rabbit-mab/5157?srsltid=AfmBOorJ26Z8uqzmdRLskxwJi8NpMrMuaC-khB58nSJtBQ9iZoE4F7q_
Rabbit polyclonal anti-RORα (Abcam, Cat # ab60134), https://www.abcam.com/en-us/products/primary-antibodies/ror-alpha-rora-antibody-ab70061?srsltid=AfmBOopFb8ARh2A4fXQBQyp2NR3RnF7UXzvVvK6hzIVr9D1Lih287w84
Mouse monoclonal anti-AREG (Santa Cruz Biotechnology, Cat # sc-74501, G-4), https://www.scbt.com/p/amphiregulin-antibody-g-4?srsltid=AfmBOorr0Slg00pu59cc8TpfYuBDRF682IZrU-if7pXiWWy1T8XQXumX
Rabbit polyclonal anti-caspase-3 (Cell Signaling Technology, Cat # 9662), https://www.cellsignal.com/products/primary-antibodies/caspase-3-antibody/9662?srsltid=AfmBOorycucfVz0gKsfympZ091CCwQ2z8Ok9RvzofSuj5O7oU0t0bY_p
Rabbit polyclonal anti-cleaved caspase-3 (Cell Signaling Technology, Cat # 9661), https://www.cellsignal.com/products/primary-antibodies/cleaved-caspase-3-asp175-antibody/9661?srsltid=AfmBOooCjnHunxcYG9tsBTy8c0cTSL5B6n6biaszMlIDRJSuTfLdsSYj
Rabbit polyclonal anti-Bax (Cell Signaling Technology, Cat # 2772), https://www.cellsignal.com/products/primary-antibodies/bax-antibody/2772?srsltid=AfmBOoov5fxcHDm4_xIFcha6Mzovg9PEMO4PsYm_GZjzmlyTzSKinbnY
Mouse monoclonal anti-FLAG (Sigma-Aldrich, Cat # F1804, M2), https://www.sigmaaldrich.com/US/en/search/f1804?focus=products&page=1&perpage=30&sort=relevance&term=f1804&type=product
Rabbit monoclonal anti-Lamin B1 (Cell Signaling Technology, Cat # 12586, D4Q4Z), https://www.cellsignal.com/products/primary-antibodies/lamin-b1-d4q4z-rabbit-mab/12586?srsltid=AfmBOoqMtO5sYN9sCsrOMCKT0HrX6vgMPz3Ixanstm3VtdNIih-Izmpj
Rabbit polyclonal anti-TBP (Cell Signaling Technology, Cat # 8515), https://www.cellsignal.com/products/primary-antibodies/tbp-antibody/8515?srsltid=AfmBOopSsqZ9n_vERdBJ0F6A8lZQP8R-4jS71URgSk8dKv_ysfo08Sn5
Mouse monoclonal anti-Lamin A/C (Cell Signaling Technology; Cat # 4777, 4C11), https://www.cellsignal.com/products/primary-antibodies/lamin-a-c-4c11-mouse-mab/4777?srsltid=AfmBOor0NNyu-u2TqKCM236s7OolSVZ_MJUHg8jZRLgSeN6AwmJZpVe7
Mouse monoclonal anti-Ubiquitin (Santa Cruz, Cat # 8017, P4D1), https://www.scbt.com/p/ubiquitin-antibody-p4d1?srsltid=AfmBOoqmotBs1SIKUZAkozv5von8CksIgKtXwiiDaOrLE8aEMcS927tL
Rabbit polyclonal anti-α-tubulin (Cell Signaling Technology, Cat # 2144), https://www.cellsignal.com/products/primary-antibodies/a-tubulin-antibody/2144?srsltid=AfmBOopiz8P5aWfUQ_XJlSwUaDht7MoJxqKKAAd-wuP4FgrXNDjNj6hF
Mouse monoclonal anti-β-actin (Santa Cruz, Cat # 47778, C4), https://www.scbt.com/p/beta-actin-antibody-c4?gad_source=1&gclid=CjwKCAiAh6y9BhBREiwApBLHC0l9SZdsM0Ae7fWAL_r7hlGymN7oJjk0sOZcxW7WPFwUx91gbFfPXBoCqmIQAvD_BwE
Rabbit monoclonal IgG (Abcam, Cat # ab172730, EPR25A), https://www.abcam.com/en-us/products/primary-antibodies/rabbit-igg-monoclonal-epr25a-isotype-control-ab172730?srsltid=AfmBOoqslItYqjtHZl1qusyj-h2rbbibvAiO4P9WXAPjPgBYAANaXy9U
Mouse monoclonal IgG (Cell Signaling Technology, Cat # 5415, G3A1), https://www.cellsignal.com/products/primary-antibodies/mouse-g3a1-mab-igg1-isotype-control/5415?srsltid=AfmBOorqNigUFdCUrrsnFWp7eJzw0MxTCO2n7aYdRYcdJmICaOqPa7SK
Rabbit polyclonal anti-α-sarcomeric (Abcam, Cat # ab137346), https://www.abcam.com/en-us/products/primary-antibodies/sarcomeric-alpha-actinin-antibody-ab137346?srsltid=AfmBOorYs2J75JIRM0ZEJg0I4_CeWzUnD2Fop9k5qqwiHjg1aLMcK526
Rabbit monoclonal anti-vimentin (Cell Signaling Technology, Cat # 5741, D21H3), https://www.cellsignal.com/products/primary-antibodies/vimentin-d21h3-xp-rabbit-mab/5741?srsltid=AfmBOorKgUai3G5yCBIHw_rHz6VW31ME-lO3Pl7WTMZynWvhdNdJLrft
Rabbit monoclonal anti-α-smooth muscle actin (Cell Signaling Technology, Cat # 19245, D4K9N), https://www.cellsignal.com/products/primary-antibodies/a-smooth-muscle-actin-d4k9n-xp-rabbit-mab/19245?srsltid=AfmBOormmxyF7FzJAuwkmZDyzgbAfRuc85eMHxlFpJBu9noL1ViB1zOf
Alexa Fluor 488 conjugated WGA (ThermoFisher Scientific, Cat # W11261), https://www.thermofisher.com/order/catalog/product/W11261
Mouse monoclonal anti-His (ThermoFisher Scientific, Cat # MA1-21315, HIS.H8), https://www.thermofisher.com/antibody/product/6x-His-Tag-Antibody-clone-HIS-H8-Monoclonal/MA1-21315
Mouse monoclonal anti-GST (Genscript, Cat # A00865, 2F10B9), https://www.genscript.com/antibody/A00865-THE_GST_Antibody_mAb_Mouse.html
Alexa Fluor® 488-AffiniPure Goat Anti-Mouse IgG (H+L) (Jackson Immuno Research Laboratories, Cat # 115-545-062), https://www.jacksonimmuno.com/catalog/products/115-545-062
Alexa Fluor® 594 AffiniPure Goat Anti-Rabbit IgG (H+L) (Jackson Immuno Research Laboratories, Cat # 115-585-144), https://www.jacksonimmuno.com/catalog/products/111-585-144

Eukaryotic cell lines

Policy information about cell lines and Sex and Gender in Research

Cell line source(s)	HEK293 (ATCC, Cat # CRL-157), https://www.atcc.org/products/crl-1573<br>human primary cardiomyocytes (ScienCell Research Laboratories,Cat # 6200, Lot# 29258), https://sciencellonline.com/en/human-cardiac-myocytes/?srsltid=AfmBOoqK1dnnLdKdk5rwwknXus0WHwlvVbMxtBUzr65qx2zZ3DeyhWHk
Authentication	All cell lines and primary cells used in the study were authenticated by STR profiling, as performed by the manufacturers.
Mycoplasma contamination	The cell lines were tested for mycoplasma by the manufacturers and were subsequently tested monthly for potential contamination. All mycoplasma contamination tests yielded negative results.
Commonly misidentified lines<br>(See ICLAC register)	None.

Animals and other research organisms

Policy information about studies involving animals; ARRIVE guidelines recommended for reporting animal research, and Sex and Gender in Research

Laboratory animals	To generate cardiac myocyte-specific deletion, Myosin Cre+ (STOCK A1cfTg[Myh6–cre/Esr1*]1Jmk/J, The Jackson Laboratory, RRID:IMSR_JAX:005650), Hif1aloxP/loxP (B6.129-Hif1atm3Rsjo/J, The Jackson Laboratory, RRID:IMSR_JAX:007561), Hif2aloxP/loxP (STOCK Epas1tm1Mcs/J, The Jackson Laboratory, RRID:IMSR_JAX:008407), LSL-Hif2dPA (STOCK Gt(ROSA)26Sortm4(HIF2A*)Kael/J, Jackson Laboratory, RRID: IMSR_JAX:009674), and Bmal1loxP/loxP (B6.129S4(Cg)-Bmal1tm1Weit/J, The Jackson Laboratory, RRID:IMSR_JAX:007668) mice, aged 8 weeks, were purchased from The Jackson Laboratory (Bar Harbor, ME) and crossbred. For the induction of Cre-recombinase activity, mice received an i.p. injection of tamoxifen at a dosage of 1 mg/day for five consecutive days. A recovery period of seven days was allowed following the final tamoxifen dose before proceeding with further experimental procedures. In these experiments, Myosin Cre+ mice served as controls. Additionally, Areg−/− mice (B6;129-Aregtm1Dle/Mmnc, MMRRC, MMRRC_011533-UNC). aged 8 weeks, were purchased from Mutant Mouse Resources & Research Centers (Chapel Hill, NC). To circumvent lactation difficulties observed in younger females, Areg−/− mice were bred using a heterozygous (Areg+/−) strategy. Control mice for these experiments were C57BL/6J (The Jackson Laboratory, 000664), aged 8 weeks, chosen for their same genetic background as the Areg−/− mice. Genotyping for all mouse strains was conducted by GeneTyper Inc. (NY, USA). Animal care was performed according to the guide for the care and use of laboratory animals of the National Institutes of Health. All experimental procedures were approved by the UTHealth Institutional Animal Care and Use Committee. The sample size was estimated based on published literature on previous murine models of myocardial ischemia and reperfusion injury. All mice were housed in a standard 12 h light:12 h darkness (L/D) photoperiod at 22°C with ad libitum access to a standard chow diet, and maintained at a humidity level of 40-60%.
Wild animals	None.
Reporting on sex	To address sex as a biological variable, both male and female mice, aged 8 to 16 weeks, underwent myocardial ischemia and reperfusion surgery. Our sex-specific analysis, which included assessments of infarct size, troponin levels, and cardiac function, revealed no significant differences between sexes.
Field-collected samples	None.
Ethics oversight	Animal Care and Use Committee (IACUC) for the University of Texas Health Science Center at Houston (UTHealth)

Note that full information on the approval of the study protocol must also be provided in the manuscript.

Clinical data

Policy information about clinical studies

All manuscripts should comply with the ICMJE guidelines for publication of clinical research and a completed CONSORT checklist must be included with all submissions.

Clinical trial registration	NCT00281164
Study protocol	https://clinicaltrials.gov/study/NCT01258231?term=NCT00281164&rank=1
Data collection	Data collection was completed at Brigham and Women's Hospital. Patients are currently being recruited at UT Southwestern Medical Center.
Outcomes	NA

Plants

Seed stocks	None.
Novel plant genotypes	None.
Authentication	None.
